# Regulation of ferroptosis in osteoarthritis and osteoarthritic chondrocytes by typical MicroRNAs in chondrocytes

**DOI:** 10.3389/fmed.2024.1478153

**Published:** 2024-11-05

**Authors:** Qingyuan Yu, Yanan Xiao, Mengqi Guan, Guohui Zhou, Xianshuai Zhang, Jianan Yu, Mingze Han, Wei Yang, Yan Wang, Zhenhua Li

**Affiliations:** ^1^Clinical College of Integrated Traditional Chinese and Western Medicine, Changchun University of Traditional Chinese Medicine, Changchun, Jilin, China; ^2^Scientific Research Center, China-Japan Friendship Hospital of Jilin University, Changchun, Jilin, China; ^3^Affiliated Hospital of Changchun University of Traditional Chinese Medicine, Changchun, Jilin, China

**Keywords:** osteoarthritis, ferroptosis, chondrocytes, circRNAs, lncRNAs, MicroRNAs, macrophages

## Abstract

Osteoarthritis (OA) is a progressive degenerative disorder impacting bones and joints, worsened by chronic inflammation, immune dysregulation, mechanical stress, metabolic disturbances, and various other contributing factors. The complex interplay of cartilage damage, loss, and impaired repair mechanisms remains a critical and formidable aspect of OA pathogenesis. At the genetic level, multiple genes have been implicated in the modulation of chondrocyte metabolism, displaying both promotive and inhibitory roles. Recent research has increasingly focused on the influence of non-coding RNAs in the regulation of distinct cell types within bone tissue in OA. In particular, an expanding body of evidence highlights the regulatory roles of microRNAs in OA chondrocytes. This review aims to consolidate the most relevant microRNAs associated with OA chondrocytes, as identified in recent studies, and to elucidate their involvement in chondrocyte metabolic processes and ferroptosis. Furthermore, this study explores the complex regulatory interactions between long non-coding RNAs (lncRNAs) and circular RNAs (circRNAs) in OA, with an emphasis on microRNA-mediated mechanisms. Finally, critical gaps in the current research are identified, offering strategic insights to advance the understanding of OA pathophysiology and guide therapeutic developments in this field.

## 1 Introduction

Osteoarthritis (OA) is a chronic degenerative disease that affects bones and joints, primarily in middle-aged and elderly populations. It leads to substantial pain, restricted movement, and financial burden due to joint discomfort and loss of mobility (1). The underlying causes of OA are multifactorial, potentially arising from disruptions in physiological processes such as redox homeostasis, anabolic and catabolic activities, and the balance between anti-inflammatory and pro-inflammatory responses (2). The pathology of OA is driven by various cellular changes, including the destabilization of cartilage metabolism, the pro-inflammatory transformation of macrophages, an imbalance between osteoblast and osteoclast activity, and impaired stem cell differentiation (3, 4). These cellular alterations underscore the importance of regulating these processes to effectively prevent and treat OA. Regulation of OA involves several critical mechanisms: maintaining chondrocyte proliferation and metabolic balance, promoting the polarization of macrophages towards anti-inflammatory states, coordinating the activities of osteoblasts and osteoclasts, and influencing the differentiation and proliferation of bone marrow-derived mesenchymal stem cells (BMSCs). MicroRNAs (miRNAs, miR), small non-coding RNAs 20 to 24 nucleotides long, play a pivotal role in these processes by binding to the 3′ untranslated region (3′UTR) of target genes, leading to mRNA degradation or the inhibition of translation. This regulatory function allows miRNAs to influence a wide range of cellular activities (5). Several miRNAs have been implicated in the regulation of cellular processes related to OA (6, 7). Their roles are often co-regulated by long non-coding RNAs (lncRNAs) and circular RNAs (circRNAs), with their expression being either upregulated or downregulated during the progression of OA. One specific mechanism of interest in OA pathogenesis is ferroptosis, an iron-dependent form of lipid peroxidation that results in plasma membrane damage and cell death. This process has been linked to cartilage damage and the worsening of OA (8). miRNAs have been identified as potential regulators of chondrocyte ferroptosis, suggesting their involvement in OA progression. This review systematically examines the regulatory roles of miRNAs in OA, focusing on their impact on various cell types in the bones and joints. It also explored the mechanisms through which miRNAs regulate OA, with a particular emphasis on their role in ferroptosis. Furthermore, this review summarizes miRNAs that are associated with ferroptosis and investigates their relationship with chondrocyte ferroptosis. The objective is to provide a more comprehensive understanding of OA pathogenesis and offer insights that may inform future clinical treatment strategies.

## 2 MicroRNAs are strongly associated with osteoarthritis

Osteoarthritis (OA) is a chronic, irreversible disorder of the bones and joints, characterized by progressive degeneration due to sustained inflammation and multiple homeostatic imbalances, culminating in pain and reduced mobility during advanced stages. While the precise pathogenesis of OA remains elusive, key factors contributing to its progression include the loss of chondrocytes and extracellular matrix from chronic injury, cellular senescence, and the development of advanced osteochondritis dissecans. Chondrocyte damage has been attributed to various triggers, including pharmacological agents, metabolic disorders, excessive mechanical stress, immune dysfunction, and inflammatory responses, all of which disrupt metabolic homeostasis and lead to both programmed and non-programmed chondrocyte cell death. Recent research has highlighted distinct alterations in microRNAs (miRNAs) within OA chondrocytes, suggesting their potential role in modulating OA progression. Over the past few years, studies have increasingly advanced our understanding of how miRNAs regulate key processes in OA, particularly their influence on cellular dynamics within bone and joint tissues. This review synthesizes the types of cell death modulated by specific miRNAs that drive critical cellular changes in bone and joint pathology (Supplementary Table 1), alongside an overview of cartilage-associated miRNA regulation (Supplementary Table 2), based on recent findings.

Research has demonstrated (9) that several microRNAs (miR-146a-5p, miR-34a-5p, miR-127-5p, and miR-140-5p) exhibit consistent expression changes in osteoarthritis (OA). These findings highlight their significant potential for use in the diagnosis and prognosis of OA (9). Considering the consistent differential expression of these four microRNAs (miRs) in osteoarthritis (OA), this review elucidates their specific regulatory mechanisms within the context of OA. Furthermore, it underscores the potential significance of targeting these miRs for future OA prevention and treatment strategies. The primary objective is to enhance the comprehension of osteoarthritis pathomechanisms and to support the advancement of therapeutic interventions in both scientific research and clinical practice.

### 2.1 MiR-140

MiR-140 plays a pivotal role in regulating cartilage homeostasis and is closely linked to aging-related OA. Among its isoforms, miR-140-3p is more abundantly expressed in cartilage compared to miR-140-5p (10). Both miR-140-3p and miR-140-5p exhibit markedly reduced expression in patients with aging-related OA (11–13). MiR-140-5P has been shown to inhibit genes involved in cartilage catabolism, such as MMP13 and ADAMTS5, while promoting the expression of genes associated with cartilage anabolism, including COL2A1, ACAN, OPN, and ALP (14). Similarly, miR-140-3P enhances the expression of CyclinD1 and Bcl-2, while suppressing Bax, p21, IL-6, IL-8, and TNF-α (15), thus countering chondrocyte senescence, inflammation, and apoptosis and stabilizing the cartilage matrix (16). Additionally, researchers have isolated cartilage-derived progenitor cells (CPCs), which possess the capacity to differentiate into cartilage and repair damaged tissue at injury sites. This regenerative ability is vital for maintaining cartilage integrity (17). However, in patients with advanced OA, a significant decrease in the expression of surface markers (CD166) and miR-140-5Pwas observed in CPCs. This downregulation is associated with increased osteoanabolic and catabolic activity, including the upregulation of matrix metalloproteinases (MMPs), ADAMTSs, and inflammatory markers, as well as disruptions in COL2A1 expression (17, 18). The Notch signaling pathway has been implicated in OA pathogenesis, with its components being generally upregulated in OA-affected tissues (19–21). Jagged1, a key ligand in the Notch pathway, is particularly elevated in OA chondrocytes (19). Studies using IL-1β-induced chondrocyte models and rat anterior cruciate ligament transection (ACLT) models have revealed that miR-140-5P may negatively interact with the Jagged1/Notch pathway (21, 22), suggesting that miR-140-5P might protect chondroprogenitor cells (CPCs) in OA by mitigating the detrimental effects of Notch signaling (23). These findings underscore the potential of miR-140-5P as a therapeutic target in preserving cartilage integrity and reducing OA progression. The transcription factor YY1 has been found to exhibit elevated expression levels in OA chondroprogenitor cells (OA CPCs), where it represses miR-140-5p transcriptionally. This suppression of miR-140-5p leads to a reduction in both the quantity and activity of CPCs through the YY1/miR-140-5p/Jagged1/Notch signaling axis (24). Targeting this axis to restore CPC function holds potential as a therapeutic strategy for OA by promoting chondrocyte replenishment and cartilage repair (18). Key components in this therapeutic approach include YY1, miR-140-5p, Notch receptors, and the ligand Jagged1.

Histone deacetylase 4 (HDAC4), a member of the histone deacetylase family, interacts with Runx2 and MEF2C to regulate genes such as Col-X, COMP, and Col-II, thereby inhibiting chondrocyte hypertrophy and stabilizing the cartilage matrix (25). Under hypoxic conditions, HIF-1α induces a significant increase in miR-140-3p expression, which subsequently upregulates SOX-9, COL2, ACAN, RUNX2, and SCX mRNAs, while downregulating COL1, COL6, COMP, TNC, and FMOD. This response protects the joints and delays the progression of OA (26). miR-140-5p also directly targets HDAC4, inhibiting its activity and regulating cartilage differentiation and proliferation (25). As a critical player in OA pathogenesis, miR-140-5p has become a focus for therapeutic intervention. Recent research has demonstrated that electroacupuncture stimulation (27) effectively inhibits the methylation of miR-140-5p and miR-146a, leading to their upregulation and modulation of downstream signaling pathways. This treatment reduces the expression of DNMT family proteins, inflammation-associated NF-κB, and cartilage damage-related SMAD3, likely through the regulation of upstream microRNAs (27). Additionally, miR-140-5p has been shown to inhibit PTEN (28, 29), promoting the anti-inflammatory phenotype of M2 macrophages, which subsequently polarizes adipose-derived stem cells (ADSCs) and activates the PI3K/AKT (30) and AKT/mTOR/HIF-1α (31)pathways. This activation enhances the osteogenic potential of ADSCs and supports bone regeneration (32). WNT5B, a ligand of the WNT pathway (33), has also been identified as a key regulator of cartilage homeostasis and differentiation (33, 34), playing a significant role in mitigating knee OA (KOA) progression (35). MiR-140-3p targets and inhibits WNT5B (36), and its suppression by circ-PREX1 has been linked to OA progression (36). In conclusion, miR-140-5p serves as a vital regulator in reducing OA-related damage, enhancing cartilage repair, and countering senescence. Given that chondrocyte injury and loss are central to the challenges of OA treatment, upregulation of miR-140-5p in CPCs within OA contexts could promote CPC stabilization and differentiation into chondrocytes, facilitating cartilage regeneration. However, further research is needed to fully elucidate its regulatory mechanisms. MiR-140-5p is thus poised to become a promising therapeutic target for repairing and regenerating damaged cartilage in OA.

### 2.2 MiR-127-5p

miR-127-5P has been shown to be downregulated in OA, where it plays a pivotal role as a regulator of cartilage metabolism (37) and acts as a key factor in countering OA progression (6, 38, 39). Research indicates that miR-127-5P directly targets the 3′ untranslated region (UTR) of osteopontin (OPN), inhibiting its expression (40). Additionally, miR-127-5P suppresses the PI3K/AKT signaling pathway, thus curbing abnormal chondrocyte overproliferation linked to OA progression (41). The long non-coding RNA (lncRNA) MALAT1 has been found to bind to and inhibit miR-127-5P, promoting excessive chondrocyte proliferation, a process that exacerbates OA (41).

In OA, CDH11 expression is elevated (42, 43), contributing to disease progression by increasing levels of inflammatory and catabolic markers, including MMP-13, IL-6, TNF-α, and ADAMTS-5 (44–46). Exosomes derived from bone marrow mesenchymal stem cells (BMSCs) containing miR-127-5P have been shown to target CDH11, inhibiting the Wnt3a/β-catenin pathway, thereby delaying OA development (47). DNM3OS acts as a negative regulator of miR-127-5P, promoting OA by activating the CDH11/Wnt3a/β-catenin/LEF-1 signaling axis. LEF-1’s positive correlation with DNM3OS suggests a feedback loop that worsens OA progression (48). In BMSCs, DNM3OS alleviates miR-127-5P’s suppression of GREM2, thereby hindering BMSC chondrogenic differentiation, particularly under hypoxic conditions (49). MiR-146-5P has been identified as a regulator that inhibits TLR4 (50) and LXN (51), protecting chondrocytes from apoptosis, inflammation, and oxidative stress. The knockdown of circSCAPER and circ_0002715 amplifies this protective effect by preventing the sponging of miR-127-5P (52, 53). Additionally, miR-127-5P targets and inhibits NAMPT, offering protection to chondrocytes against inflammation, apoptosis, and extracellular matrix degradation (54, 55). Circ_0128846 directly inhibits miR-127-5P, reducing its biological activity (56). Targeting circ_0128846 to release miR-127-5P presents a potential therapeutic strategy for OA treatment and prevention. Beyond chondrocytes, miR-127-5P promotes the osteogenic differentiation of BMSCs by interacting with the PTEN/AKT pathway (57) and inhibiting SPHK1 through targeting PDX1 (58), further enhancing osteogenesis (59). In contrast, circ_0134944 competitively downregulates miR-127-5P expression at PDX1, inhibiting osteogenic differentiation (60). In macrophages, miR-127-3p inhibits fatty acid synthase SCD1 (61) and regulates the NF-κB pathway (62), affecting macrophage proliferation and inflammation, although this mechanism has yet to be verified in synovial macrophages. In summary, miR-127-5p regulates key processes in chondrocytes, BMSCs, and macrophages, making it a vital inhibitor of OA progression. Targeting miR-127-5p to modulate downstream signaling pathways offers a promising therapeutic approach for OA. Further exploration of the regulatory relationships between miRNAs, circRNAs, and lncRNAs could provide deeper insights into OA pathogenesis and lead to the development of innovative treatment strategies.

### 2.3 MiR-34a-5P

miR-34a-5P demonstrates significant differential expression between normal and osteoarthritic cartilage, showing pronounced upregulation in osteoarthritic chondrocytes compared to healthy cartilage tissue (63). This overexpression correlates with elevated levels of catabolic markers (MMP13, ADAMTS5), inflammatory cytokines (IL-1, IL-6, TNF-α), and markers of chondrogenic hypertrophy (COL10A1), while simultaneously downregulating anabolic markers such as COL2A1 and ACAN. Additionally, an increase in pro-apoptotic markers, including caspase-3, caspase-9, and Bax, has been observed (63). The resulting inflammation, apoptosis, and degradation of the cartilage extracellular matontribute to the progression of osteoarthritic cartilage deterioration. Studies have shown that both miR-34a-5P and miR-125b-5P target and suppress SYVN1 expression, thereby promoting chronic inflammation, apoptosis, and other mechanisms involved in OA pathogenesis (64, 65). Moreover, lncRNA SNHG7 has been found to directly bind and inhibit miR-34a-5P, thereby alleviating its suppressive effect on SYVN1 and protecting chondrocytes from inflammatory and apoptotic damage (65). This regulatory interaction between microRNAs and lncRNAs in OA introduces a promising new area of research. Research has indicated that miR-34a-5P plays a pivotal role in promoting the osteogenic differentiation of bone marrow-derived mesenchymal stem cells (BMSCs) (66), primarily through the direct inhibition of HDAC1 (67), which subsequently activates estrogen receptor alpha (ER-α). This activation triggers an upregulation of osteogenic markers, including Runx2, alkaline phosphatase (ALP) (68), osteocalcin (OCN), and osteopontin (OPN). Additionally, the miR-34a-5P/smad2 axis has been shown to alleviate lipopolysaccharide (LPS)-induced suppression of osteogenic differentiation (69), while the miR-34c/SATB2 axis enhances osteoblast activity in osteoporotic mice (70), further highlighting the significant influence of miR-34a-5P on bone metabolism. lncRNA MALAT1 has emerged as a critical regulator in osteogenic differentiation and bone mineralization, acting through the negative regulation of miR-34a-5P, which in turn decreases Smad expression. This regulation impacts key osteogenic factors, such as Runx2, ALP, and OCN, thereby affecting bone mineralization processes (71). In osteoarthritic (OA) cartilage, SESN2, known to act as a leucine receptor, is significantly downregulated (72). SESN2 overexpression has been shown to inhibit mTOR pathway activity by promoting AMPK phosphorylation, thus facilitating cellular autophagy and protecting chondrocytes from senescence and extracellular matrix degradation (73). Notably, in cases of OA induced by hip dysplasia, miR-34a-5P has been identified as a suppressor of SESN2 activity, thereby impairing autophagy regulation (74). Oxidative stress and the excessive production of reactive oxygen species (ROS) are key drivers of chondrocyte damage in OA, playing a central role in disease progression. Emerging evidence suggests that ROS upregulates miR-34a-5P (75), which targets the SIRT1/P53 pathway, thereby promoting chondrocyte apoptosis and exacerbating tissue damage. SIRT1, through the acetylation of NRF2, enhances antioxidant defenses (76), while P53 has been found to further upregulate miR-34a-5P, establishing a feedback loop that amplifies P53-mediated apoptosis in chondrocytes (77). These findings underscore miR-34a-5P’s role in cartilage degradation and its contribution to the pathogenesis of OA. In summary, miR-34a-5P not only regulates osteogenic differentiation but also plays a critical role in the progression of OA by inhibiting extracellular matrix synthesis and autophagy, while promoting inflammation and apoptosis. This leads to cumulative chondrocyte damage and degeneration, facilitating OA progression. Targeting miR-34a-5P inhibition offers a promising therapeutic approach, while further exploration of the interactions between miR-34a-5P and other non-coding RNAs may provide deeper insights into the molecular mechanisms underlying OA pathology.

Moreover, it has been demonstrated that MiR-34a-5P can induce ferroptosis by targeting and inhibiting SIRT1 (78). However, it remains unclear whether similar regulatory mechanisms are present in osteoarthritic (OA) cartilage. Future research addressing this gap will be instrumental in expanding our understanding of MiR-mediated regulation of chondrocyte ferroptosis and its implications for OA progression. Additionally, MiR-34a-5P has been shown to be significantly upregulated in advanced OA synovial tissue (63). MiR-34-5P has been demonstrated to interact with ATF3, IL6, IL1B, and EGR1—an iron death-related gene identified in osteoarthritis (OA) synovial tissue—among others, potentially contributing to the pathogenesis of iron death (79). Notably, miR-155-5p (80, 81) may exhibit a similar function. In summary, targeting MiR-34-5P not only modulates the progression of OA in terms of inflammation, bone metabolism, and other factors, but its regulation of chondrocyte iron death also presents significant potential for targeted OA therapy.

### 2.4 MiR-146a-5P

The expression of miR-146a-5P in peripheral blood mononuclear cells and chondrocytes of patients with OA has been found to be significantly elevated compared to healthy controls (82). This microRNA is recognized as a key contributor to OA pathogenesis (83). Both *in vivo* and *ex vivo* studies have demonstrated that miR-146a-5P exacerbates OA through its pro-inflammatory and pro-apoptotic effects, as well as by destabilizing the cartilage matrix. Its mechanism likely involves enhancing P65 phosphorylation, which activates the NF-κB pathway, leading to the upregulation of pro-inflammatory cytokines such as IL-6 and TNF-α, alongside chondrolysis-associated factors like MMP13, thus promoting OA progression (83). Moreover, Prkg1, a key component of the nitric oxide (NO)/cGMP signaling pathway, has emerged as a potential therapeutic target of miR-146a-5P in OA treatment (83, 84). miR-146a-5P has also been implicated in activating the p65/NF-κB pathway by targeting TRAF6, which promotes inflammatory damage and IL-1β-induced chondrocyte apoptosis, further aggravating OA (85). In addition, resolvin D1, a mediator that supports lipid metabolism, has been shown to suppress the NF-κB-mediated pro-inflammatory pathway by inhibiting the transcription factor KLF5 (86), thereby attenuating inflammation (87, 88). However, miR-146a-5P, upregulated in OA, may hinder resolvin D1’s effects, exacerbating lipopolysaccharide (LPS)-induced macrophage inflammation (89). miR-146a-5P also targets NUMB, inhibiting its expression, which results in increased apoptosis and reduced autophagy in chondrocytes, contributing further to OA pathogenesis (60). Additionally, diminished POU2F1 expression in OA (90) is linked to IL-1β-induced cartilage matrix degradation, chondrocyte apoptosis, and inflammation (91), a process likely driven by the upregulation of miR-146a-5P, which suppresses POU2F1 expression (91). lncRNA FAM201A has been shown to counteract OA-associated inflammation and chondrocyte apoptosis by inhibiting miR-146a-5P and interacting with the POU2F1 promoter, leading to POU2F1 upregulation. This creates a positive feedback loop, as POU2F1 also enhances lncRNA FAM201A expression (91). Despite these insights, the downstream effectors in the lncRNA FAM201A/miR-146a-5P/POU2F1 regulatory axis remain unidentified, warranting further investigation. Nonetheless, it is well established that POU2F1 regulates TWIST1 expression *via* transcriptional mechanisms, thereby inhibiting the WNT pathway (90, 92–94). This regulatory function may contribute to OA, though its precise role in the process remains to be fully elucidated. The downregulation of BMPR2 in OA appears to accelerate disease progression, likely due to miR-146a-5P targeting and inhibiting BMPR2, thereby disrupting BMP signaling (95, 96). Notably, overexpression of the long non-coding RNA (lncRNA) MINCR has been shown to counteract this mechanism, suggesting that lncRNA MINCR may alleviate OA by mitigating the suppressive effects of miR-146a-5P on BMPR2 (97). Additionally, miR-1307-3p has been identified as another microRNA capable of targeting BMPR2 (98). Furthermore, miR-146a-5P serves as a key inhibitory regulator in bone formation (99), directly targeting SMAD4 and potentially inducing osteoblast apoptosis by upregulating pro-apoptotic genes such as caspase-3 and Bax (100). Recent studies have also highlighted the role of miR-146a-5P in damaging the mitochondrial oxidative respiratory chain *via* inflammatory pathways, leading to mitochondrial dysfunction and promoting apoptosis in MIN6 cells (101). Despite these findings, the precise regulatory mechanisms of miR-146a-5P concerning mitochondrial function in OA chondrocytes remain unclear. This underscores the need for further research into the interaction between microRNAs and mitochondrial dynamics, which may reveal novel targets for better understanding OA pathogenesis and developing more effective therapeutic strategies.

### 2.5 MiR-24

MiR-24 has been demonstrated to exhibit reduced expression levels in senescent or dysfunctional chondrocytes associated with osteoarthritis (OA), and it is linked to the capacity to mitigate cartilage damage and chondrocyte senescence (102). Prior research has indicated that C-myc, a gene implicated in the induction of inflammation and apoptosis in OA (103–105), can be targeted by MiR-24 to suppress its expression (106, 107). However, MiR-24 has been identified as a negative regulator of P16 and was shown early on to influence the chondrocyte senescence process (108). This underscores the significant regulatory role of MiR-24 in osteoarthritis (OA). Previous research has highlighted the substantial potential of synovial mesenchymal stem cells (SMSC) in chondrogenic differentiation (109–111). Recent investigations have demonstrated that a MiR-24/SMSC complex-associated hydrogel (MSOH) exhibits a promising therapeutic effect in the repair and differentiation of OA cartilage damage (102). MSOH effectively regulates cartilage homeostasis and facilitates the regeneration of osteoarthritic cartilage by modulating glycolytic pathways, enhancing the oxidative phosphorylation process, and mitigating chondrocyte senescence, inflammation, and ferroptosis, as evidenced by reductions in HMGB1 and p16ink4a levels. This regulatory mechanism may be attributed to the ability of MiR-24 to counteract senescence and promote chondrogenesis in synovial mesenchymal stem cells (SMSC) and chondrocytes by targeting and inhibiting the downstream effector TAOK1 (102). Additionally, it has been demonstrated that lncRNA C9ORF139 can act as a sponge for MiR-24, thereby modulating the expression of TAOK1 (112). Chondrocyte injury is a significant and persistent factor in the pathogenesis of osteoarthritis (OA). This study highlights the potential of microRNA-24 (MiR-24) in conjunction with stem cell-derived mesenchymal stem cells (SMSC) to facilitate the repair of damaged cartilage and mitigate chondrocyte senescence. Furthermore, it proposes a novel therapeutic direction for the management of OA. Importantly, this research represents a meaningful effort to translate the molecular insights regarding MiR-24 into clinical applications. These findings underscore the promising regulatory role of microRNAs in the context of OA and their potential for therapeutic intervention.

## 3 Mechanisms of action of other microRNAs associated with osteoarthritis

Damage and loss of articular cartilage are recognized as fundamental contributors to refractory osteoarthritis (OA). The etiological factors implicated in this process include inflammation, chondrocyte apoptosis, cartilage matrix degradation, and dysregulated autophagy (113, 114). This paper will examine the regulatory role of microRNAs (miRNAs) in OA chondrocytes, a topic that has gained significant attention in recent years, through these specific dimensions.

### 3.1 MicroRNA regulation of osteoarthritis cartilage metabolism

Previous research has indicated that miR-199a may inhibit early chondrogenesis by downregulating the expression of COMP, SOX9, and type II collagen (115). Additionally, miR-199a has been identified as a regulator of chondrogenic differentiation in stem cells *via* its targeting of SMAD1 (115). Moreover, miR-199a has been suggested to reduce the expression of the inflammatory marker COX-2, thereby providing protection to chondrocytes from inflammatory damage (116). Additionally, miR-199a-5p has been reported to alleviate OA symptoms by targeting MAPK4 (117). Recent studies have shown that miR-199a-5p may influence OA pathogenesis by targeting Gcnt2 and Fzd6 (118). Moreover, it has been proposed that miR-199a-5p exerts its effects by inhibiting the Indian hedgehog (Ihh) signaling pathway (119). miR-107-5p targets CASP3, protecting chondrocytes from apoptosis and extracellular matrix (ECM) degradation, thereby delaying OA progression. CircSEC24A has been identified as a negative regulator of miR-107-5p (120). Intra-articular injections of miR-81 in rats have been shown to suppress IL-16 expression, reducing MMP3 and MMP13 levels, and consequently, mitigating extracellular matrix damage and bone catabolism (121). Chondrocyte-derived exosomal miR-125 has been linked to osteogenic differentiation, influenced by sympathetic regulation, and is implicated in the pathophysiology of age-related OA (122). In contrast, miR-29b-3p negatively correlates with TGF-β1 and reduces chondrocyte numbers by inhibiting the TGF-β1/Smad signaling pathway (123). miR-4492 has been shown to modulate IL-18 production through the MEK/ERK signaling pathway, impacting OA cartilage (124). miR-204 targets and inhibits SP1-LRP1 signaling, disrupting the neural-cartilage interface and alleviating OA-associated pain (125). miR-81 has been proposed to contribute to cartilage homeostasis and the regeneration of damaged cartilage by targeting and inhibiting Rac2, which upregulates key anabolic factors such as SOX9, COL2A1, and ACAN, facilitating the differentiation of bone marrow-derived mesenchymal stem cells (BMSCs) into cartilage and promoting bone anabolism (126). miR-362-5p has been implicated in OA by inhibiting the differentiation of bone marrow-derived mesenchymal stem cells (BMSCs) into cartilage through the suppression of PLXNB1, reducing chondrocyte replenishment and exacerbating OA progression (127). miR-302c, by directly targeting and inhibiting TGFBR2, mitigates IL-1β-induced damage in chondrocytes (128), miR-136-5p regulates NAMPT activity, playing a role in OA progression through its modulation of this key enzyme (129).

### 3.2 Regulation of chondrocyte apoptosis by MicroRNAs

miR-539-3p has also been found to target and inhibit SOX9 and TGF-β1 (112), which results in a reduction in the chondrogenic differentiation of human adipose-derived stem cells (hASCs) and decreased expression of COL2A1 and ACAN (130). Furthermore, miR-539-3p has been shown to inhibit Runx2, thereby reducing chondrocyte apoptosis, inflammation, and extracellular matrix degradation in pediatric OA, ultimately alleviating the disease (131). miR-214-3p, present in synovial fibroblast-derived exosomes, has been shown to protect articular cartilage from inflammatory and apoptotic damage (132). This protection is likely mediated through the inhibition of FOXM1 expression, thereby reducing chondrocyte apoptosis (133). Recent studies further suggest that miR-214 targets and inhibits Bax and TRPV4, contributing to the suppression of chondrocyte apoptosis and injury (134). Research has shown that appropriate mechanical stress can upregulate miR-214 expression in chondrocytes, contributing to the maintenance of cartilage integrity (135). Similarly, the upregulation of miR-653-5p in OA cartilage has been demonstrated to attenuate IL-6 expression and inhibit JAK1 and STAT3 phosphorylation, which helps mitigate chondrocyte senescence and delay OA progression in a destabilization of the medial meniscus (DMM) rat model (136). miR-149-5p targets TRADD, reducing caspase-3, caspase-8, and TNF-α levels, which decreases chondrocyte apoptosis and inflammation (137). Similarly, has-miR-4282 was found to mitigate OA by modulating cellular pyroptosis through the targeting of the NF-κB/NLRP3 pathway (138). miR-506-3p, on the other hand, targets the PI3K/AKT/mTOR pathway, promoting chondrocyte apoptosis and contributing to OA pathogenesis (139). Conversely, the long non-coding RNA HOXA11-AS exhibits a negative correlation with miR-506-3p. miR-99a has been shown to inhibit FZD8, thus protecting spinal joint cells from IL-6 and TNF-α-induced apoptosis and inflammation (140). Moreover, miR-99a-5P has been found to negatively regulate TLR8, indirectly modulating the innate immune response and activating the PI3K/Akt pathway in OA chondrocytes, contributing to cartilage damage (141). Additionally, miR-320c targets CREB5, inhibiting the cAMP pathway and contributing to OA development (142), while miR-320a modulates the ERK/JNK/MAPK pathways by targeting DAZAPI, potentially delaying OA progression (143). Additionally, miR-502-5p inhibits TRAF2, reducing NF-κB activity, which protects chondrocytes from apoptosis and inflammatory damage caused by TNF-α and IL-1β, while also supporting cartilage anabolism (143). miR-203a-3p protects cartilage from LPS-induced apoptosis, pyroptosis, and oxidative stress by inhibiting the MYD88/NF-κB signaling pathway (144). miR-182-5p has been shown to bind to FGF9, resulting in a reduction in its levels, which subsequently promotes chondrocyte injury and apoptosis, exacerbating OA symptoms (145). Additionally, miR-3591-5p has been shown to mitigate the progression of OA by targeting and inhibiting PRKAA2. The demethylation of the miR-3591-5p precursor by FTO impairs the maturation of miR-3591-5p, promoting OA development through the suppression of its protective effects (146). In contrast, miR-155 contributes to OA pathogenesis by targeting SMAD2 and activating the NLRP3/Caspase-1 pathway, leading to chondrocyte pyroptosis (147). miR-155 also disrupts the PI3K/Akt signaling pathway by targeting PIK3R1, which exacerbates IL-1β-induced chondrocyte apoptosis and matrix degradation (148). Exosomal miR-3960, released from mesenchymal stem cells (MSCs), targets PHLDA2, reducing SDC1/Wnt/β-catenin pathway activity and protecting cartilage from OA-related damage (149).

### 3.3 MicroRNA regulation of osteoarthritis-related inflammation

miR-199-3p has been shown to mediate autophagy and suppress OA-related inflammation by reducing pro-inflammatory cytokines such as TNF-α, IL-6, and IL-1β, while also decreasing chondrocyte apoptosis through the inhibition of TCF4 and DNMT3A (150, 151). Furthermore, LINC00707, which is significantly upregulated in OA, may contribute to chondrocyte apoptosis by acting as a sponge for miR-199-3p (152). miR-26b-5p has been shown to induce cartilage damage and cellular senescence by inhibiting the TGF-β1-Smad2 signaling pathway, which occurs through the upregulation of asporin (153). Additionally, miR-26b-5p targets COL10A1 and TLR3, inhibiting the pro-inflammatory M1 macrophage phenotype and reducing inflammatory mediators such as IL-1β, IL-6, TNF-α, and PTGS2. At the same time, it promotes macrophage polarization towards the anti-inflammatory M2 phenotype, thus mitigating OA progression by reducing synovial inflammation and preserving cartilage integrity (154). miR-4738-3p has also been shown to mitigate OA-related inflammation by inhibiting the NF-κB signaling pathway and suppressing Col1A2 expression (155). Furthermore, the upregulation of miR-877-5p has been demonstrated to reduce IL-1β-induced inflammation and apoptosis in chondrocytes by inhibiting FOXM1 expression (156). miR-877-5p enhances SOX9 and collagen type II (COL II) expression, offering protective effects on the cartilage matrix (156). In osteoblasts, miR-877-5p targets and inhibits EIF4G2, facilitating osteoblast differentiation (157). Conversely, miR-350-3p has been found to promote synovial macrophage transformation into a pro-inflammatory phenotype, exacerbating inflammatory chondrocyte injury and OA pathogenesis. On the other hand, miR-350-5p, delivered *via* macrophage exosomes, inhibits NSD1-mediated H3K36 methylation in chondrocytes, facilitating OA progression (158). miR-515-5p protects against IL-1β-induced cartilage degradation by inhibiting TLR4 and suppressing NF-κB pathway activation, resulting in anti-inflammatory and anti-apoptotic effects (159). miR-577 reduces chondrocyte inflammation by downregulating MTF-1, while LINC01094 negatively regulates miR-577 (160). Similarly, miR-558 may exert analogous effects to miR-577 and is potentially regulated by Circ_0007482 (161). Finally, miR-98-5p has been shown to directly target and inhibit CASP3 expression, decreasing inflammatory mediators and pro-apoptotic factors such as caspase-3 and Bax, thereby offering protection to chondrocytes from inflammation, apoptosis, and extracellular matrix degradation (162). The overexpression of miR-98-5P has been demonstrated to reduce IL-1β-induced cartilage damage, offering protective effects against OA progression (162). The overexpression of miR-124-3p in OA chondrocytes has been found to inhibit the interaction between MALAT1 and KLF5, thereby reducing inflammation—marked by decreased IL-1β and IL-18 levels (163)—and preventing extracellular matrix degradation and chondrocyte pyroptosis through the suppression of CXCL11 transcription (164). This multifaceted regulatory mechanism ultimately contributes to the mitigation of OA pathogenesis (165). miR-93-5P, encapsulated in exosomes derived from adipose-derived stem cells (ADSCs), has been demonstrated to inhibit ADAMTS9, leading to the activation of the PI3K/AKT/mTOR signaling pathway. This activation counteracts IL-1β-induced inflammation and apoptosis, providing a protective effect on chondrocytes (166). In contrast, long non-coding RNA (lncRNA) CASC2 has been identified as a negative regulator of miR-93-5P, thereby mitigating its beneficial effects in OA (167). miR-149 has been shown to play a protective role in OA by inhibiting the PI3K/AKT signaling pathway through targeted suppression of VCAM-1, which is associated with reduced inflammation and chondrocyte apoptosis (168). Finally, miR-150-5p has been found to attenuate VCAM-1 expression by inhibiting the activity of the lncRNA XIST. This inhibition reduces monocyte recruitment and adhesion in OA synovial tissues, potentially exerting a palliative effect by decreasing inflammation and mitigating OA progression (169). The miR-150-5p/AKT axis has been identified as a key regulator in the progression of OA and is negatively regulated by the long non-coding RNA MALAT1 (170). Similarly, miR-5701 has been shown to suppress VCAM-1 activity, attenuating the inflammatory response in OA synovial tissue (171). miR-18a-3p demonstrates the ability to target and inhibit PDP1, reducing joint inflammation by downregulating IL-8, IL-6, and PGE2, as well as mitigating cartilage matrix damage through the suppression of MMP2, MMP3, and MMP9 in an OA rat model (172). Moreover, miR-224-5P alleviates synovial inflammation and OA symptoms by inhibiting PTX3, reducing P65/NF-κB activity, and promoting macrophage polarization towards an anti-inflammatory phenotype by targeting CD32 (173). Additionally, miR-212-5p, found in exosomes derived from human synovial mesenchymal stem cells, targets and inhibits ELF3, exerting both anti-inflammatory and chondroprotective effects (174). while adipose-derived stem cell exosomal miR-388-3P inhibits Runx2 expression, ameliorating IL-1β-induced cartilage damage (175).

### 3.4 MicroRNA regulation of the extracellular matrix (ECM)

Additionally, miR-485-3p targets NRP1, reducing IL-1β-induced cartilage matrix degradation by attenuating the PI3K/Akt pathway, thus slowing OA progression (176). In an *in vivo* rat model of OA induced by lipopolysaccharide (LPS) injection into the joint cavity, miR-106a was shown to alleviate OA symptoms (177). miR-21 has been linked to the activation of TLR7, exacerbating OA progression by promoting cartilage degradation (178). Finally, miR-217 suppresses SIRT1 expression, promoting inflammatory injury by elevating IL-1, IL-6, and TNF-α levels, facilitating apoptosis *via* Bax and caspase-3 upregulation, and increasing MMP-13 and MMP-9 expression through the NF-κB and P53 acetylation pathways (179). miR-217 has been found to inhibit bone anabolism by downregulating the expression of key cartilage matrix components such as COL2A1 and ACAN, ultimately accelerating the progression of OA (179). Additionally, miR-223 directly inhibits NLRP3 and reduces the expression of matrix metalloproteinases (MMPs) (180), thus protecting chondrocytes from pyroptosis and helping to stabilize the cartilage matrix in OA (181). In contrast, miR-760 has been found to target Heparin-Binding EGF-Like Growth Factor (HBEGF), promoting the degradation of the cartilage extracellular matrix and contributing to cartilage damage in OA (182). miR-322 protects the cartilage matrix from catabolic stress by targeting and inhibiting TRAF3. This inhibition leads to the upregulation of COL2A1 and ACAN, while simultaneously downregulating the expression of catabolic enzymes such as MMPs and ADAMTS5, thereby mitigating the progression of OA (183). Overexpression of miR-15a, which targets the inhibition of β1,4-GalT-I mRNA, may reduce NF-κB phosphorylation, a critical mediator of inflammation and cartilage degradation, potentially offering protection against extracellular matrix degradation and inflammatory injury in OA (184). In temporomandibular joint OA (TMJOA), miR-132-3p targets PTEN, providing protection against extracellular matrix degradation, inflammation, and chondrocyte apoptosis (185).

### 3.5 Regulation of chondrocyte autophagy by MicroRNAs (MiRs)

Furthermore, miR-128-3p, targeting ZEB1, has been shown to alleviate IL-1β-induced cartilage damage (186), while miR-128, by inhibiting NR1D2, may facilitate OA progression through the suppression of cartilage anabolism and extracellular matrix synthesis (187). miR-375 has been demonstrated to inhibit the autophagy gene ATG2B, thereby inducing endoplasmic reticulum stress and chondrocyte injury (188). It also mitigates IL-1β-induced chondrocyte apoptosis and extracellular matrix degradation by targeting PI3R3 (189). Circ_0044235 has been identified as a regulator that inhibits miR-375, influencing these pathways (154). miR-429 has also been identified as a key regulator of cartilage protection, as it targets and inhibits FEZ2, which activates autophagy, thereby safeguarding cartilage from damage and slowing OA progression (190). miR-378, by targeting Atg2a and Sox6, inhibits autophagy and impairs the chondrogenic differentiation of BMSCs, further contributing to OA pathology (191).

Collectively, these miRNAs are involved in the pathogenesis of OA by interacting with various genes and signaling pathways, exerting both positive and negative regulatory effects on inflammation, apoptosis, cartilage metabolism, and stem cell differentiation. Notably, a wide range of lncRNAs and circRNAs are actively involved in modulating the regulatory functions of miRNAs in OA, either by enhancing or counteracting their effects. The intricate crosstalk among lncRNAs, circRNAs, and miRNAs holds significant potential for advancing therapeutic strategies and preventive measures in OA. Furthermore, exosomal miRNAs play a critical role in OA’s regulatory mechanisms, emphasizing the complex intercellular communication facilitated by these exosomal miRNAs. This presents new opportunities for future treatments and prevention strategies for OA. In summary, microRNAs are pivotal regulators of key processes such as extracellular matrix homeostasis, inflammation, cellular senescence, apoptosis, and non-regulated cell death in chondrocytes during the progression of OA. Additionally, miRNAs influence the balance between proliferation and differentiation in bone marrow-derived mesenchymal stem cells (BMSCs), osteoblasts, and osteoclasts, as well as the regulation of macrophage proliferation and the phenotypic switch between pro-inflammatory (M1) and anti-inflammatory (M2) macrophages. These mechanisms highlight the critical role of miRNAs in OA pathogenesis, positioning the regulation of key miRNAs as a promising target for therapeutic interventions. Future research is expected to increasingly focus on the targeted modulation of miRNAs and the interactions between various non-coding RNAs, offering new insights and strategic directions for the effective treatment of OA.

## 4 MicroRNA (miR) influences osteoarthritis (OA) chondrocytes through the regulation of ferroptosis

**FIGURE 1:**
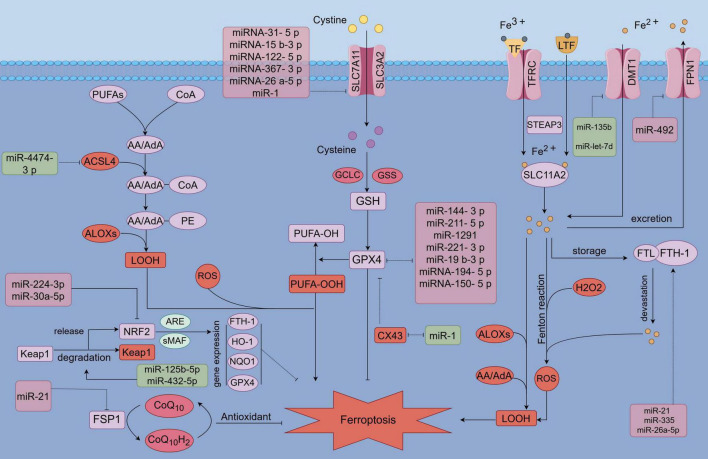
MicroRNA (MiR) involvement in the regulation of ferroptosis. MicroRNAs (miRs) modulate ferroptosis by targeting key components of antioxidant pathways, lipid metabolism, and iron metabolism pathways. Specifically, miRs influence antioxidant pathways, including GPX4, SLC7A11, NRF2, and FSP1, as well as iron metabolism-related factors such as FPN, DMT1, FTH1, and TFR1. Additionally, miRs target lipid metabolism-related factors, including ACSL4 and ALOXs.

Since its proposal in 2012, iron-dependent cell death, known as ferroptosis, has been linked to a variety of metabolic diseases (192). Ferroptosis is distinguished by the accumulation of lipid peroxides in an iron-dependent manner, ultimately causing plasma membrane rupture and cell death (193). The role of ferroptosis in chondrocytes has been extensively studied in OA, where it has been identified as a significant contributor to disease progression (8, 194). As a result, targeting chondrocyte ferroptosis has emerged as a promising therapeutic strategy for OA management. Recently, miRNAs have gained attention as potential therapeutic targets in OA, with growing evidence highlighting their regulatory roles in ferroptosis. These processes involve complex changes in the levels of downstream effectors, and various non-coding RNAs are also involved in regulating these pathways. This study explores the mechanisms through which miRNAs regulate ferroptosis and presents a comprehensive overview of miR targets and the associated pathways that modulate ferroptosis in chondrocytes, emphasizing their broader implications for OA regulation (Figure 1 and Table 1).

**TABLE 1:** Cartilage ferroptosis-associated miRNAs.

	Anabolism	Catabolism	Inflammation	Ferroptosis				References
				**Up**	**Target**	**Marker**	**Pathways**	
miR-1↑	ACAN↑ SOX9↑ OPN↓ALP↓ COL1↓BMP-2↓ OC↓COLX↓ OCN↓ COL2↑ COL2A1↑ COL10↓OPG↓	MMP-13↓ MMP-1↓ IHH↓ MMP-9↓ ADAMTS5↓	TTP↑ 1L-8↓ TNF-α↓ IL-6↓ IFNα↓ IFNβ↓ IL-1β↓ IFN-γ↑ IL-4↓ IL-5↓ IL-10↓ iNos↓		CX43↓	GPX4↑SLC7A11↑		(224, 225, 261–270)
miR-885-5p↑		MMP9↓	IL-1β↓IL-6↓TNF-α↓IL-18↑IL-6↓	LncRNA MEG3	SLC7A11↓	SLC7A11↓GPX4↓	lncRNA MEG3/miR-885-5p/SLC7A11	(231, 271–273)
miR-138-5p↑	COL2↓ SOX9↓ALP↓ COLIα1↓ ACAN↓ COL2A1↓ GAG↓	MMP-13↑ADAMTS-5↑MMP-9↓	TNF-α↓IL-6↓IL-10↑ IL-8 ↓ IL-1β↓IL-18↓Arg1 ↓	circTRIM25	CREB1↓	GPX4↑SLC7A11↑	circTRIM25/miR-138-5p/CREB1	(223, 238, 274–279)
miR-10a-5p↑	COL2↓COL2A1↑ SOX9↑ACAN ↑ BMP-2↑CRTAC1↓ALP↓	ADAMTS-5↑ MMP-13↑	IL-6↓ IL-8↓ TNF-α↓ IL-1β↓ IL-4↓ IL-17 ↓ 1L-3↑	IL-6	IL-6R↓	GPX4↑FPN1↑ DMT1↑	IL-6/miR-10a-5p/IL-6R	(230, 280–286)
miR-181b↑	COL2↑, ACAN↑ OCN↑OPN↑ ALP↑	MMP-13↓	IL-6↓ IL-8↓ TNF-α↓ iNOS↓VCAM-1↓ COX-2↓ IL-1β ↓ IL-10↑		SLC7A11↓	SLC7A11↓GPX4↓ FTH1↓TFR1↑		(232, 287–290)
miR-19b-3p↑	COL2↓COL1A1↑ ALP↑ACAN↑ sGAG↑	MMP-13↑ MMP1↓ MMP3↓ MMP9↓ ADAMTS4↓ ADAMTS5↓	IL-6↓ IL-8↓ IL-1β↓ TNF-α↓		SLC7A11↓	ACSL4↑GPX4↓ SLC7A11↓		(234, 291–293)

### 4.1 MicroRNA (miR) modulates ferroptosis by influencing factors both upstream and downstream within antioxidant-related pathways

Firstly, the SLC7A11/GSH/GPX4 pathway, recognized as the most classical antioxidant mechanism, effectively counteracts the initiation of ferroptosis through its antioxidative properties (195–197). However, microRNAs (miRNAs) play a crucial role in the regulation of ferroptosis by directly or indirectly modulating the SLC7A11/GPX4 pathway. Certain microRNAs (miRs) have been identified to directly target key components of the pathway (Figure 1). In the following discussion, we examine the indirect regulation of SLC7A11/GPX4 by several notable miRs in recent years. Specifically, miR-144-3p targets and inhibits the expression of ZEB1, which in turn suppresses the expression of GPX4 (198). Additionally, miR-211-5p targets and inhibits P2RX7, leading to a downregulation of P2RX7 that subsequently activates the MAPK/ERK pathway, thereby upregulating the expression of GPX4 (199). miR-1291 targets FOXA2, leading to the down-regulation of GSH and GPX4 expression (200). Similarly, miR-221-3p targets ATF3, resulting in the down-regulation of GPX4 expression (201). Additionally, miR-129-2-3p targets and inhibits SMAD3, thereby inhibiting GPX4 expression. Furthermore, miR-19b-3p targets and inhibits RBMS1, which subsequently up-regulates GPX4 expression (202). Conversely, circIDE acts as a sponge for miR-19b-3p, thereby exerting an opposite effect on GPX4 expression (203). MicroRNA-194-5p targets and inhibits the activity of PTGS2, leading to a downregulation of GPX4 expression (204). Similarly, microRNA-150-5p targets and suppresses c-Myb expression, which results in an increase in CDO1 expression and a subsequent decrease in GPX4 levels (205). Furthermore, microRNA-31-5p inhibits the production of BAP1 by targeting and suppressing SLC7A11 expression (206). Additionally, microRNA-15b-3p targets KLF2, thereby modulating the SLC7A11/GPX4 axis (207). MicroRNA-122-5p targets TP53, resulting in the upregulation of SLC7A11 expression (208). Similarly, microRNA-367-3p inhibits EZH2 expression, which also leads to the upregulation of SLC7A11 (209). Furthermore, microRNA-26a-5p inhibits MAT2A, thereby suppressing the SIRT1/SLC7A11 signaling pathway (210). In addition to the regulation of the SLC7A11/GPX4 pathway, microRNAs also indirectly regulate the FSP1/CoQ pathway, an antioxidant and ferroptosis-related pathway that operates independently of the GPX4 pathway (211, 212). MiR-21 targets the phosphatase and tensin homolog (PTEN), leading to the upregulation of FSP1 expression and the activation of the FSP1/CoQ10 pathway, thereby exerting antioxidant effects and mitigating ferroptosis (213). Similarly, miR-30a-5p inhibits the expression of sirtuin 1 (SIRT1) and disrupts the nuclear translocation of NRF2, resulting in the downregulation of downstream anti-ferroptotic factors such as GPX4 and FTH (214). Furthermore, miR-125b-5pand miR-432-5p promote the degradation of kelch-like ECH-associated protein 1 (Keap1), which liberates NRF2 activity and enhances the expression of antioxidant proteins that counteract ferroptosis (215, 216). Overall, microRNAs (miRs) exert regulatory effects on ferroptosis by directly targeting or indirectly modulating antioxidant pathways, including SLC7A11/GPX4, FSP1/CoQ10, and NRF2. Although these miRNAs have not been directly linked to chondrocyte ferroptosis in OA, their differential expression and involvement in oxidative stress and ferroptosis in other tissues suggest a potential regulatory role in OA. Further research is warranted to investigate the role of these differentially expressed miRNAs in chondrocyte ferroptosis and their implications for OA progression. Validating the influence of these miRNAs on chondrocyte ferroptosis could provide valuable insights into novel therapeutic targets for OA.

### 4.2 MicroRNAs (miRNAs) modulate ferroptosis by influencing pathways associated with iron and lipid metabolism

Alterations in iron metabolism are crucial for the regulation of iron-induced cell death, with certain precursor proteins involved in modulating cellular iron levels and influencing sensitivity to this form of cell death. Furthermore, microRNAs (miRs) can either directly target (Figure 1) or indirectly regulate factors associated with iron metabolism, thereby influencing the process of cellular ferroptosis. Specifically, miR-492 targets and inhibits MZF-1, resulting in a reduction of ferroportin (FPN) levels (217). This results in a decreased exclusion of intracellular iron, thereby increasing susceptibility to ferroptosis through cellular iron overload. MicroRNAs miR-135b and miR-let-7d are implicated in targeting and inhibiting DMT1 expression (29, 218), which subsequently reduces intracellular iron levels and mitigates the progression of ferroptosis. Additionally, miR-30d targets the inhibition of the autophagy-related gene ATG5, which plays a role in the regulation of ferroptosis by modulating the FTH1 autophagy process associated with iron overload-induced cell death (219). Abnormalities in lipid metabolism significantly contribute to ferroptosis, and microRNAs (MiRs) play a crucial role in the regulation of this process. Specifically, miR-4474-3p has been identified as a regulator of ferroptosis through its modulation of ALOX15 expression (220). In conclusion, MiRs are implicated in the regulation of ferroptosis by directly targeting or indirectly influencing pathways associated with the antioxidant system, lipid metabolism, and iron metabolism. While the roles of certain aforementioned microRNAs (MiRs) in osteoarthritis (OA) remain inadequately defined, several have been demonstrated to exhibit differential expression in OA chondrocytes. Consequently, further investigation into the regulatory functions of MiRs in ferroptosis within chondrocytes is both necessary and holds significant promise for the future treatment of OA.

### 4.3 Regulatory mechanisms of microRNAs associated with iron-induced chondrocyte death

miR-138-5P serves as a positive regulator of cartilage metabolism by promoting anabolic processes, reducing catabolic activity, and exerting anti-inflammatory effects in osteoarthritic (OA) cartilage (221). In OA, the expression of miR-138-5P is diminished, likely due to the inhibitory influence of the upregulated CREB1 protein. miR-138-5P directly targets and inhibits CREB1, which in turn liberates GPX4, a critical enzyme inhibited by CREB1. This pathway provides resistance to ferroptosis and oxidative stress in chondrocytes by reducing Fe2 + levels, reactive oxygen species (ROS), and malondialdehyde (MDA) (222). However, overexpression of CREB1 can counteract the protective effects of miR-138-5P. Additionally, CircTRIM25 has been identified as a direct inhibitor of miR-138-5P, promoting ferroptosis in chondrocytes. The knockdown of CircTRIM25, *via* the miR-138-5P/CREB1/GPX4 axis, presents a promising therapeutic approach for mitigating chondrocyte ferroptosis and treating OA (223). Research has shown that miR-1 is downregulated in OA (224). Under normal conditions, miR-1 serves a protective function by enhancing cartilage proliferation, regulating cartilage metabolism, and preventing chondrocyte apoptosis. It accomplishes this by upregulating key osteosynthesis-related genes such as COL2A1, ACAN, and SOX9 while suppressing osteocatabolism-related genes like MMPs and ADAMTSs. Additionally, miR-1 inhibits caspase-3 and promotes BCl-2 expression, further safeguarding chondrocytes from apoptotic damage (225). CX43, however, has been found to inhibit the proliferation of SX43 and suppress the SLC7A11/GPX4 pathway, thereby inducing ferroptosis (226). In the context of chondrocyte ferroptosis, miR-1 regulates this process by directly targeting and inhibiting CX43. This inhibition leads to a reduction in ferroptosis-related alterations in chondrocytes, such as increases in Fe2 + levels, reactive oxygen species (ROS), and malondialdehyde (MDA). By inhibiting CX43, miR-1 enhances the activity of GPX4 and SLC7A11, key players in reducing oxidative stress and ferroptosis. This mechanism plays a pivotal role in mitigating chondrocyte ferroptosis and, consequently, alleviating the progression of OA (225, 227).

Interleukin-6 (IL-6) is highly upregulated in OA and plays a significant role as a pro-inflammatory cytokine (228). IL-6 downregulates ferroportin 1 (FPN1) and upregulates divalent metal transporter 1 (DMT1), thereby increasing cellular iron uptake and reducing iron excretion (69, 229). This dysregulation results in intracellular iron overload, enhancing susceptibility to ferroptosis in chondrocytes and contributing to OA progression. The interplay between inflammation and ferroptosis in OA is further highlighted by this iron overload-induced chondrocyte death. Notably, miR-10a-5p targets and inhibits IL-6R, potentially mitigating the effects of IL-6 on chondrocyte injury and ferroptosis. This inhibition may protect cartilage by preventing IL-6-induced ferroptosis in OA (230). However, IL-6 upregulation can inhibit miR-10a-5p, enhancing IL-6R activity, which ultimately exacerbates chondrocyte ferroptosis. This mechanism may explain the observed downregulation of miR-10a-5p in osteoarthritic cartilage (230).

miR-885-5p has been identified as a direct regulator of SLC7A11, enhancing the susceptibility of C28/I2 human chondrocytes to Erastin-induced ferroptosis. The overexpression of miR-885-5p is implicated in the progression of OA by promoting ferroptosis in chondrocytes (231). Similarly, miR-181b directly targets and inhibits SLC7A11, facilitating the initiation of chondrocyte ferroptosis and advancing OA progression (232). miR-1972, miR-665, and miR-181a-2-3p have also been found to regulate the expression of GPX4 and glutathione (GSH) by modulating JUN, ATF3, and CDKN1A, which may influence chondrocyte ferroptosis (233). Furthermore, miR-19b-3P, found in synoviocyte-derived exosomes in OA, inhibits SLC7A11, exacerbating ferroptosis and oxidative stress-induced injury in chondrocytes (234). The SLC7A11/GPX4 pathway plays a critical role in protecting chondrocytes from ferroptosis by mitigating oxidative stress, and its proper function is essential for safeguarding articular cartilage from OA damage (235, 236). The role of microRNAs (miRNAs) in modulating ferroptosis has been increasingly recognized as critical to the pathogenesis of OA. Numerous miRNAs have been implicated in either promoting or resisting OA progression by targeting ferroptosis-related factors and their upstream or downstream regulators. Understanding miRNA-mediated regulation of chondrocyte ferroptosis is essential for elucidating the mechanisms underlying OA. Investigating how miRNAs influence chondrocyte iron-dependent cell death could not only improve our understanding of OA but also pave the way for novel therapeutic strategies aimed at preventing and treating the disease. This research avenue offers substantial promise for developing innovative treatments for OA in the future.

## 5 Novel avenues for the regulation of osteoarthritis-associated ferroptosis by microRNAs

It has been observed that lncRNA AC011511.5 competitively inhibits hsa-miR-520c-5p, hsa-miR-518d-5p, hsa-miR-518f-5p, and hsa-miR-665, among others, thereby regulating the expression of GABARAPL2, HMOX1, NOX4, STMN1, and TXNIP, as well as other genes implicated in OA iron-induced cell death (237). Similarly, the competitive inhibition of lncRNA AL358072.1 with hsa-miR-138-5p and hsa-miR-122-5p modulates the expression of AGPAT3, HERPUD1, JDP2, SLC38A, SQSTM1, and UBC (237). Through bioinformatics analysis, eight hub genes (ATF3, EGR1, FOSB, FOSL1, FOSL2, JUN, JUNB, and MYC) were identified as potential regulators of osteoarthritis (OA) iron-death-related genes. These hub genes modulate iron death genes, with ALOX15, CISD1, SAT1, and TFRC showing positive correlations, and ATP5MC3, GPX4, HSPB1, and MT1G exhibiting negative correlations. Consequently, these hub genes may play a role in regulating the OA iron death process (238–241). Previous studies have highlighted the significant role of these hub genes in OA (104, 242–246). This analysis suggests a potential interaction between the hub gene and ferroptosis-related genes. The recent study identified ACSF2 (247, 248), AURKA (249–252), EGFR (253, 254), and KLHL24 (51, 255) as biomarkers associated with iron deposition in osteoarthritis (OA). Additionally, a targeted investigation was conducted on EGFR (256). Epidermal growth factor receptor (EGFR) expression was significantly diminished in osteoarthritic (OA) cartilage and iron-dead chondrocytes (66), demonstrating an inhibitory effect on chondrocyte iron-induced cell death. Importantly, EGFR was competitively regulated by three long non-coding RNAs (lncRNAs: LINC00265, LINC00051, and KCTD21-AS) and four microRNAs (miRNAs: hsa-miR-6846-5p, hsa-miR-4763-3p, hsa-miR-6796-5p, and hsa-miR-6860), which collectively modulated EGFR inhibition. A recent study indicates that hsa-miR-149-3p, hsa-miR-423-5p, hsa-miR-31-5p, and hsa-miR-30b-3p may influence the progression of osteoarthritis (OA) through the regulation of CDKN1A (257, 258) and SLC39A14 (259, 260), the latter of which has been identified as a differentially expressed gene associated with ferroptosis in OA (222). In conclusion, this not only broadens the scope of scientific research but also offers a novel therapeutic direction for future exploration. The regulatory mechanisms between these genes remain poorly understood. Further investigation into the interrelationships among these genes holds significant potential for advancing our understanding of the pathogenesis of osteoarthritis and improving strategies for its prevention and management. Most importantly, this insight offers novel strategies for targeting microRNAs (MiRs) to regulate the progression of osteoarthritis (OA). Specifically, the modulation of MiRs and other non-coding RNAs to control chondrocyte ferroptosis presents a promising therapeutic approach for OA management.

## 6 Discussions

OA is a chronic inflammatory and immune-mediated disease of unknown origin, leading to irreversible joint pain, mobility loss, and significant functional impairments. These consequences greatly reduce patients’ quality of life while also imposing substantial financial burdens. Current OA management strategies focus on pain relief and partial restoration of joint function, but a complete and effective cure remains elusive. A deeper understanding of the pathogenesis of OA is essential to developing more comprehensive and innovative therapeutic approaches. In this context, microRNAs (miRNAs), small non-coding RNAs of about 20 nucleotides, have emerged as key players. miRNAs have been shown to play a pivotal role in regulating osteoarthritic chondrocytes through mechanisms such as apoptosis, autophagy, pyroptosis, and ferroptosis—types of programmed and non-programmed cell death. These regulatory pathways are intricately connected to chondrocyte anabolic and catabolic metabolism, osteoblast redox balance, the differentiation of bone marrow-derived mesenchymal stem cells into chondrocytes and osteoblasts, macrophage polarization, and other key processes in OA pathology. The regulation of miRNAs in OA occurs at the genetic level, making them promising targets for future OA treatments and preventive strategies. Numerous miRNAs have already been linked to OA progression, with additional upstream non-coding RNAs, such as lncRNAs and circRNAs, playing indirect roles by modulating miRNA activity. These upstream RNAs influence downstream pathways and further shape the regulatory network in OA. Targeting non-coding RNAs, including lncRNAs, circRNAs, and miRNAs, offers a promising avenue for the future treatment of OA. Mechanistically, the role of miRNAs in regulating chondrocyte ferroptosis, a form of cell death implicated in OA progression, deserves particular attention. Exploring this regulatory axis may enhance our understanding of OA and broaden therapeutic strategies. Despite extensive research on non-coding RNAs, including miRNAs, significant knowledge gaps remain. Current insights into their preventive, palliative, or therapeutic effects on OA are largely theoretical, necessitating rigorous clinical validation to confirm their safety and efficacy. Emerging studies on exosomal miRNAs and intra-articular injections of miRNA-related therapies have shown promise in experimental models of OA treatment and prevention. However, their clinical application faces challenges, and substantial work remains to ensure their efficacy in human patients. Bridging basic research with clinical practice through comprehensive trials and further investigation is essential for integrating these miRNA-based therapies into OA management. This research direction holds great promise for transforming the treatment landscape of OA, potentially paving the way for novel therapeutic interventions and even a future cure.
