# Supplementary material for: Regulation of ferroptosis in osteoarthritis and osteoarthritic chondrocytes by typical MicroRNAs in chondrocytes
Source: Front Med (Lausanne). 2024 Nov 5;11:1478153. doi: 10.3389/fmed.2024.1478153 (PMC11573538; doi:10.3389/fmed.2024.1478153)
Supplement: Supplementary file 1 [file Table_1.docx]

| Table1  Summary of associated mirnas and associated signaling pathways in osteoarthritis-related cells in the last 3 years. | | | | |
| --- | --- | --- | --- | --- |
|  | MiRNAs | Signaling pathways | References | |
| Osteoblast | | | | |
| Up in OA | miR-877-5p | microRNA-877-5p/EIF4G2 | | ^(1)^ |
|  | miR-1260b | miR-1260b/GDI1 | | ^(2)^ |
|  | miR-708-3p | miR-708-3p /LSD1 | | ^(3)^ |
|  | miR-181d-5p | miR-181d-5p/TNF | | ^(4)^ |
|  | miR-7-5p | miR-7-5p / LRP4 / Wnt/β-catenin | | ^(5)^ |
|  | miR-497-5p | miR-497-5p/HIF1AN | | ^(6)^ |
|  | miR-24-2-5p | lncRNAAW011738/miR-24-2-5p/TREM1 | | ^(7)^ |
|  | miR-365-2-5p | miR-365-2-5p/OLFML1 | | ^(8)^ |
|  | miR-578 | miR-578/HMGA2 | | ^(9)^ |
|  | miR-483-3p | miR-483-3p/ARRB2/MAPK | | ^(10)^ |
|  | miR-497-5p | miR-497-5p/Smurf2 | | ^(11)^ |
|  | miR-218-5p | miR-218-5p/SOCS3 | | ^(12)^ |
|  | microRNA-29b-3p | microRNA-29b-3p/sirtuin-1 | | ^(13)^ |
|  | miR-450b | ncRNA XIST/miR-450b/FBXW7 | | ^(14)^ |
|  | miR-101-3p | miR-101-3p/CDH11/SOX9 | | ^(15)^ |
|  | miR-125a-5p | miR-125a-5p/TNFAIP3/Wnt/β-catenin | | ^(16)^ |
|  | miR-513a-5p | LINC01234 miR-513a-5p/AOX1 | | ^(17)^ |
|  | miR-382 | miR-382/SLIT2 | | ^(18)^ |
|  | miR-27a-3p | miR-27a-3p/APC/Wnt/b-catenin | | ^(19)^ |
|  | miR-4739 | miR-4739/ITGA10/PI3K | | ^(20)^ |
|  | miRNA-1224-5p | miRNA-1224-5p/Rap1 /ADCY2 | | ^(21)^ |
|  | miR-708 | LncRNA TMC3-AS1/miR-708 | | ^(22)^ |
|  | miR-29cb2 | miR-29cb2/HIF-3α | | ^(23)^ |
|  | miR-215-5p | miR-215-5p/XIAP | | ^(24)^ |
|  | miR-455-3p | miR-455-3p/HDAC2 | | ^(25)^ |
|  | miR-582-3p | miR-582-3p/HOXA10 | | ^(26)^ |
|  | miR-21-5p | miR-21-5p/Dec1 | | ^(27)^ |
|  | miR-181a-5p | SNHG1/miR-181a-5p/PTEN | | ^(28)^ |
|  | miR-155-5p |  | | ^(29)^ |
|  | miR-150 | miR-150 /GREM1/NF-kappaB | | ^(30)^ |
|  | miR-138-5p | miR-138-5p/RUNX2 /Wnt/β-catenin | | ^(31)^ |
|  | miR-3129 | miR-3129/SLC7A11 | | ^(32)^ |
|  | miR-20a | miR-20a /BAMBI / SMAD6 / BMP2 | | ^(33)^ |
|  | miR-129-5p |  | | ^(34)^ |
|  | miR-466l-3p | miR-466l-3p/FGF23 | | ^(35)^ |
|  | miR-21-5p | miR-21-5p/KLF3 | | ^(36)^ |
| Down in OA | miR-124-3p | miR-124-3p/DNMT3b/TRAF6 | | ^(37)^ |
|  | miR-187-3p | miR-187-3p/CNR2 | | ^(38)^ |
|  | miR-455-3p | CircZfp644-205 / miR-455-3p /SMAD2 | | ^(39)^ |
|  | miR-483-5p | miR-483-5p/Col1A1 Mrna | | ^(40)^ |
|  | miR-338-3p | KLF3-AS1/miR-338-3p | | ^(41)^ |
|  | miR-98-5p | MicroRNA 98-5p /BMP-2 | | ^(42)^ |
|  | miR-223-3p | LncRNA MAGI2-AS3 / miR-223-3p | | ^(43)^ |
|  | miR-12200-5p | miR-12200-5p/Wnt signaling pathway | | ^(44)^ |
|  | miR-196b-5p | miR-196b-5p /SEMA3A | | ^(45)^ |
|  | miR-224-5p | miR-224-5p/ Runx2 / Sp7 | | ^(46)^ |
|  | miR-138-5p | miR-138-5p/SIRT1 | | ^(47)^ |
|  | miR-5134-5p | miR-5134-5p/JAK2/STAT3 | | ^(48)^ |
|  | miR-92a-1-5p | miR-92a-1-5p/MAPK1/FoxO1 | | ^(49)^ |
|  | miR-146a-3p | AC108925/miR-146a-3p | | ^(50)^ |
|  | miR-19a-3p | miR-19a-3p/SMAD5 | | ^(51)^ |
|  | miR-26a | miR-26a/NORAD | | ^(52)^ |
|  | miR-182-5p | miR-182-5p/Hoxa10 | | ^(53)^ |
|  | miR-212-3p | SETDB1/miR-212-3p/HMGB1 | | ^(54)^ |
|  | miR-9-5p | miR-9-5p/KLF5 | | ^(55)^ |
|  | miRNA-29b-3p | miRNA-29b-3p/MMP2 | | ^(56)^ |
|  | miR-214-3p | miR-214-3p/ATF4 | | ^(57)^ |
|  | miR-151b | miR-151b/Msx2 | | ^(58)^ |
|  | miR-30b-5p | miR-30b-5p/BCL6 | | ^(59)^ |
|  | miR-23a | OB-OC-MΦ | | ^(60)^ |
|  | miR-140-5p | IRS1/PI3K/Akt/mTOR | | ^(61)^ |
|  | miR-153-3p | miR-153-3p/CBFβ | | ^(62)^ |
|  | miR-1246 |  | | ^(63)^ |
|  | miR-146a-5p | miR-146a-5p/SMAD4 | | ^(64)^ |
|  | miR-31-5p | HIF-1α/BNIP3 | | ^(65)^ |
|  | miR-10a-5p | LncRNA /miR-10a-5p/BDNF | | ^(66)^ |
|  | miR-100-5p | miR-100-5p/TMEM135 | | ^(67)^ |
|  | miR-1270 | miR-1270/IRF8 | | ^(68)^ |
|  | miR-539-3p | miR-539-3p/Wnt/β catenin /Akap-3 | | ^(69)^ |
|  | miR-210-3p | miR-210-3p/BDNF | | ^(70)^ |
|  | miR-140-5p | miR-140-5p/VEGFA/ERK5 | | ^(71)^ |
|  | miR-103-3p | lncRNA H19/miR-103-3p/Runx2 | | ^(72)^ |
|  | miR-532-5p | circSKIL/miR-532-5p/Notch1 | | ^(73)^ |
|  | miR-197-3p | miR-197-3p/SMAD5 | | ^(74)^ |
|  | miR-150-5p | miR-150-5p/MMP14/Wnt/β-catenin | | ^(75)^ |
|  | miR-199a | miR-199a/IGF1 | | ^(76)^ |
|  | miR-96 | miR-96/Osx | | ^(77)^ |
|  | miR-92b-3p | miR-92b-3p/ELK4 | | ^(78)^ |
| Osteoclast | | | | |
| Up in OA | miR-212-3p | miR-212-3p//Smad2 | | ^(79)^ |
|  | miR-6359 | miR-6359/SIRT3/MAPK | | ^(80)^ |
|  | miR-19a | miR-19 /NF-кB/MAPK | | ^(81)^ |
|  | miR-92a-1-5p | miR-92a-1-5p/MAPK1 / FoxO1 | | ^(49)^ |
|  | miR-196b-5p | miR-196b-5p/SEMA3A | | ^(45)^ |
|  | miR-182 | miR-182/IL-8/STAT3 | | ^(82)^ |
|  | miR-31-5p | miR-31-5p/eNOS | | ^(83)^ |
|  | miR-574-5p | miR-574–5p/BMP2 | | ^(84)^ |
|  | miR-193-3p | miR-193-3p/NF-κB/NFATc1 | | ^(85)^ |
|  | miR-182 | miR-182/RANKL/NFκB | | ^(86)^ |
|  | miR-381-3p | miR-381-3p/NEK2 | | ^(87)^ |
|  | miR-28 | miR-28/RUNX1 | | ^(88)^ |
|  | miR-23a | OB-OC-MΦ | | ^(60)^ |
|  | miR-214-5p | ATF1/miR-214-5p/ITGA7 | | ^(89)^ |
|  | MiR-221-5p | MiR-221-5p/Smad3 | | ^(90)^ |
|  | miR-134-5p | miR-134-5p/Itgb1/MAPK | | ^(91)^ |
|  | miR-1260b | miR-1260b/ATF6β | | ^(92)^ |
|  | miR-214-3p |  | | ^(93)^ |
|  | miR-128-3p | miR-128-3p/NFAT5 | | ^(94)^ |
|  | miR-23b-3p | miR-23b-3p/PI3k/AKT /PTEN | | ^(95)^ |
|  | miR-107-5p | miR-107-5p/MKP1 | | ^(96)^ |
|  | miR-25 | miR-25/MCU | | ^(97)^ |
|  | miR-92a-3p | miR-92a-3p/Akt | | ^(98)^ |
|  | miR-21 |  | | ^(99)^ |
|  | mir-882 | mir-882/Rev-Erbα | | ^(100)^ |
|  | miR-96-5p | miR96-5p/Abca1 | | ^(101)^ |
|  | miR-21 | miR-21/PTEN/PI3K/Akt | | ^(102)^ |
|  | miR-655 | miR-655/IL-17 | | ^(103)^ |
| Down in OA | miR-125a-3p | miR-125a-3p/TRAF6 | | ^(104)^ |
|  | miR-483-5p | miR-483-5p/Col1A1 | | ^(40)^ |
|  | miR-134-5p | miR-134-5p/Rab27a | | ^(105)^ |
|  | miR-185 | miR-185/Btk | | ^(106)^ |
|  | miR-181 | miR-181/KAT2B/SRSF1 | | ^(107)^ |
|  | miR-15b-5p | miR-15b-5p/GFAP | | ^(108)^ |
|  | miR-130b-3p | miR-130b-3p/PTEN | | ^(109)^ |
|  | miR-3470b | miR-3470b/TAB3/NF-κB | | ^(110)^ |
|  | miR-210-3p | miR-210-3p/NF-kB1 | | ^(111)^ |
|  | miR-214 |  | | ^(112)^ |
|  | miR-155 | MicroRNA-155/SOCS1 | | ^(113)^ |
|  | miR-223-3p |  | | ^(114)^ |
|  | miR-340-5p | ZEB1/microRNA-340-5p/HMGB1 | | ^(115)^ |
|  | miR-218-2-3p | miR-218-2/RANK | | ^(116)^ |
|  | miR-124 | miR-124/NFATc1 | | ^(117)^ |
|  | miRNA-146b-5p | miRNA-146b-5p/PTEN/AKT/mTor | | ^(118)^ |
|  | miR-25-3p |  | | ^(119)^ |
|  | miR-6881-3p |  | | ^(120)^ |
|  | miR-181a-5p | miR-181a-5p/TRAF6/TAK1 | | ^(121)^ |
|  | miR-1224-5p | MiR-1224-5p/Rap1/ADCY2 | | ^(21)^ |
|  | miR-27a-3p |  | | ^(122)^ |
|  | miR-196b-5p |  | | ^(122)^ |
|  | miR-34a-5p |  | | ^(93)^ |
|  | miR-223 | IL-23/miR-223 | | ^(123)^ |
|  | miR-148a | miR-148a agomiR/AAV-shNRP1 | | ^(124)^ |
|  | miR-152 | HOTAIR/miR-152/CAMKIIα | | ^(125)^ |
| Chondrocyte | | | | |
| Up in OA | miR-204 | miR-204/SP1-LRP1 | | ^(126)^ |
|  | miR-223 | miR-223/NLRP3 | | ^(127)^ |
|  | miR-18a-3p | miR-18a-3p/PDP1 | | ^(128)^ |
|  | miR-29b-5p | miR-29b-5p/TET1 | | ^(129)^ |
|  | miR-214-3p |  | | ^(130)^ |
|  | miR-140-5p | miR-140-5p/Jagged1/Notch | | ^(131)^ |
|  | miR-124-3p | miR-124-3p/MALAT1/KLF5/ CXCL11 | | ^(132)^ |
|  | miR-125-5p | miR-125-5p/SUV39H1 | | ^(133)^ |
|  | miR-140-5p | YY1/miR-140-5p/Jagged1/Notch | | ^(134)^ |
|  | miR-92a-3p |  | | ^(135)^ |
|  | miR-140 |  | | ^(136)^ |
|  | miR-20b | miR-20b/BMP2/Smad1 | | ^(137)^ |
|  | miR-20a-5p | miR-20a-5p/Map3k2 | | ^(138)^ |
|  | miR-155 | ETS2/miR-155/STAT1/DNMT1 | | ^(139)^ |
|  | miR-455 | miR-455/SOX11/FOXO | | ^(140)^ |
|  | miR-181a-5p | miR-181a-5p/SIRT1 | | ^(141)^ |
|  | miR-148a-3p | miR-148a-3p/Wnt/β-Catenin | | ^(142)^ |
|  | miR-199a-3p |  | | ^(143)^ |
|  | miR-146a | miR-146a/TRAF6 | | ^(144)^ |
|  | miR-142-5p | miR-142-5p/RUNX2 | | ^(145)^ |
|  | miR-125 |  | | ^(146)^ |
|  | miR-485-3p | miR-485-3p/NRP1 / PI3K/Akt | | ^(147)^ |
|  | miR-455-3p | miR-455-3p/HDAC2 | | ^(148)^ |
|  | mir-99a-5p | mir-99a-5p/PI3K/AKT | | ^(149)^ |
|  | miR-204 | miR-204/SP1-LRP1 | | ^(126)^ |
| Down in OA | miR-362-5p | miR-362-5p/PLXNB1 | | ^(150)^ |
|  | miR-760 | miR-760/HBEGF | | ^(151)^ |
|  | miR-144-3p | miR-144-3p/BMP2/PI3K/Akt | | ^(152)^ |
|  | miR-146a | miR-146a/NRF2/HO-1 | | ^(153)^ |
|  | miR-199a-5p | miR-199a-5p/MAPK4 | | ^(154)^ |
|  | miR-195/497-5p |  | | ^(155)^ |
|  | miR-30a-3p | circHIPK3/miR-30a-3p/PON2 | | ^(156)^ |
|  | miR-708-5p | miR-708-5p/SATB2 | | ^(157)^ |
|  | miR-940 |  | | ^(158)^ |
|  | miR-576-5p |  | | ^(158)^ |
| Synovial macrophage | | | | |
| Up in OA | miR-135b | miR-135b/MAPK6 | | ^(159)^ |
|  | miR-492 | miR-492/SOCS2 | | ^(160)^ |
|  | miR-204/-211 |  | | ^(161)^ |
|  | miR-486-5p | circ_0066715/miR-486-5p/ETS1 | | ^(162)^ |
| Down in OA | miR-155 |  | | ^(163)^ |
|  | miR-146a | miR-146a/Toll-likereceptor 4/TRAF6/NF-κB | | ^(144)^ |
|  | miR-146b-5p | miR-146b-5p/Usp3&Sox5 | | ^(164)^ |
|  | miR-100-5p | miR-100-5p/mTOR | | ^(165)^ |
|  | miR-124-3p |  | | ^(166)^ |

**REFERENCES**

1. Shen Y, Zhang Y, Wang Q, Jiang B, Jiang X, Luo B. MicroRNA-877-5p promotes osteoblast differentiation by targeting EIF4G2 expression, *J Orthop Surg Res*. (2024) 19:134. doi: 10.1186/s13018-023-04396-y

2. Li J, Xu K, Cui Y, Xu T, Fei W, Lyu C, et al. ECM1-associated miR-1260b promotes osteogenic differentiation by targeting GDI1, *Acta Histochem*. (2024) 126:152133. doi: 10.1016/j.acthis.2024.152133

3. Shao Q, Liu S, Zou C, Ai Y. miR-708-3p targetedly regulates LSD1 to promote osteoblast differentiation of hPDLSCs in periodontitis, *Odontology*. (2024) . doi: 10.1007/s10266-024-00963-9

4. Qu F, Zhang YF, Wang YY, Cao XM, Shen YY, Wu ZA, et al. Cyclic stretch-induced exosomes from periodontal ligament cells promote osteoblasts osteogenic differentiation via the miR-181d-5p/TNF signaling pathway, *Arch Oral Biol*. (2024) 157:105843. doi: 10.1016/j.archoralbio.2023.105843

5. Liu C, Liu J, Chen H. Overexpression of miR-7-5p Promoted Fracture Healing Through Inhibiting LRP4 and Activating Wnt/β-Catenin Pathway, *Int J Low Extrem Wounds*. (2024) 23:86-91. doi: 10.1177/15347346231157443

6. Lu Y, Pan K, Zhang Y, Peng J, Cao D, Li X. The mechanism of lncRNA SNHG1 in osteogenic differentiation via miR-497-5p/ HIF1AN axis, *Connect Tissue Res*. (2024) 65:63-72. doi: 10.1080/03008207.2023.2281321

7. Liu J, Wang B, Chen H, Yu X, Cao X, Zhang H. Osteoclast-derived exosomes influence osteoblast differentiation in osteoporosis progression via the lncRNA AW011738/ miR-24-2-5p/ TREM1 axis, *Biomed Pharmacother*. (2024) 178:117231. doi: 10.1016/j.biopha.2024.117231

8. Hou C, Zhang Y, Lv Z, Luan Y, Li J, Meng C, et al. Macrophage exosomes modified by miR-365-2-5p promoted osteoblast osteogenic differentiation by targeting OLFML1, *Regen Biomater*. (2024) 11:rbae018. doi: 10.1093/rb/rbae018

9. Li F, Zhao X, Zhang Y, Zhuang Q, Wang S, Fang X, et al. Exosomal circFAM63Bsuppresses bone regeneration of postmenopausal osteoporosis via regulating miR-578/HMGA2 axis, *J Orthop Res*. (2024) 42:1244-53. doi: 10.1002/jor.25776

10. Yu X, Ge J, Xie H, Qian J, Xia W, Wang Q, et al. MiR-483-3p promotes dental pulp stem cells osteogenic differentiation via the MAPK signaling pathway by targeting ARRB2, *In Vitro Cell Dev Biol Anim*. (2024) 60:879-87. doi: 10.1007/s11626-024-00929-9

11. Hu X, Luo J. [Role and mechanism of miR-497-5p in the differentiation and mineralization of pre-osteoblast MC3T3-E1], *Shanghai Kou Qiang Yi Xue*. (2023) 32:17-22

12. Zhou Q, Zhou L, Li J. MiR-218-5p-dependent SOCS3 downregulation increases osteoblast differentiation inpostmenopausal osteoporosis, *J Orthop Surg Res*. (2023) 18:109. doi: 10.1186/s13018-023-03580-4

13. Xie H, Cao L, Ye L, Li Q, Zhang Y, Zhang H, et al. microRNA-29b-3p/sirtuin-1/peroxisome proliferator-activated receptor γ suppress osteogenic differentiation, *In Vitro Cell Dev Biol Anim*. (2023) 59:109-20. doi: 10.1007/s11626-023-00753-7

14. Song C, Guo Y, Chen F, Liu W. IRF-1-inhibited lncRNA XIST regulated the osteogenic differentiation via miR-450b/FBXW7 axis, *Apoptosis*. (2023) 28:669-80. doi: 10.1007/s10495-023-01820-w

15. Chen J, Lin Y, Sun Z. Inhibition of miR-101-3p prevents human aortic valve interstitial cell calcification through regulation of CDH11/SOX9 expression, *Mol Med*. (2023) 29:24. doi: 10.1186/s10020-023-00619-4

16. Liu C, Liang T, Zhang Z, Chen J, Xue J, Zhan X, et al. MEG3 alleviates ankylosing spondylitis by suppressing osteogenic differentiation of mesenchymal stem cells through regulating microRNA-125a-5p-mediated TNFAIP3, *Apoptosis*. (2023) 28:498-513. doi: 10.1007/s10495-022-01804-2

17. Yan Z, He Q. LINC01234 Sponging of the miR-513a-5p/AOX1 Axis is Upregulated in Osteoporosis and Regulates Osteogenic Differentiation of Bone Marrow Mesenchymal Stem Cells, *Mol Biotechnol*. (2023) 65:2108-18. doi: 10.1007/s12033-023-00712-3

18. Su H, Yang Y, Lv W, Li X, Zhao B. Bone marrow mesenchymal stem cell-derived exosomal microRNA-382 promotes osteogenesis in osteoblast via regulation of SLIT2, *J Orthop Surg Res*. (2023) 18:185. doi: 10.1186/s13018-023-03667-y

19. Arab F, Aghaee Bakhtiari SH, Pasdar A, Saburi E. Evaluation of osteogenic induction potency of miR-27a-3p in adipose tissue-derived human mesenchymal stem cells (AD-hMSCs), *Mol Biol Rep*. (2023) 50:1281-91. doi: 10.1007/s11033-022-08084-8

20. Song Y, Meng Z, Zhang S, Li N, Hu W, Li H. miR-4739/ITGA10/PI3K signaling regulates differentiation and apoptosis of osteoblast, *Regen Ther*. (2022) 21:342-50. doi: 10.1016/j.reth.2022.08.002

21. Hu L, Xie X, Xue H, Wang T, Panayi AC, Lin Z, et al. MiR-1224-5p modulates osteogenesis by coordinating osteoblast/osteoclast differentiation via the Rap1 signaling target ADCY2, *Exp Mol Med*. (2022) 54:961-72. doi: 10.1038/s12276-022-00799-9

22. Chen S, Dai M. Lipopolysaccharide-Induced lncRNA TMC3-AS1 is Highly Expressed in Osteoporosis and Promotes Osteoblast Apoptosis by Suppressing the Formation of Mature miR-708, *Int J Gen Med*. (2022) 15:3345-52. doi: 10.2147/IJGM.S350081

23. Ouyang L, Sun Y, Lv D, Peng X, Liu X, Ci L, et al. miR-29cb2 promotes angiogenesis and osteogenesis by inhibiting HIF-3α in bone, *iScience*. (2022) 25:103604. doi: 10.1016/j.isci.2021.103604

24. Yin Z, Shen J, Wang Q, Wen L, Qu W, Zhang Y. miR-215-5p regulates osteoporosis development and osteogenic differentiation by targeting XIAP, *BMC Musculoskelet Disord*. (2022) 23:789. doi: 10.1186/s12891-022-05731-w

25. Ma H, Li M, Jia Z, Chen X, Bu N. MicroRNA-455-3p promotes osteoblast differentiation via targeting HDAC2, *Injury*. (2022) 53:3636-41. doi: 10.1016/j.injury.2022.08.047

26. Yin J, Xiao W, Zhao Q, Sun J, Zhou W, Zhao W. MicroRNA-582-3p regulates osteoporosis through regulating homeobox A10 and osteoblast differentiation, *Immunopharmacol Immunotoxicol*. (2022) 44:421-8. doi: 10.1080/08923973.2022.2052895

27. Kurita T, Li X, Bhawal UK. Crosstalk between microRNA-21-5p and the transcription factor Dec1 maintains osteoblast function, *Biochem Biophys Res Commun*. (2022) 632:32-9. doi: 10.1016/j.bbrc.2022.09.090

28. Guo X, Zhang J, Han X, Wang G. LncRNA SNHG1 Delayed Fracture Healing via Modulating miR-181a-5p/PTEN Axis, *J Invest Surg*. (2022) 35:1304-12. doi: 10.1080/08941939.2022.2048926

29. Yamamura Y, Miyoshi K, Mouri Y, Kudo Y, Miyamoto Y. miR-155-5p can be involved in acquisition of osseointegration on titanium surface, *In Vitro Cell Dev Biol Anim*. (2022) 58:693-701. doi: 10.1007/s11626-022-00718-2

30. Zheng LW, Lan CN, Kong Y, Liu LH, Fan YM, Zhang CJ. Exosomal miR-150 derived from BMSCs inhibits TNF-α-mediated osteoblast apoptosis in osteonecrosis of the femoral head by GREM1/NF-κB signaling, *Regen Med*. (2022) 17:739-53. doi: 10.2217/rme-2021-0169

31. Yan F, Huo Q, Zhang W, Wu T, Dilimulati D, Shi L. MiR-138-5p targets RUNX2 to inhibit osteogenic differentiation of aortic valve interstitial cells via Wnt/β-catenin signaling pathway, *BMC Cardiovasc Disord*. (2022) 22:24. doi: 10.1186/s12872-022-02471-6

32. Nguyen AP, Yamagata K, Iwata S, Trimova G, Zhang T, Shan Y, et al. Enhancer RNA commits osteogenesis via microRNA-3129 expression in human bone marrow-derived mesenchymal stem cells, *Inflamm Regen*. (2022) 42:43. doi: 10.1186/s41232-022-00228-4

33. Peng Z, Mai Z, Xiao F, Liu G, Wang Y, Xie S, et al. MiR-20a: a mechanosensitive microRNA that regulates fluid shear stress-mediated osteogenic differentiation via the BMP2 signaling pathway by targeting BAMBI and SMAD6, *Ann Transl Med*. (2022) 10:683. doi: 10.21037/atm-22-2753

34. Deng L, Li X, Ren X, Lai S, Zhu Y, Li J, et al. A grooved porous hydroxyapatite scaffold induces osteogenic differentiation via regulation of PKA activity by upregulating miR-129-5p expression, *J Periodontal Res*. (2022) 57:1238-55. doi: 10.1111/jre.13060

35. Xiang Z, Jin X. A novel miR-466l-3p/FGF23 axis promotes osteogenic differentiation of human bone marrow mesenchymal stem cells, *BONE*. (2024) 185:117123. doi: 10.1016/j.bone.2024.117123

36. Donghao Z, Jinshan R, Jingjing L, Chunlin Y, Zhifu C, Felix Kwame A, et al. miR-21-5p Regulates the Proliferation and Differentiation of Skeletal Muscle Satellite Cells by Targeting KLF3 in Chicken, *Genes*. (2021) 12. doi: 10.3390/genes12060814

37. Zhang P, Feng Q, Chen W, Bai X. Catalpol antagonizes LPS-mediated inflammation and promotes osteoblast differentiation through the miR-124-3p/DNMT3b/TRAF6 axis, *Acta Histochem*. (2024) 126:152118. doi: 10.1016/j.acthis.2023.152118

38. Zhang L, Zeng C, Huang J, Yan H, Jiang Y, Li R. Exploration of the miR-187-3p/CNR2 pathway in modulating osteoblast differentiation and treating postmenopausal osteoporosis through mechanical stress, *FASEB J*. (2024) 38:e23776. doi: 10.1096/fj.202400113RR

39. Zhang P, Liu J, Chai Z, Fu J, Li S, Yang Z. CircZfp644-205 inhibits osteoblast differentiation and induces apoptosis of pre-osteoblasts via sponging miR-455-3p and promoting SMAD2 expression, *Eur J Med Res*. (2024) 29:315. doi: 10.1186/s40001-024-01903-7

40. Liu X, Guo L, Du J, Luo Z, Xu J, Bhawal UK, et al. Macrophage-derived apoptotic bodies impair the osteogenic ability of osteoblasts in periodontitis, *Oral Dis*. (2024) 30:3296-307. doi: 10.1111/odi.14808

41. Liu D, Zhao X, Zhang Q, Zhou F, Tong X. Bone marrow mesenchymal stem cell-derived exosomes promote osteoblast proliferation, migration and inhibit apoptosis by regulating KLF3-AS1/miR-338-3p, *BMC Musculoskelet Disord*. (2024) 25:122. doi: 10.1186/s12891-024-07236-0

42. Zhang YB, Guo XQ, Wang GG, Pu HB. MicroRNA 98-5p Overexpression Contributes to Delayed Fracture Healing via Targeting BMP-2, *Tohoku J Exp Med*. (2024) 263:17-25. doi: 10.1620/tjem.2024.J005

43. Dong Z, Hu B, Wang S, Wang M, Sun S, Liu X, et al. LncRNA MAGI2-AS3 promotes fracture healing through downregulation of miR-223-3p, *J Orthop Surg Res*. (2024) 19:370. doi: 10.1186/s13018-024-04850-5

44. Li H, Yin C, Li J, Huang Q, Huai Y, Chu X, et al. MiR-12200-5p Targets Multiple Members of Wnt Signaling Pathway to Inhibit Osteoblast Differentiation and Bone Formation, *Endocr Metab Immune Disord Drug Targets*. (2023) 23:1254-64. doi: 10.2174/1871530323666230301150350

45. Xie Y, Zhou J, Tian L, Dong Y, Yuan H, Zhu E, et al. miR-196b-5p Regulates Osteoblast and Osteoclast Differentiation and Bone Homeostasis by Targeting SEMA3A, *J Bone Miner Res*. (2023) 38:1175-91. doi: 10.1002/jbmr.4834

46. Ding S, Ma Y, Yang J, Tang Y, Jin Y, Li L, et al. MiR-224-5p inhibits osteoblast differentiation and impairs bone formation by targeting Runx2 and Sp7, *Cytotechnology*. (2023) 75:505-16. doi: 10.1007/s10616-023-00593-z

47. Xu L, Zhang X, Li G, Zhang L, Zhang S, Shi F, et al. Inhibition of SIRT1 by miR-138-5p provides a mechanism for inhibiting osteoblast proliferation and promoting apoptosis under simulated microgravity, *Life Sci Space Res (Amst)*. (2023) 36:59-69. doi: 10.1016/j.lssr.2022.08.001

48. Pan L, Zhang C, Zhang H, Ke T, Bian M, Yang Y, et al. Osteoclast-Derived Exosomal miR-5134-5p Interferes with Alveolar Bone Homeostasis by Targeting the JAK2/STAT3 Axis, *Int J Nanomedicine*. (2023) 18:3727-44. doi: 10.2147/IJN.S413692

49. Yu L, Sui B, Zhang X, Liu J, Hao X, Zheng L. miR-92a-1-5p enriched prostate cancer extracellular vesicles regulate osteoclast function via MAPK1 and FoxO1, *J Exp Clin Cancer Res*. (2023) 42:109. doi: 10.1186/s13046-023-02685-2

50. Liu J, Zhao Z, Deng Z, Chen X, Li W. LncRNA AC108925 promotes osteoblast differentiation of tendon-derived stem cells by targeting miR-146a-3p, *Pathol Res Pract*. (2023) 241:154230. doi: 10.1016/j.prp.2022.154230

51. Lin H, Nie L, Lu G, Wu H, Xu T. Long non-coding RNA KCNQ10T1/miR-19a-3p/SMAD5 axis promotes osteogenic differentiation of mouse bone mesenchymal stem cells, *J Orthop Surg Res*. (2023) 18:929. doi: 10.1186/s13018-023-04425-w

52. Chen S, Ma H, Li M, Jia Z, Chen X, Bu N. Long Noncoding RNA NORAD Promotes Fracture Healing through Interacting with Osteoblast Differentiation via Targeting miR-26a, *Biomed Res Int*. (2023) 2023:9950037. doi: 10.1155/2023/9950037

53. Huang Y, Tao M, Yan S, He X. Long non-coding RNA Homeobox D gene cluster antisense growth-associated long noncoding RNA/microRNA-182-5p/Homeobox protein A10 alleviates postmenopausal osteoporosis via accelerating osteoblast differentiation of bone marrow mesenchymal stem cells, *J Orthop Surg Res*. (2023) 18:726. doi: 10.1186/s13018-023-04203-8

54. Zhang L, Xu L, Wang Y, Zhang X, Xue T, Sun Q, et al. Histone methyltransferase Setdb1 mediates osteogenic differentiation by suppressing the expression of miR-212-3p under mechanical unloading, *Cell Signal*. (2023) 102:110554. doi: 10.1016/j.cellsig.2022.110554

55. Li B, Ding T, Chen H, Li C, Chen B, Xu X, et al. CircStrn3 targeting microRNA-9-5p is involved in the regulation of cartilage degeneration and subchondral bone remodelling in osteoarthritis, *Bone Joint Res*. (2023) 12:33-45. doi: 10.1302/2046-3758.121.BJR-2022-0231.R1

56. Zhong-Sheng Z, Nuo Y, Xiao-Yan H, Liang D, Rui F, Hai-Jun X. MMP2 promotes osteoblast differentiation and calcification of muscle-derived mesenchymal stem cells by interaction with miR-29b-3p, *Tissue Cell*. (2022) 76:101807. doi: 10.1016/j.tice.2022.101807

57. Zhang K, Liu X, Tang Y, Liu Z, Yi Q, Wang L, et al. Fluid Shear Stress Promotes Osteoblast Proliferation and Suppresses Mitochondrial-Mediated Osteoblast Apoptosis Through the miR-214-3p-ATF4 Signaling Axis, *Physiol Res*. (2022) 71:527-38. doi: 10.33549/physiolres.934917

58. Liu F, Liang Y, Lin X. MiR-151b inhibits osteoblast differentiation via downregulating Msx2, *Connect Tissue Res*. (2022) 63:112-23. doi: 10.1080/03008207.2021.1882442

59. Luo Y, Zhou F, Wu X, Li Y, Ye B. miR-30b-5p inhibits osteoblast differentiation through targeting BCL6, *Cell Cycle*. (2022) 21:630-40. doi: 10.1080/15384101.2022.2031428

60. Ma TL, Zhu P, Ke ZR, Chen JX, Hu YH, Xie J. Focusing on OB-OC-MΦ Axis and miR-23a to Explore the Pathogenesis and Treatment Strategy of Osteoporosis, *Front Endocrinol (Lausanne)*. (2022) 13:891313. doi: 10.3389/fendo.2022.891313

61. Tang Y, Sun Y, Zeng J, Yuan B, Zhao Y, Geng X, et al. Exosomal miR-140-5p inhibits osteogenesis by targeting IGF1R and regulating the mTOR pathway in ossification of the posterior longitudinal ligament, *J Nanobiotechnology*. (2022) 20:452. doi: 10.1186/s12951-022-01655-8

62. Wei C, Chu M, Zheng K, He P, Xiao J. miR-153-3p inhibited osteogenic differentiation of human DPSCs through CBFβ signaling, *In Vitro Cell Dev Biol Anim*. (2022) 58:316-24. doi: 10.1007/s11626-022-00665-y

63. Jia E, Zhu H, Geng H, Zhong L, Qiu X, Xie J, et al. The Inhibition of Osteoblast Viability by Monosodium Urate Crystal-Stimulated Neutrophil-Derived Exosomes, *Front Immunol*. (2022) 13:809586. doi: 10.3389/fimmu.2022.809586

64. Liu X, Zhang K, Wang L, Geng B, Liu Z, Yi Q, et al. Fluid shear stress-induced down-regulation of miR-146a-5p inhibits osteoblast apoptosis via targeting SMAD4, *Physiol Res*. (2022) 71:835-48. doi: 10.33549/physiolres.934922

65. Fu HH, Sun LG, Ding CC, Ma XR, Huang YM. [Effects of miR-31-5p on HIF-1α/BNIP3 signaling pathway and the expression of osteoblast-related factors of dental pulp stem cells], *Shanghai Kou Qiang Yi Xue*. (2022) 31:237-42

66. Li X, Huang Y, Han Y, Yang Q, Zheng Y, Li W. LncPVT1 regulates osteogenic differentiation of human periodontal ligament cells via miR-10a-5p/brain-derived neurotrophic factor, *J Periodontol*. (2022) 93:1093-106. doi: 10.1002/JPER.21-0429

67. Wang R, Zhang M, Hu Y, He J, Lin Q, Peng N. MiR-100-5p inhibits osteogenic differentiation of human bone mesenchymal stromal cells by targeting TMEM135, *Hum Cell*. (2022) 35:1671-83. doi: 10.1007/s13577-022-00764-8

68. Ramírez-Salazar EG, Almeraya EV, López-Perez TV, Jiménez-Salas Z, Patiño N, Velázquez-Cruz R. MicroRNA-1270 Inhibits Cell Proliferation, Migration, and Invasion via Targeting IRF8 in Osteoblast-like Cell Lines, *Curr Issues Mol Biol*. (2022) 44:1182-90. doi: 10.3390/cimb44030077

69. Tripathi A, John AA, Kumar D, Kaushal SK, Singh DP, Husain N, et al. MiR-539-3p impairs osteogenesis by suppressing Wnt interaction with LRP-6 co-receptor and subsequent inhibition of Akap-3 signaling pathway, *Front Endocrinol (Lausanne)*. (2022) 13:977347. doi: 10.3389/fendo.2022.977347

70. Deng L, Lai S, Fan L, Li X, Huang H, Mu Y. miR-210-3p suppresses osteogenic differentiation of MC3T3-E1 by targeting brain derived neurotrophic factor (BDNF), *J Orthop Surg Res*. (2022) 17:418. doi: 10.1186/s13018-022-03315-x

71. Wang X, Geng B, Wang H, Wang S, Zhao D, He J, et al. Fluid shear stress-induced down-regulation of microRNA-140-5p promotes osteoblast proliferation by targeting VEGFA via the ERK5 pathway, *Connect Tissue Res*. (2022) 63:156-68. doi: 10.1080/03008207.2021.1891228

72. Zhou W, Feng Q, Cheng M, Zhang D, Jin J, Zhang S, et al. LncRNA H19 sponges miR-103-3p to promote the high phosphorus-induced osteoblast phenotypic transition of vascular smooth muscle cells by upregulating Runx2, *Cell Signal*. (2022) 91:110220. doi: 10.1016/j.cellsig.2021.110220

73. Zhong X, Wang H. circSKIL promotes osteoblastic differentiation of periodontal ligament cells by sponging miR-532-5p to activate Notch signaling, *J Periodontal Res*. (2022) 57:1148-58. doi: 10.1111/jre.13052

74. Chen Q, Shen P, Zhang B, Chen Y, Zheng C. Circ_0062582 promotes osteogenic differentiation of human bone marrow mesenchymal stem cells in vitro by elevating SMAD5 expression through sponging miR-197-3p, *Cells Tissues Organs*. (2022) . doi: 10.1159/000525703

75. Xu C, Wang Z, Liu Y, Wei B, Liu X, Duan K, et al. Extracellular vesicles derived from bone marrow mesenchymal stem cells loaded on magnetic nanoparticles delay the progression of diabetic osteoporosis via delivery of miR-150-5p, *Cell Biol Toxicol*. (2023) 39:1257-74. doi: 10.1007/s10565-022-09744-y

76. Lin WL, Wu XP, Wang XM, He WW. [Effect of miR-199a regulating IGF1 expression on differentiation of osteoblasts under mechanical stimulation], *Shanghai Kou Qiang Yi Xue*. (2022) 31:132-7

77. Zang LY, Yang XL, Li WJ, Liu GL. Long Noncoding RNA Metastasis-Associated Lung Adenocarcinoma Transcript 1 Promotes the Osteoblast Differentiation of Human Bone Marrow-Derived Mesenchymal Stem Cells by Targeting the microRNA-96/Osterix Axis, *J Craniofac Surg*. (2022) 33:956-61. doi: 10.1097/SCS.0000000000008092

78. Zhang X, Zhang L, Xu L, Li G, Wang K, Xue T, et al. Exosomes from Microvascular Endothelial Cells under Mechanical Unloading Inhibit Osteogenic Differentiation via miR-92b-3p/ELK4 Axis, *J Pers Med*. (2022) 12:2030. doi: 10.3390/jpm12122030

79. Dai J, Hu Z, Zeng F, Gong X, Tang H, Deng J, et al. Osteoclast-derived exosomal miR-212-3p suppressed the anabolism and accelerated the catabolism of chondrocytes in osteoarthritis by targeting TGF-β1/Smad2 signaling, *Arch Biochem Biophys*. (2024) 751:109827. doi: 10.1016/j.abb.2023.109827

80. Xie X, Cheng P, Hu L, Zhou W, Zhang D, Knoedler S, et al. Bone-targeting engineered small extracellular vesicles carrying anti-miR-6359-CGGGAGC prevent valproic acid-induced bone loss, *Signal Transduct Target Ther*. (2024) 9:24. doi: 10.1038/s41392-023-01726-8

81. Lei Z, Wang Q, Jiang Q, Liu H, Xu L, Kang H, et al. The miR-19a/Cylindromatosis Axis Regulates Pituitary Adenoma Bone Invasion by Promoting Osteoclast Differentiation, *Cancers (Basel)*. (2024) 16:302. doi: 10.3390/cancers16020302

82. Zhao MN, Zhang LF, Sun Z, Qiao LH, Yang T, Ren YZ, et al. A novel microRNA-182/Interleukin-8 regulatory axis controls osteolytic bone metastasis of lung cancer, *Cell Death Dis*. (2023) 14:298. doi: 10.1038/s41419-023-05819-8

83. Lu J, Yu N, Liu Q, Xie Y, Zhen L. Periodontal Ligament Stem Cell Exosomes Key to Regulate Periodontal Regeneration by miR-31-5p in Mice Model, *Int J Nanomedicine*. (2023) 18:5327-42. doi: 10.2147/IJN.S409664

84. Li T, Li M, Peng Y, Li X, Chen Y, Zhang J, et al. Liver cancer cell-secreted exosomes promote bone metastasis of liver cancer by facilitating osteoclast differentiation through the miR-574-5p/BMP2 axis, *Pathol Res Pract*. (2023) 245:154485. doi: 10.1016/j.prp.2023.154485

85. Liu X, Xu X, Li J, Shi L, Zeng Y, Tang S, et al. Isobavachalcone inhibits RANKL-induced osteoclastogenesis via miR-193-3p/NF-κB/NFATc1 signaling pathway in BMMs cells, *Biosci Biotechnol Biochem*. (2023) 87:960-71. doi: 10.1093/bbb/zbad075

86. Zhao B, Luo M, Wang A, Zhang W. MiR-182 antagonist alleviates glucocorticoid-induced secondary bone degeneration and osteoclast differentiation, *Cell Mol Biol (Noisy-le-grand)*. (2022) 67:123-30. doi: 10.14715/cmb/2021.67.5.17

87. Wang Y, Zhu G, Pei F, Zhao Z. lncRNA SNHG15 as a ceRNA modulates Osteoclast Differentiation, Proliferation, and Metastasis by Sponging miR-381-3p/NEK2 Axis, *J Immunol Res*. (2022) 2022:8634820. doi: 10.1155/2022/8634820

88. Zhang X, Zhao Z, Chen Y, Han X, Jie Y. Extracellular vesicles secreted by human periodontal ligament induced osteoclast differentiation by transporting miR-28 to osteoclast precursor cells and further promoted orthodontic tooth movement, *Int Immunopharmacol*. (2022) 113:109388. doi: 10.1016/j.intimp.2022.109388

89. Liu LL, Xiao YS, Huang WM, Liu S, Huang LX, Zhong JH, et al. ATF1/miR-214-5p/ITGA7 axis promotes osteoclastogenesis to alter OVX-induced bone absorption, *Mol Med*. (2022) 28:56. doi: 10.1186/s10020-022-00476-7

90. Guo M, Liu N, Guo Z. MiR-221-5p/Smad3 axis in osteoclastogenesis and its function: Potential therapeutic target for osteoporosis, *Steroids*. (2022) 185:109063. doi: 10.1016/j.steroids.2022.109063

91. Huang M, Wang Y, Wang Z, Qin Q, Zhang H, Liu S, et al. miR-134-5p inhibits osteoclastogenesis through a novel miR-134-5p/Itgb1/MAPK pathway, *J Biol Chem*. (2022) 298:102116. doi: 10.1016/j.jbc.2022.102116

92. Hayashi C, Fukuda T, Kawakami K, Toyoda M, Nakao Y, Watanabe Y, et al. miR-1260b inhibits periodontal bone loss by targeting ATF6β mediated regulation of ER stress, *Front Cell Dev Biol*. (2022) 10:1061216. doi: 10.3389/fcell.2022.1061216

93. John AA, Xie J, Yang YS, Kim JM, Lin C, Ma H, et al. AAV-mediated delivery of osteoblast/osteoclast-regulating miRNAs for osteoporosis therapy, *Mol Ther Nucleic Acids*. (2022) 29:296-311. doi: 10.1016/j.omtn.2022.07.008

94. Zhang H, Chen L, Wang Z, Sun Z, Shan Y, Li Q, et al. Long noncoding RNA KCNQ1OT1 inhibits osteoclast differentiation by regulating the miR-128-3p/NFAT5 axis, *Aging (Albany NY)*. (2022) 14:4486-99. doi: 10.18632/aging.204088

95. Chai J, Xu L, Liu N. miR-23b-3p regulates differentiation of osteoclasts by targeting PTEN via the PI3k/AKT pathway, *Arch Med Sci*. (2022) 18:1542-57. doi: 10.5114/aoms.2019.87520

96. Li G, Sul OJ, Yu R, Choi HS. 7-Ketocholesterol-Induced Micro-RNA-107-5p Increases Number and Activity of Osteoclasts by Targeting MKP1, *Int J Mol Sci*. (2022) 23:3697. doi: 10.3390/ijms23073697

97. Hu W, Yu Y, Sun Y, Yuan F, Zhao F. MiR-25 overexpression inhibits titanium particle-induced osteoclast differentiation via down-regulation of mitochondrial calcium uniporter in vitro, *J Orthop Surg Res*. (2022) 17:133. doi: 10.1186/s13018-022-03030-7

98. Uehara N, Kyumoto-Nakamura Y, Mikami Y, Hayatsu M, Sonoda S, Yamaza T, et al. miR-92a-3p encapsulated in bone metastatic mammary tumor cell-derived extracellular vesicles modulates mature osteoclast longevity, *Cancer Sci*. (2022) 113:4219-29. doi: 10.1111/cas.15557

99. Hu CH, Sui BD, Liu J, Dang L, Chen J, Zheng CX, et al. Sympathetic Neurostress Drives Osteoblastic Exosomal MiR-21 Transfer to Disrupt Bone Homeostasis and Promote Osteopenia, *Small Methods*. (2022) 6:e2100763. doi: 10.1002/smtd.202100763

100. Tian Y, Ming J. Melatonin inhibits osteoclastogenesis via RANKL/OPG suppression mediated by Rev-Erbα in osteoblasts, *J Cell Mol Med*. (2022) 26:4032-47. doi: 10.1111/jcmm.17440

101. Wang T, Mo L, Ou J, Fang Q, Wu H, Wu Y, et al. Proteus mirabilis Vesicles Induce Mitochondrial Apoptosis by Regulating miR96-5p/Abca1 to Inhibit Osteoclastogenesis and Bone Loss, *Front Immunol*. (2022) 13:833040. doi: 10.3389/fimmu.2022.833040

102. Fang B, Zhang K, Zhang J, Chen Z, Xuan Y, Huang H. Mechanical strain regulates osteoclastogenesis via modulating the PTEN/PI3K/Akt signal pathway through miR-21, *Cytotechnology*. (2022) 74:65-75. doi: 10.1007/s10616-021-00507-x

103. Liu SC, Hsieh HL, Tsai CH, Fong YC, Ko CY, Wu HC, et al. CCN2 Facilitates IL-17 Production and Osteoclastogenesis in Human Osteoarthritis Synovial Fibroblasts by Inhibiting miR-655 Expression, *J Bone Miner Res*. (2022) 37:1944-55. doi: 10.1002/jbmr.4661

104. Zhang J, Zhang L, Yao G, Zhao H, Qiao P, Wu S. lncRNA-Gm5532 regulates osteoclast differentiation through the miR-125a-3p/TRAF6 axis, *Acta Biochim Biophys Sin (Shanghai)*. (2024) 56:54-61. doi: 10.3724/abbs.2023245

105. Tang L, Yuan L, Yan J, Ge J, Lian Z, Li Z. circ_0029463 promotes osteoclast differentiation by mediating miR-134-5p/Rab27a axis, *J Orthop Surg Res*. (2024) 19:128. doi: 10.1186/s13018-024-04610-5

106. He D, Jiao Y, Xu J, Luo J, Cui Y, Han X, et al. mmu-miR-185 regulates osteoclasts differentiation and migration by targeting Btk, *J Gene Med*. (2024) 26:e3687. doi: 10.1002/jgm.3687

107. Wang C, Chen R, Zhu X, Zhang X, Lian N. DOT1L decelerates the development of osteoporosis by inhibiting SRSF1 transcriptional activity via microRNA-181-mediated KAT2B inhibition, *Genomics*. (2024) 116:110759. doi: 10.1016/j.ygeno.2023.110759

108. Xu C, Wang Z, Liu Y, Duan K, Guan J. Delivery of miR-15b-5p via magnetic nanoparticle-enhanced bone marrow mesenchymal stem cell-derived extracellular vesicles mitigates diabetic osteoporosis by targeting GFAP, *Cell Biol Toxicol*. (2024) 40:52. doi: 10.1007/s10565-024-09877-2

109. Lin W, Wu X, He W, Wang X, Gao Y, Dong W. LncRNA XIST regulates osteoclast formation and promotes orthodontically induced inflammatory root resorption through miR-130b-3p/PTEN axis, *Biotechnol Genet Eng Rev*. (2023) :1-17. doi: 10.1080/02648725.2023.2200331

110. Pan B, Zhang Z, Wu X, Xian G, Hu X, Gu M, et al. Macrophage-derived exosomes modulate wear particle-induced osteolysis via miR-3470b targeting TAB3/NF-κB signaling, *Bioact Mater*. (2023) 26:181-93. doi: 10.1016/j.bioactmat.2023.02.028

111. Tian J, Chen W, Xiong Y, Li Q, Kong S, Li M, et al. Small extracellular vesicles derived from hypoxic preconditioned dental pulp stem cells ameliorate inflammatory osteolysis by modulating macrophage polarization and osteoclastogenesis, *Bioact Mater*. (2023) 22:326-42. doi: 10.1016/j.bioactmat.2022.10.001

112. Xu L, Xu X, Liang Y, Wen C, Ouyang K, Huang J, et al. Osteoclast-targeted delivery of anti-miRNA oligonucleotides by red blood cell extracellular vesicles, *J Control Release*. (2023) 358:259-72. doi: 10.1016/j.jconrel.2023.04.043

113. Jiao Y, Mi S, Li X, Liu Y, Han N, Xu J, et al. MicroRNA-155 targets SOCS1 to inhibit osteoclast differentiation during orthodontic tooth movement, *BMC Oral Health*. (2023) 23:955. doi: 10.1186/s12903-023-03443-8

114. Li X, Jiang Y, Liu X, Fu J, Du J, Luo Z, et al. Mesenchymal stem cell-derived apoptotic bodies alleviate alveolar bone destruction by regulating osteoclast differentiation and function, *Int J Oral Sci*. (2023) 15:51. doi: 10.1038/s41368-023-00255-y

115. Chen H, Zheng Q, Lv Y, Yang Z, Fu Q. CUL4A-mediated ZEB1/microRNA-340-5p/HMGB1 axis promotes the development of osteoporosis, *J Biochem Mol Toxicol*. (2023) 37:e23373. doi: 10.1002/jbt.23373

116. Kim EY, Kim JE, Chung SH, Park JE, Yoon D, Min HJ, et al. Concomitant induction of SLIT3 and microRNA-218-2 in macrophages by toll-like receptor 4 activation limits osteoclast commitment, *Cell Commun Signal*. (2023) 21:213. doi: 10.1186/s12964-023-01226-w

117. Zhang C, Pan L, Zhang H, Ke T, Yang Y, Zhang L, et al. Osteoblasts-Derived Exosomal lncRNA-MALAT1 Promotes Osteoclastogenesis by Targeting the miR-124/NFATc1 Signaling Axis in Bone Marrow-Derived Macrophages, *Int J Nanomedicine*. (2023) 18:781-95. doi: 10.2147/IJN.S395607

118. Yin S, Lin S, Xu J, Yang G, Chen H, Jiang X. Dominoes with interlocking consequences triggered by zinc: involvement of microelement-stimulated MSC-derived exosomes in senile osteogenesis and osteoclast dialogue, *J Nanobiotechnology*. (2023) 21:346. doi: 10.1186/s12951-023-02085-w

119. Jia T, Yuan F, Tao J, Wang G, Zhang X, Zhang B, et al. CRISPR/Cas13d targeting GZMA in PARs pathway regulates the function of osteoclasts in chronic apical periodontitis, *Cell Mol Biol Lett*. (2023) 28:70. doi: 10.1186/s11658-023-00477-2

120. Liu Z, Zhang N, Xin B, Shi Y, Liang Z, Wan Y, et al. Exosomes from LSD1 knockdown breast cancer cells activate osteoclastogenesis and inhibit osteoblastogenesis, *Int J Biol Macromol*. (2023) 235:123792. doi: 10.1016/j.ijbiomac.2023.123792

121. Xue HY, Liu MW, Yang G. Resveratrol suppresses lipopolysaccharide-mediated activation of osteoclast precursor RAW 264.7 cells by increasing miR-181a-5p expression, *Int J Immunopathol Pharmacol*. (2023) 37:3946320231154995. doi: 10.1177/03946320231154995

122. Lai G, Zhao R, Zhuang W, Hou Z, Yang Z, He P, et al. BMSC-derived exosomal miR-27a-3p and miR-196b-5p regulate bone remodeling in ovariectomized rats, *PeerJ*. (2022) 10:e13744. doi: 10.7717/peerj.13744

123. Chen SY, Tsai TC, Li YT, Ding YC, Wang CT, Hsieh JL, et al. Interleukin-23 Mediates Osteoclastogenesis in Collagen-Induced Arthritis by Modulating MicroRNA-223, *Int J Mol Sci*. (2022) 23:9718. doi: 10.3390/ijms23179718

124. Pan B, Zheng L, Liu S, Fang J, Lou C, Hu X, et al. MiR-148a deletion protects from bone loss in physiological and estrogen-deficient mice by targeting NRP1, *Cell Death Discov*. (2022) 8:470. doi: 10.1038/s41420-022-01261-5

125. Dong C, Liu X, Li J, Lan D, Zheng S. Dysregulation of the HOTAIR-miR-152-CAMKIIα Axis in Craniosynostosis Results in Impaired Osteoclast Differentiation, *Front Genet*. (2022) 13:787734. doi: 10.3389/fgene.2022.787734

126. Lu K, Wang Q, Hao L, Wei G, Wang T, Lu WW, et al. miR-204 ameliorates osteoarthritis pain by inhibiting SP1-LRP1 signaling and blocking neuro-cartilage interaction, *Bioact Mater*. (2023) 26:425-36. doi: 10.1016/j.bioactmat.2023.03.010

127. Liu W, Liu A, Li X, Sun Z, Sun Z, Liu Y, et al. Dual-engineered cartilage-targeting extracellular vesicles derived from mesenchymal stem cells enhance osteoarthritis treatment via miR-223/NLRP3/pyroptosis axis: Toward a precision therapy, *Bioact Mater*. (2023) 30:169-83. doi: 10.1016/j.bioactmat.2023.06.012

128. Feng X, Lu J, Wu Y, Xu H. MiR-18a-3p improves cartilage matrix remodeling and inhibits inflammation in osteoarthritis by suppressing PDP1, *J Physiol Sci*. (2022) 72:3. doi: 10.1186/s12576-022-00827-3

129. Zhu J, Yang S, Qi Y, Gong Z, Zhang H, Liang K, et al. Stem cell-homing hydrogel-based miR-29b-5p delivery promotes cartilage regeneration by suppressing senescence in an osteoarthritis rat model, *Sci Adv*. (2022) 8:eabk0011. doi: 10.1126/sciadv.abk0011

130. Lai C, Liao B, Peng S, Fang P, Bao N, Zhang L. Synovial fibroblast-miR-214-3p-derived exosomes inhibit inflammation and degeneration of cartilage tissues of osteoarthritis rats, *Mol Cell Biochem*. (2023) 478:637-49. doi: 10.1007/s11010-022-04535-9

131. Chen Y, Huang H, Zhong W, Li L, Lu Y, Si HB. miR-140-5p protects cartilage progenitor/stem cells from fate changes in knee osteoarthritis, *Int Immunopharmacol*. (2023) 114:109576. doi: 10.1016/j.intimp.2022.109576

132. Rozi R, Zhou Y, Rong K, Chen P. miR-124-3p sabotages lncRNA MALAT1 stability to repress chondrocyte pyroptosis and relieve cartilage injury in osteoarthritis, *J Orthop Surg Res*. (2022) 17:453. doi: 10.1186/s13018-022-03334-8

133. Chen D, Jiang X. Exosomes-derived miR-125-5p from cartilage endplate stem cells regulates autophagy and ECM metabolism in nucleus pulposus by targeting SUV38H1, *Exp Cell Res*. (2022) 414:113066. doi: 10.1016/j.yexcr.2022.113066

134. Chen Y, Liao G, Ma T, Li L, Yang J, Shen B, et al. YY1/miR-140-5p/Jagged1/Notch axis mediates cartilage progenitor/stem cells fate reprogramming in knee osteoarthritis, *Int Immunopharmacol*. (2023) 121:110438. doi: 10.1016/j.intimp.2023.110438

135. Tie K, Zhao Z, Wu Z, Qin J, Zhang J, Pei L, et al. Low miR-92a-3p in oocytes mediates the multigenerational and transgenerational inheritance of poor cartilage quality in rat induced by prenatal dexamethasone exposure, *Biochem Pharmacol*. (2022) 203:115196. doi: 10.1016/j.bcp.2022.115196

136. Shi Y, Shao X, Sun M, Ma J, Li B, Zou N, et al. MiR-140 is involved in T-2 toxin-induced matrix degradation of articular cartilage, *Toxicon*. (2023) 222:106987. doi: 10.1016/j.toxicon.2022.106987

137. Zheng Z, Wang D, Zhang W, Yu H, Zhang Y, Feng T, et al. MiR-20 promotes cartilage repair in knee osteoarthritis rats via BMP2/Smad1 signaling pathway, *Cell Mol Biol (Noisy-le-grand)*. (2023) 69:256-61. doi: 10.14715/cmb/2023.69.12.40

138. Liu J, Tang G, Liu W, Zhou Y, Fan C, Zhang W. MiR-20a-5p facilitates cartilage repair in osteoarthritis via suppressing mitogen-activated protein kinase kinase kinase 2, *Bioengineered*. (2022) 13:13801-14. doi: 10.1080/21655979.2022.2084270

139. Chen S, Zhu X, Ou W, Kang L, Situ J, Liao Z, et al. ETS2 overexpression ameliorates cartilage injury in osteoarthritis by the ETS2/miR-155/STAT1/DNMT1 feedback loop pathway, *Biochim Biophys Acta Gene Regul Mech*. (2023) 1866:194965. doi: 10.1016/j.bbagrm.2023.194965

140. Sun Y, Zhao J, Wu Q, Zhang Y, You Y, Jiang W, et al. Chondrogenic primed extracellular vesicles activate miR-455/SOX11/FOXO axis for cartilage regeneration and osteoarthritis treatment, *NPJ Regen Med*. (2022) 7:53. doi: 10.1038/s41536-022-00250-7

141. Qi H, Zhao Z, Xu L, Zhang Y, Li Y, Xiao L, et al. Antisense Oligonucleotide-Based Therapy on miR-181a-5p Alleviates Cartilage Degradation of Temporomandibular Joint Osteoarthritis via Promoting SIRT1, *Front Pharmacol*. (2022) 13:898334. doi: 10.3389/fphar.2022.898334

142. Cheng L, Huang C, Li M, Shang S, Chen J, Tang Z. Chonggu Granules Improve Cartilage Matrix Metabolism in Knee Osteoarthritis via the miR-148a-3p/Wnt/β-Catenin Pathway, *J Inflamm Res*. (2023) 16:4751-62. doi: 10.2147/JIR.S428582

143. Zhao S, Xiu G, Wang J, Wen Y, Lu J, Wu B, et al. Engineering exosomes derived from subcutaneous fat MSCs specially promote cartilage repair as miR-199a-3p delivery vehicles in Osteoarthritis, *J Nanobiotechnology*. (2023) 21:341. doi: 10.1186/s12951-023-02086-9

144. Wang H, Zhang Y, Zhang C, Zhao Y, Shu J, Tang X. Exosomes derived from miR-146a-overexpressing fibroblast-like synoviocytes in cartilage degradation and macrophage M1 polarization: a novel protective agent for osteoarthritis, *Front Immunol*. (2024) 15:1361606. doi: 10.3389/fimmu.2024.1361606

145. Zeng G, Deng G, Xiao S, Li F. Fibroblast-like Synoviocytes-derived Exosomal PCGEM1 Accelerates IL-1β-induced Apoptosis and Cartilage Matrix Degradation by miR-142-5p/RUNX2 in Chondrocytes, *Immunol Invest*. (2022) 51:1284-301. doi: 10.1080/08820139.2021.1936010

146. Guan Z, Liu Y, Luo L, Jin X, Guan Z, Yang J, et al. Sympathetic innervation induces exosomal miR-125 transfer from osteoarthritic chondrocytes, disrupting subchondral bone homeostasis and aggravating cartilage damage in aging mice, *J Adv Res*. (2024) :S2090-1232(24)00122-00122X [pii]. doi: 10.1016/j.jare.2024.03.022

147. Qiu M, Xie Y, Tan G, Wang X, Huang P, Hong L. Synovial mesenchymal stem cell-derived exosomal miR-485-3p relieves cartilage damage in osteoarthritis by targeting the NRP1-mediated PI3K/Akt pathway: Exosomal miR-485-3p relieves cartilage damage, *Heliyon*. (2024) 10:e24042. doi: 10.1016/j.heliyon.2024.e24042

148. Liu L, Yu F, Chen L, Xia L, Wu C, Fang B. Lithium-Containing Biomaterials Stimulate Cartilage Repair through Bone Marrow Stromal Cells-Derived Exosomal miR-455-3p and Histone H3 Acetylation, *Adv Healthc Mater*. (2023) 12:e2202390. doi: 10.1002/adhm.202202390

149. Zhang J, Zheng K, Wu Y, Zhang S, Guo A, Sui C. The experimental study of mir-99a-5p negative regulation of TLR8 receptor mediated-mediated innate immune response in rabbit knee cartilage injury, *Immun Inflamm Dis*. (2024) 12:e1211. doi: 10.1002/iid3.1211

150. Li Q, Xu P, Zhang C, Gao Y. MiR-362-5p inhibits cartilage repair in osteoarthritis via targeting plexin B1, *J Orthop Surg (Hong Kong)*. (2022) 30:10225536221139887. doi: 10.1177/10225536221139887

151. Zhu Y, Zhang C, Jiang B, Dong Q. MiR-760 targets HBEGF to control cartilage extracellular matrix degradation in osteoarthritis, *J Orthop Surg Res*. (2023) 18:186. doi: 10.1186/s13018-023-03664-1

152. Mo ML, Jiang JM, Long XP, Xie LH. miR-144-3p aggravated cartilage injury in rheumatoid arthritis by regulating BMP2/PI3K/Akt axis, *Mod Rheumatol*. (2022) 32:1064-76. doi: 10.1093/mr/roab105

153. Zhou X, Zhang Y, Hou M, Liu H, Yang H, Chen X, et al. Melatonin Prevents Cartilage Degradation in Early-Stage Osteoarthritis Through Activation of miR-146a/NRF2/HO-1 Axis, *J Bone Miner Res*. (2022) 37:1056-72. doi: 10.1002/jbmr.4527

154. Lu H, Yang Y, Ou S, Qi Y, Li G, He H, et al. The silencing of miR-199a-5p protects the articular cartilage through MAPK4 in osteoarthritis, *Ann Transl Med*. (2022) 10:601. doi: 10.21037/atm-22-2057

155. Shi S, Zhang L, Wang Q, Wang Q, Li D, Sun W, et al. Targeting Cartilage miR-195/497 Cluster for Osteoarthritis Treatment Regulates the Circadian Clock, *Gerontology*. (2024) 70:59-75. doi: 10.1159/000534292

156. Shang J, Li H, Wu B, Jiang N, Wang B, Wang D, et al. CircHIPK3 prevents chondrocyte apoptosis and cartilage degradation by sponging miR-30a-3p and promoting PON2, *Cell Prolif*. (2022) 55:e13285. doi: 10.1111/cpr.13285

157. Wang Y, Su Q, Tang H, Tian Q, Lin X, Fu M, et al. Microfracture technique combined with mesenchymal stem cells inducer represses miR-708-5p to target special at-rich sequence-binding protein 2 to drive cartilage repair and regeneration in rabbit knee osteoarthritis, *Growth Factors*. (2023) 41:115-29. doi: 10.1080/08977194.2023.2227269

158. Zhan JW, Wang SQ, Chen M, Sun K, Yu J, Li LH, et al. [Salvianolic acid A contributes to cartilage endplate cell restoration by regulating miR-940 and miR-576-5p], *Zhongguo Gu Shang*. (2023) 36:982-9. doi: 10.12200/j.issn.1003-0034.2023.10.014

159. Wang R, Xu B. TGF-β1-modified MSC-derived exosomal miR-135b attenuates cartilage injury via promoting M2 synovial macrophage polarization by targeting MAPK6, *Cell Tissue Res*. (2021) 384:113-27. doi: 10.1007/s00441-020-03319-1

160. Zhang J, Cheng F, Rong G, Tang Z, Gui B. Circular RNA hsa_circ_0005567 overexpression promotes M2 type macrophage polarization through miR-492/SOCS2 axis to inhibit osteoarthritis progression, *Bioengineered*. (2021) 12:8920-30. doi: 10.1080/21655979.2021.1989999

161. Teng H, Chen S, Fan K, Wang Q, Xu B, Chen D, et al. Dexamethasone Liposomes Alleviate Osteoarthritis in miR-204/-211-Deficient Mice by Repolarizing Synovial Macrophages to M2 Phenotypes, *Mol Pharm*. (2023) 20:3843-53. doi: 10.1021/acs.molpharmaceut.2c00979

162. Wan L, Liu J, Huang C, Zhu Z, Li F, Sun G, et al. Role of m6A modification and novel circ_0066715/ miR-486-5p/ ETS1 axis in rheumatoid arthritis macrophage polarization progression, *Aging (Albany NY)*. (2022) 14:10009-26. doi: 10.18632/aging.204439

163. Wang H, Zhang H, Fan K, Zhang D, Hu A, Zeng X, et al. Frugoside delays osteoarthritis progression via inhibiting miR-155-modulated synovial macrophage M1 polarization, *Rheumatology (Oxford)*. (2021) 60:4899-909. doi: 10.1093/rheumatology/keab018

164. Jia H, Duan L, Yu P, Zhou Y, Liu R, Wang H. Digoxin ameliorates joint inflammatory microenvironment by downregulating synovial macrophage M1-like-polarization and its-derived exosomal miR-146b-5p/Usp3&Sox5 axis, *Int Immunopharmacol*. (2022) 111:109135. doi: 10.1016/j.intimp.2022.109135

165. Liu H, Chen Y, Huang Y, Wei L, Ran J, Li Q, et al. Macrophage-derived mir-100-5p orchestrates synovial proliferation and inflammation in rheumatoid arthritis through mTOR signaling, *J Nanobiotechnology*. (2024) 22:197. doi: 10.1186/s12951-024-02444-1

166. Nakamachi Y, Uto K, Hayashi S, Okano T, Morinobu A, Kuroda R, et al. Exosomes derived from synovial fibroblasts from patients with rheumatoid arthritis promote macrophage migration that can be suppressed by miR-124-3p, *Heliyon*. (2023) 9:e14986. doi: 10.1016/j.heliyon.2023.e14986
