# Supplementary material for: Regulation of ferroptosis in osteoarthritis and osteoarthritic chondrocytes by typical MicroRNAs in chondrocytes
Source: Front Med (Lausanne). 2024 Nov 5;11:1478153. doi: 10.3389/fmed.2024.1478153 (PMC11573538; doi:10.3389/fmed.2024.1478153)
Supplement: Supplementary file 2 [file Table_2.docx]

Table2 Mirnas of various modes of chondrocyte death

|  | Up | | Down | |
| --- | --- | --- | --- | --- |
| apoptosis | miR-203a-3p^(1)^ | miR-1183^(2)^ | miR-99a^(3)^ | miR-19b-3p^(4)^ |
|  | miR-146a-5p^(5)^ | miR-224-5p^(6)^ | miR-543^(7)^ | miR-130a-3p^(8)^ |
|  | miR-590-5p^(9)^ | miR-1236^(10)^ | miR-107^(11)^ | miR-369-3p^(12)^ |
|  | miR-455-3p^(13)^ | miR-495^(14)^ | miR-142-5p^(15)^ | miR-1179^(16)^ |
|  | miR-34a^(17)^ | miR-122-5p^(18)^ | miR-337-3p^(19)^ | miR-671-3p^(20)^ |
|  | miR-155^(21)^ | miR-195-5p^(22)^ | miR-203a-5p^(23)^ | miR-193a-3p^(24)^ |
|  | miR-361-5p^(25)^ | miR-382-5p^(26)^ | miR-152^(27)^ | miR-373^(28)^ |
|  | miR-525-5p^(29)^ | miR-150^(30)^ | miR-214-3p^(27)^ | miR-149-5p^(31)^ |
|  | miR-1307-5p^(32)^ | miR-375^(33)^ | miR-30b-5p^(34)^ | miR-93^(35)^ |
|  | miR-30b-5p^(34)^ | miR-181a-5p^(36)^ | miR-140-5p^(37)^ | miR-200b^(38)^ |
|  | miR-539-3p^(39)^ | miR-125b-5p^(40)^ | miR-539-3p^(39)^ | miR-125b^(41)^ |
|  | miR-10a-5p^(42)^ | miR-20a^(43)^ | miR-200b-3p^(44)^ | miR-502-5p^(45)^ |
|  | miR-139^(46)^ | miR-29b-3p^(47)^ | miR-330-5p^(48)^ | miR-224-5p^(49)^ |
|  | miR-30b^(50)^ | miR-23b-3p^(51)^ | miR-33b-3p^(52)^ | miR-27b-3p^(53)^ |
|  | miR-106a-5p^(54)^ | miR-125b^(55)^ | miR-206^(56)^ | miR-613^(57)^ |
|  | miR-377-3p^(58)^ | miR-29b^(59)^ | miR-128-3p^(60)^ | miR-497^(61)^ |
|  | miR-145^(62)^ | miR-27b-3p^(63)^ | miR-216a-5p^(64)^ | miR-24-3p^(65)^ |
|  | miR-146a^(66)^ | miR-1323^(67)^ | miR-152-3p^(68)^ | miR-643^(69)^ |
|  | miR-30a-3p^(70)^ | miR-155^(21)^ | miR-373-3p^(71)^ | miR-16-5p^(72)^ |
|  | miR-29b-3p^(47)^ | miR-195^(73)^ | miR-103a-3p^(74)^ | miR-671-5p^(75)^ |
|  | miR-7-5p^(76)^ | miR-9-5p^(77)^ | miR-515-5p^(78)^ | miR-149^(79)^ |
|  | miR-495^(80)^ | miR-138-5p^(81)^ | miR-520c-3p^(82)^ | miR-125a-5p^(83)^ |
|  | miR-3619-5p^(84)^ | miR-199a-3p^(85)^ | miR-296-5p^(86)^ | miR-127-5p^(87)^ |
|  | miR-599^(88)^ | miR-21-5p^(89)^ | miR-142-3p^(90)^ | miR-296-3p^(91)^ |
|  | miR-3189-3p^(92)^ | miR-29b^(59)^ | miR-93-5p^(93)^ | miR-663a^(94)^ |
|  | miR-665^(95)^ | miR-101a-3p^(96)^ | miR-485-3p^(97)^ | miR-222^(98)^ |
|  | miR-26a^(99)^ | miR-206a-3p^(100)^ | miR-940^(101)^ | miR-322-5p^(102)^ |
|  | miR-7^(103)^ | miR-324-3p^(104)^ | miR-675-3p^(105)^ | miR-210^(106)^ |
|  | miR-98^(107)^ | miR-16^(108)^ | miR-17-5p^(109)^ | miR-26b-5p^(110)^ |
|  | miR-23b^(111)^ | miR-137^(112)^ | miR-421^(113)^ | miR-558^(114)^ |
|  | miR-29a-3p^(115)^ | miR-34a-5p^(116)^ | miR-320^(117)^ | miR-199-3p^(118)^ |
|  | miR-15b^(119)^ | miR-448^(120)^ | miR-183-5p^(121)^ | miR-221-3p^(122)^ |
|  | miR-204^(123)^ | miR-101^(124)^ | miR-211^(125)^ | miR-1207-5p^(126)^ |
|  | miR-374b-5p^(127)^ | miR-376-3p^(128)^ | miR-100-5p^(129)^ | miR-26a-5p^(130)^ |
|  | miR-144-3p^(131)^ | miR-106b^(132)^ | miR-146a-3p^(133)^ | miR-9^(134)^ |
|  | miR-182-5p^(135)^ | miR-653-5p^(136)^ | miR-140-3p^(137)^ | miR-591^(138)^ |
|  | miR-18a-3p^(139)^ | miR-211-3p^(140)^ | miR-548d-5p^(141)^ | miR-1277^(142)^ |
|  | miR-206^(143)^ | miR-93^(35)^ | miR-766-3p^(144)^ | miR-107^(145)^ |
|  | miR-29a^(146)^ | miR-384-5p^(147)^ | miR-671^(148)^ | miR-4428^(149)^ |
|  | MiR-30b-5p^(150)^ | miR-30a-5p^(151)^ | miR-27-3p^(152)^ | miR-3960^(153)^ |
|  | miR-330-5p^(154)^ | miR-141^(155)^ | miR-223-3p^(156)^ | miR-451^(157)^ |
|  | miR-146b^(158)^ | miR-26a^(159)^ | miR-320a^(160)^ | miR-373^(161)^ |
|  | miR-199a-5p^(162)^ | miR-423-5p^(163)^ | miR-503-5p^(164)^ | miR-221^(165)^ |
|  | miR-335-5P^(166)^ | miR-141-3p^(167)^ | miR-222-3p^(168)^ | miR-98-5p^(169)^ |
|  | miR-186-5p^(170)^ | miR-124-3p^(171)^ | miR-25-3p^(172)^ | miR-199-3p^(173)^ |
|  | miR-1271^(174)^ | miR-363-3p^(175)^ | miR-137^(112)^ | miR-410-3p^(176)^ |
|  | miR-21-5p^(177)^ | miR-21^(178)^ | miR-27a^(179)^ | miR-138^(180)^ |
|  | miR-218-5p^(181)^ | miR-1271-5P^(182)^ | miR-3622b-5p^(183)^ | miR-20b^(184)^ |
|  | miR-146a-5p^(185)^ | miR-3680-3p^(186)^ | miR-214^(187)^ | miR-556-5p^(188)^ |
|  | miR-302d-3p^(189)^ | miR-223^(190)^ | miR-93-5p^(191)^ | miR-23b^(192)^ |
|  |  |  | miR-193a-3p^(193)^ | miR-665^(194)^ |
|  |  |  | miR-155^(21)^ | miR-301a^(195)^ |
|  |  |  | miR-4303^(196)^ | miR-19a^(197)^ |
|  |  |  | miR-107-5p^(198)^ | miR-9-5p^(77)^ |
|  |  |  | miR-3473b^(199)^ | miR-135a^(200)^ |
|  |  |  | miR-486-5p^(201)^ | miR-378^(202)^ |
|  |  |  | miR-129-3p^(203)^ | miR-577^(204)^ |
|  |  |  | miR-197-3p^(205)^ | miR-124^(206)^ |
|  |  |  | miR-361-5p^(207)^ | miR-105-5p^(208)^ |
|  |  |  | miR-145^(209)^ | miR-877-5p^(210)^ |
|  |  |  | miR-497-5p^(211)^ | miR-532-5p^(212)^ |
|  |  |  | miR-34a-5p^(213)^ | miR-140^(214)^ |
|  |  |  | miR-485-3p^(215)^ | miR-18a^(216)^ |
|  |  |  | miR-337^(33)^ | miR-15a-5p^(217)^ |
|  |  |  | miR-375^(218)^ | miR-132-3p^(219)^ |
|  |  |  | miR-558^(220)^ | miR-219a-5p^(221)^ |
|  |  |  | miR-1^(222)^ | miR-328-3p^(223)^ |
| pyroptosis | miR-155^(224)^ |  | MiR-203a-3p^(1)^ | miR-219a-5p^(221)^ |
|  | miR-144-3p^(225)^ |  | miR-140-5p^(226)^ | miR-107^(227)^ |
|  | miR-9-5p^(228)^ |  | miR-223^(229)^ | miR-25-3p^(230)^ |
|  |  |  | miR-124-3p^(171)^ | miR-558^(220)^ |
|  |  |  | miR-326^(231)^ | miR-21-5p^(89)^ |
| autophagy | miR-100-5p^(232)^ | miR-153-3p^(233)^ | miR-146a-5p^(5)^ | miR-146a^(234)^ |
|  | miR-27a^(235)^ | miR-140-3p^(236)^ | miR-20^(237)^ | miR-375^(218)^ |
|  | miR-31-5p^(238)^ | miR-421-3p^(239)^ | miR-495^(80)^ | miR-378^(240)^ |
|  | miR-142-5p^(241)^ | miR-130a-3p^(242)^ | miR-667-5p^(243)^ | miR-128a^(244)^ |
|  | mir-199a-3p^(85)^ | miR-766-3p^(144)^ | miR-128-3p^(245)^ | miR-93-5p^(191)^ |
|  | miR-140-5p^(246)^ | miR-411^(247)^ | miR-30b^(50)^ | miR-1912-3p^(248)^ |
|  | miR-149^(246)^ | miR-199a-5p^(162)^ | miR-34a^(249)^ | miR-497-5p^(211)^ |
|  | miR-335-5p^(250)^ | miR-373^(161)^ | miR-128a^(251)^ | miR-122-5p^(252)^ |
|  | miR-125-5p^(253)^ | miR-149-5p^(254)^ | miR-142-3p^(90)^ | miR-199-3p^(255)^ |
|  | miR-7^(256)^ | miR-429^(257)^ | miR-224-5p^(258)^ | miR-34a-5p^(259)^ |
|  | miR-146b-5p^(260)^ | miR-342-5p^(261)^ | miR-7-5p^(76)^ | miR-449a-5p^(262)^ |
|  | miR-21^(89)^ | miR-145^(263)^ | miR-155^(264)^ | miR-206^(265)^ |
|  | miR-126-5p^(266)^ | miR-29a-3p^(267)^ | miR-17-5p^(268)^ | miR-1271^(269)^ |
|  |  |  |  | miR-206^(270)^ |
| ferroptosis | miR-885-5p^(271)^ |  | miR-1^(272)^ |  |
|  | miR-181b^(273)^ |  | miR-138-5p^(274)^ |  |
|  | miR-19b-3p^(275)^ |  | miR-10a-5p^(276)^ |  |

**REFERENCES**

1. Jiayi C, Zhutong L, He S, Mange L, Jiangliang W, Chenxiao Z, et al. MiR-203a-3p attenuates apoptosis and pyroptosis of chondrocytes by regulating the MYD88/NF-κB pathway to alleviate osteoarthritis progression, *Aging*. (2023) 15:14457-72. doi: 10.18632/aging.205373

2. Xiaowei L, Yali S, Jimei T. CircCDK14 ameliorates interleukin-1β-induced chondrocyte damage by the miR-1183/KLF5 pathway in osteoarthritis, *AUTOIMMUNITY*. (2022) 55:408-17. doi: 10.1080/08916934.2022.2081843

3. Yeyang W, Xiaoyu Z, Dixin L, Wangyang X, Xiaozhong Z. MiR-99a alleviates apoptosis and extracellular matrix degradation in experimentally induced spine osteoarthritis by targeting FZD8, *BMC MUSCULOSKELETAL DISORDERS*. (2022) 23:872. doi: 10.1186/s12891-022-05822-8

4. Liang S, Liang D, Dapeng D, Yayi F, Honghai X. MiR-19b-3p Attenuates Chondrocytes Injury by Inhibiting MAPK/NF-Κb Axis via Targeting SOCS1, *Evid Based Complement Alternat Med*. (2022) 2022:5133754. doi: 10.1155/2022/5133754

5. Hongjun Z, Wendi Z, Du L, Jia Z. miR-146a-5p Promotes Chondrocyte Apoptosis and Inhibits Autophagy of Osteoarthritis by Targeting NUMB, *Cartilage*. (2021) 13:1467S-1477S. doi: 10.1177/19476035211023550

6. Hao L, Yong C, Chongfei C, Wenping H, Songchuan S, Zhenggang P, et al. Knockdown of circSOD2 ameliorates osteoarthritis progression via the miR-224-5p/PRDX3 axis, *Journal of Orthopaedic Surgery and Research*. (2023) 18:432. doi: 10.1186/s13018-023-03880-9

7. Peng X, Xu Z, Jinpeng S, Yuhang Z, Weijian Q, Jianqiang L, et al. LncRNA NEAT1 regulates chondrocyte proliferation and apoptosis via targeting miR-543/PLA2G4A axis, *Human Cell*. (2021) 34:60-75. doi: 10.1007/s13577-020-00433-8

8. Yunzhou Z, Changjun X, Xuewen G, Wei X, Xiaokang Y, Yanzhao C, et al. LncRNA HAGLR silencing inhibits IL-1β-induced chondrocytes inflammatory injury via miR-130a-3p/JAK1 axis, *Journal of Orthopaedic Surgery and Research*. (2023) 18:203. doi: 10.1186/s13018-023-03661-4

9. Jiang P, Dou X, Li S, Jia Q, Ling P, Liu H, et al. miR-590-5p affects chondrocyte proliferation, apoptosis, and inflammation by targeting FGF18 in osteoarthritis, *Am J Transl Res*. (2021) 13:8728-41

10. Wan-Tao W, Zhi-Peng H, Shi S, Jian-Hui L, Da-Miao Y, Wen-Bo W. microRNA-1236 promotes chondrocyte apoptosis in osteoarthritis via direct suppression of PIK3R3, *LIFE SCIENCES*. (2020) 253:117694. doi: 10.1016/j.lfs.2020.117694

11. Feng T, Junhu W, Zhanhua Z, Jie Y. miR-107 modulates chondrocyte proliferation, apoptosis, and extracellular matrix synthesis by targeting PTEN, *International Journal of Clinical and Experimental Pathology*. (2019) 12:488-97

12. Zhenyu T, Zongming G, Xiaoliang S. Long non-coding RNA musculin antisense RNA 1 promotes proliferation and suppresses apoptosis in osteoarthritic chondrocytes via the microRNA-369-3p/Janus kinase-2/ signal transducers and activators of transcription 3 axis, *Bioengineered*. (2022) 13:1554-64. doi: 10.1080/21655979.2021.2013028

13. Wen X, Li H, Sun H, Zeng A, Lin R, Zhao J, et al. MiR-455-3p reduces apoptosis and alleviates degeneration of chondrocyte through regulating PI3K/AKT pathway, *Life Sci*. (2020) 253:117718. doi: 10.1016/j.lfs.2020.117718

14. Xingyu Z, Tiejun W, Bo C, Xiaoning W, Wei F, Yu H, et al. MicroRNA-495 enhances chondrocyte apoptosis, senescence and promotes the progression of osteoarthritis by targeting AKT1, *American Journal of Translational Research*. (2019) 11:2232-44

15. Lin S, Huiling Z, Jianmin S, Xiang G, Chunhong L. CircSEC24A promotes IL-1β-induced apoptosis and inflammation in chondrocytes by regulating miR-142-5p/SOX5 axis, *BIOTECHNOLOGY AND APPLIED BIOCHEMISTRY*. (2022) 69:701-13. doi: 10.1002/bab.2145

16. Zhiping L, Peng L, Yangyang T, Hongchang T, Lianxiang L. Hsa_circ_0007292 promotes chondrocyte injury in osteoarthritis via targeting the miR-1179/HMGB1 axis, *Journal of Orthopaedic Surgery and Research*. (2023) 18:544. doi: 10.1186/s13018-023-04026-7

17. Chen H, Wang J, Hu B, Wu X, Chen Y, Li R, et al. MiR-34a promotes Fas-mediated cartilage endplate chondrocyte apoptosis by targeting Bcl-2, *Mol Cell Biochem*. (2015) 406:21-30. doi: 10.1007/s11010-015-2420-4

18. Xiang L, Ya Z, Xuefeng C, Hongjun W, Shuang Y, Jun Y, et al. Semi-synthetic chondroitin sulfate CS-semi5 upregulates miR-122-5p, conferring a therapeutic effect on osteoarthritis <i>via</i> the p38/MMP13 pathway, *Acta Pharmaceutica Sinica B*. (2024) 14:3528-42. doi: 10.1016/j.apsb.2024.05.016

19. Zhihui H, Wenming M, Jinhuai X, Xiaoyu D, Weiqi L. CircRNA_0092516 regulates chondrocyte proliferation and apoptosis in osteoarthritis through the miR-337-3p/PTEN axis, *J Biochem*. (2021) 169:467-75. doi: 10.1093/jb/mvaa119

20. Zhengjie L, Shunguang C, Yezi Y, Shengjun L, Xunming Z, Biao H, et al. MicroRNA‑671‑3p regulates the development of knee osteoarthritis by targeting TRAF3 in chondrocytes, *Molecular Medicine Reports*. (2019) 20:2843-50. doi: 10.3892/mmr.2019.10488

21. Fan Z, Liu Y, Shi Z, Deng K, Zhang H, Li Q, et al. MiR-155 promotes interleukin-1β-induced chondrocyte apoptosis and catabolic activity by targeting PIK3R1-mediated PI3K/Akt pathway, *J Cell Mol Med*. (2020) 24:8441-51. doi: 10.1111/jcmm.15388

22. Yang S, Junpeng L, Weixiong G, Wei Y. MicroRNA‑195‑5p inhibitor prevents the development of osteoarthritis by targeting REGγ, *Molecular Medicine Reports*. (2019) 19:4561-8. doi: 10.3892/mmr.2019.10124

23. Zhao W, Hongwei B, Jingzhao H, Bin J, Yong J. Circ-NFKB1 sponges miR-203a-5p to regulate ERBB4 expression and promotes IL-1β induced chondrocytes apoptosis, *Journal of Orthopaedic Surgery and Research*. (2023) 18:528. doi: 10.1186/s13018-023-03990-4

24. Xiao O, Yunzhi D, Li Y, Feng X, Xiaowei Y, Xingyong L, et al. Circular RNA CircDHRS3 Aggravates IL-1β-induced ECM Degradation, Apoptosis, and Inflammatory Response via Mediating MECP2 Expression, *INFLAMMATION*. (2023) 46:1670-83. doi: 10.1007/s10753-023-01832-3

25. Wang A, Hu N, Zhang Y, Chen Y, Su C, Lv Y, et al. MEG3 promotes proliferation and inhibits apoptosis in osteoarthritis chondrocytes by miR-361-5p/FOXO1 axis, *BMC Med Genomics*. (2019) 12:201. doi: 10.1186/s12920-019-0649-6

26. Nie X, Liu H, Wei X, Li L, Lan L, Fan L, et al. miRNA-382-5p Suppresses the Expression of Farnesoid X Receptor to Promote Progression of Liver Cancer, *Cancer Manag Res*. (2021) 13:8025-35. doi: 10.2147/CMAR.S324072

27. Wan D, Qu Y, Ai S, Cheng L. miR-152 Attenuates Apoptosis in Chondrocytes and Degeneration of Cartilages in Osteoarthritis Rats via TCF-4 Pathway, *Dose Response*. (2020) 18:1559325820946918. doi: 10.1177/1559325820946918

28. Guang C, Tao L, Bofan Y, Bingyi W, Qiang P. CircRNA-UBE2G1 regulates LPS-induced osteoarthritis through miR-373/HIF-1a axis, *Cell cycle (Georgetown, Tex.)*. (2020) 19:1696-705. doi: 10.1080/15384101.2020.1772545

29. He W, Lin X. LINC00313 promotes the proliferation and inhibits the apoptosis of chondrocytes via regulating miR-525-5p/GDF5 axis, *J Orthop Surg Res*. (2023) 18:137. doi: 10.1186/s13018-023-03610-1

30. Meng J, Kai X, Huafeng R, Mingmin W, Ximin H, Jianping C. Role of lincRNA-Cox2 targeting miR-150 in regulating the viability of chondrocytes in osteoarthritis, *Exp Ther Med*. (2021) 22:800. doi: 10.3892/etm.2021.10232

31. Shengqiang F, Qiaoyun F, Juntao X, Shengjun Y, Ming S, Yuan J, et al. Circ_0008956 contributes to IL-1β-induced osteoarthritis progression via miR-149-5p/NAMPT axis, *International Immunopharmacology*. (2021) 98:107857. doi: 10.1016/j.intimp.2021.107857

32. Qiu Z, Ma X, Xie J, Liu Z, Zhang Y, Xia C. miR-1307-5p regulates proliferation and apoptosis of chondrocytes in osteoarthritis by specifically inhibiting transforming growth factor beta-induced gene, *Am J Transl Res*. (2021) 13:7756-66

33. Xuegang S, Lidong M, Guangyu D, Chuanxiu S, Shengwei H. Platelet-rich plasma treatment alleviates osteoarthritis-related pain, inflammation, and apoptosis by upregulating the expression levels of microRNA-375 and microRNA-337, *IMMUNOPHARMACOLOGY AND IMMUNOTOXICOLOGY*. (2022) 44:87-98. doi: 10.1080/08923973.2021.2007263

34. Lishan L, Yaling Y, Kangping L, Yixin J, Zhenlei Z. Downregulation of miR-30b-5p Facilitates Chondrocyte Hypertrophy and Apoptosis via Targeting Runx2 in Steroid-Induced Osteonecrosis of the Femoral Head, *INTERNATIONAL JOURNAL OF MOLECULAR SCIENCES*. (2022) 23. doi: 10.3390/ijms231911275

35. Yanjie D, Laifang W, Qing Z, Zhenzhen W, Lingli K. MicroRNA‑93 inhibits chondrocyte apoptosis and inflammation in osteoarthritis by targeting the TLR4/NF‑κB signaling pathway, *INTERNATIONAL JOURNAL OF MOLECULAR MEDICINE*. (2019) 43:779-90. doi: 10.3892/ijmm.2018.4033

36. Suolin Z, Min T. The lncRNA MIAT/miR-181a-5p axis regulates osteopontin (OPN)-mediated proliferation and apoptosis of human chondrocytes in osteoarthritis, *JOURNAL OF MOLECULAR HISTOLOGY*. (2022) 53:285-96. doi: 10.1007/s10735-022-10067-9

37. Bo Y, Lei X, Shuang W. Regulation of lncRNA-H19/miR-140-5p in cartilage matrix degradation and calcification in osteoarthritis, *Ann Palliat Med*. (2020) 9:1896-904. doi: 10.21037/apm-20-929

38. Yintai L, Suizhuan W, Zhongping Z. MicroRNA-200b relieves LPS-induced inflammatory injury by targeting FUT4 in knee articular chondrocytes <i>in vitro</i>, *Exp Ther Med*. (2021) 21:407. doi: 10.3892/etm.2021.9838

39. T J, H Z, X F, T W, K Y, Y H. MiR-539-3p Alleviates Apoptosis and Extracellular Matrix Degradation in Chondrocytes of Childhood-Onset Osteoarthritis by Targeting RUNX2, *PHYSIOLOGICAL RESEARCH*. (2024) 73:415-26. doi: 10.33549/physiolres.935291

40. Feng-Xiao G, Haitao L, Xin Y. Upregulation of microRNA-125b-5p is involved in the pathogenesis of osteoarthritis by downregulating SYVN1, *ONCOLOGY REPORTS*. (2017) 37:2490-6. doi: 10.3892/or.2017.5475

41. Jia J, Wang J, Zhang J, Cui M, Sun X, Li Q, et al. MiR-125b Inhibits LPS-Induced Inflammatory Injury via Targeting MIP-1α in Chondrogenic Cell ATDC5, *Cell Physiol Biochem*. (2018) 45:2305-16. doi: 10.1159/000488178

42. Jiang H, Pang H, Wu P, Cao Z, Li Z, Yang X. LncRNA SNHG5 promotes chondrocyte proliferation and inhibits apoptosis in osteoarthritis by regulating miR-10a-5p/H3F3B axis, *Connect Tissue Res*. (2021) 62:605-14. doi: 10.1080/03008207.2020.1825701

43. Zhao H, Gong N. miR-20a regulates inflammatory in osteoarthritis by targeting the IκBβ and regulates NK-κB signaling pathway activation, *Biochem Biophys Res Commun*. (2019) 518:632-7. doi: 10.1016/j.bbrc.2019.08.109

44. Jian W, Yunjuan T, Anquan S, Weiwei W, Yujie Z, Liqing H, et al. Effect of the interaction between MiR-200b-3p and DNMT3A on cartilage cells of osteoarthritis patients, *JOURNAL OF CELLULAR AND MOLECULAR MEDICINE*. (2017) 21:2308-16. doi: 10.1111/jcmm.13152

45. Zhang G, Sun Y, Wang Y, Liu R, Bao Y, Li Q. MiR-502-5p inhibits IL-1β-induced chondrocyte injury by targeting TRAF2, *Cell Immunol*. (2016) 302:50-7. doi: 10.1016/j.cellimm.2016.01.007

46. Makki MS, Haqqi TM. miR-139 modulates MCPIP1/IL-6 expression and induces apoptosis in human OA chondrocytes, *Exp Mol Med*. (2015) 47:e189. doi: 10.1038/emm.2015.66

47. Lingqiang C, Qin L, Jing W, Song J, Hongmei Z, Jun L, et al. MiR-29b-3p promotes chondrocyte apoptosis and facilitates the occurrence and development of osteoarthritis by targeting PGRN, *JOURNAL OF CELLULAR AND MOLECULAR MEDICINE*. (2017) 21:3347-59. doi: 10.1111/jcmm.13237

48. Minglei Q, Yuanxin S, Wei L. LINC00707 knockdown inhibits IL-1β-induced apoptosis and extracellular matrix degradation of osteoarthritis chondrocytes by the miR-330-5p/FSHR axis, *IMMUNOPHARMACOLOGY AND IMMUNOTOXICOLOGY*. (2022) 44:671-81. doi: 10.1080/08923973.2022.2076241

49. Lecheng Z, Cong S, Yuelei Z, Gang W, Zongsheng Y. Knockdown of hsa_circ_0134111 alleviates the symptom of osteoarthritis via sponging microRNA-224-5p, *Cell cycle (Georgetown, Tex.)*. (2021) 20:1052-66. doi: 10.1080/15384101.2021.1919838

50. Zhe C, Tao J, Yong L. AntimiR-30b Inhibits TNF-α Mediated Apoptosis and Attenuated Cartilage Degradation through Enhancing Autophagy, *Cellular physiology and biochemistry : international journal of experimental cellular physiology, biochemistry, and pharmacology*. (2016) 40:883-94. doi: 10.1159/000453147

51. Yuanxu G, Zixin M, Congshan J, Wei W, Jidong Y, Peng X, et al. Downregulation of HS6ST2 by miR-23b-3p enhances matrix degradation through p38 MAPK pathway in osteoarthritis, *Cell Death & Disease*. (2018) 9:699. doi: 10.1038/s41419-018-0729-0

52. Tao T, Yunkun Z, Hui W, Ke H. Downregulation of IRAK3 by miR-33b-3p relieves chondrocyte inflammation and apoptosis in an in vitro osteoarthritis model, *BIOSCIENCE BIOTECHNOLOGY AND BIOCHEMISTRY*. (2021) 85:545-52. doi: 10.1093/bbb/zbaa105

53. Yizhao Z, Sihong L, Ping C, Benyu Y, Junjun Y, Renfeng L, et al. MicroRNA-27b-3p inhibits apoptosis of chondrocyte in rheumatoid arthritis by targeting HIPK2, *Artificial Cells Nanomedicine and Biotechnology*. (2019) 47:1766-71. doi: 10.1080/21691401.2019.1607362

54. Zhang X, Liu X, Ni X, Feng P, Wang YU. Long non-coding RNA H19 modulates proliferation and apoptosis in osteoarthritis via regulating miR-106a-5p, *J Biosci*. (2019) 44:128 [pii]

55. Wen-Bin L, Gui-Shi L, Peng S, Ya-Nan L, Fu-Jiang Z. Long non-coding RNA HOTAIRM1-1 silencing in cartilage tissue induces osteoarthritis through microRNA-125b, *Exp Ther Med*. (2021) 22:933. doi: 10.3892/etm.2021.10365

56. Yubao L, Lupan L, Rui Z, Chuanyang W, Zhen W, Fuqing L. MSC-derived exosomes promote proliferation and inhibit apoptosis of chondrocytes via lncRNA-KLF3-AS1/miR-206/GIT1 axis in osteoarthritis, *Cell cycle (Georgetown, Tex.)*. (2018) 17:2411-22. doi: 10.1080/15384101.2018.1526603

57. Peng X, Xu Z, Jinpeng S, Yuhang Z, Weijian Q, Jianqiang L, et al. MicroRNA-613 alleviates IL-1β-induced injury in chondrogenic CHON-001 cells by targeting fibronectin 1, *American Journal of Translational Research*. (2020) 12:5308-19

58. Tu Y, Ma T, Wen T, Yang T, Xue L, Cai M, et al. MicroRNA-377-3p alleviates IL-1β-caused chondrocyte apoptosis and cartilage degradation in osteoarthritis in part by downregulating ITGA6, *Biochem Biophys Res Commun*. (2020) 523:46-53. doi: 10.1016/j.bbrc.2019.11.186

59. Masahiro H, Shun-Neng H, Anna R, Colin F, Louise A S. miR-29b inhibits TGF-β1-induced cell proliferation in articular chondrocytes, *Biochem Biophys Rep*. (2022) 29:101216. doi: 10.1016/j.bbrep.2022.101216

60. Shujun C, Bo L. MiR-128-3p Post-Transcriptionally Inhibits WISP1 to Suppress Apoptosis and Inflammation in Human Articular Chondrocytes via the PI3K/AKT/NF-κB Signaling Pathway, *CELL TRANSPLANTATION*. 29:963689720939131. doi: 10.1177/0963689720939131

61. Xu J, Fang X, Qin L, Wu Q, Zhan X. LncRNA PVT1 regulates biological function of osteoarthritis cells by regulating miR-497/AKT3 axis, *Medicine (Baltimore)*. (2022) 101:e31725. doi: 10.1097/MD.0000000000031725

62. Xue H, Yu P, Wang WZ, Niu YY, Li X. The reduced lncRNA NKILA inhibited proliferation and promoted apoptosis of chondrocytes via miR-145/SP1/NF-κB signaling in human osteoarthritis, *Eur Rev Med Pharmacol Sci*. (2020) 24:535-48. doi: 10.26355/eurrev_202001_20030

63. Xiuyun L, Yanhui Y, Fengxiang Y, Chuandong Y, Bing L, Jing L, et al. Knockdown of PVT1 inhibits IL-1β-induced injury in chondrocytes by regulating miR-27b-3p/TRAF3 axis, *International Immunopharmacology*. (2020) 79:106052. doi: 10.1016/j.intimp.2019.106052

64. Lei Z, Pin Z, Xiangyi S, Liwu Z, Jianning Z. Long non-coding RNA DANCR regulates proliferation and apoptosis of chondrocytes in osteoarthritis via miR-216a-5p-JAK2-STAT3 axis, *BIOSCIENCE REPORTS*. (2018) 38. doi: 10.1042/BSR20181228

65. Jin X, Xiaozhong Q, Ren D. MiR-24-3p attenuates IL-1β-induced chondrocyte injury associated with osteoarthritis by targeting BCL2L12, *Journal of Orthopaedic Surgery and Research*. (2021) 16:371. doi: 10.1186/s13018-021-02378-6

66. Li J, Huang J, Dai L, Yu D, Chen Q, Zhang X, et al. miR-146a, an IL-1β responsive miRNA, induces vascular endothelial growth factor and chondrocyte apoptosis by targeting Smad4, *Arthritis Res Ther*. (2012) 14:R75. doi: 10.1186/ar3798

67. Yanglin G, Guangchang W, Huazhong X. Long non-coding RNA ZNFX1 antisense 1 (ZFAS1) suppresses anti-oxidative stress in chondrocytes during osteoarthritis by sponging microRNA-1323, *Bioengineered*. (2022) 13:13188-200. doi: 10.1080/21655979.2022.2074770

68. Zhou C, He T, Chen L. LncRNA CASC19 accelerates chondrocytes apoptosis and proinflammatory cytokine production to exacerbate osteoarthritis development through regulating the miR-152-3p/DDX6 axis, *J Orthop Surg Res*. (2021) 16:399. doi: 10.1186/s13018-021-02543-x

69. Zhen J, Qing-Jun W. CircRNA-MSR Regulates LPS-Induced C28/I2 Chondrocyte Injury through miR-643/MAP2K6 Signaling Pathway, *Cartilage*. (2021) 13:785S-795S. doi: 10.1177/19476035211044826

70. Shang J, Li H, Wu B, Jiang N, Wang B, Wang D, et al. CircHIPK3 prevents chondrocyte apoptosis and cartilage degradation by sponging miR-30a-3p and promoting PON2, *Cell Prolif*. (2022) 55:e13285. doi: 10.1111/cpr.13285

71. Y-J Z, D-M J. LncRNA PART1 modulates chondrocyte proliferation, apoptosis, and extracellular matrix degradation in osteoarthritis via regulating miR-373-3p/SOX4 axis, *European Review for Medical and Pharmacological Sciences*. (2019) 23:8175-85. doi: 10.26355/eurrev_201910_19124

72. Ping X, Xuelong Z, Qian L. Modeling Osteoarthritis: MiR-16-5p Attenuates IL-1β Induced Chondrocyte Dysfunction by Targeting MAP2K1 through the MAPK Pathway, *ANNALS OF CLINICAL AND LABORATORY SCIENCE*. (2023) 53:248-58

73. Shi S, Lele Z, Qi W, Qian W, Dejian L, Wei S, et al. Targeting Cartilage miR-195/497 Cluster for Osteoarthritis Treatment Regulates the Circadian Clock, *GERONTOLOGY*. (2024) 70:59-75. doi: 10.1159/000534292

74. Ming C, Yue W. Downregulation of HMGB1 by miR-103a-3p Promotes Cell Proliferation, Alleviates Apoptosis and in Flammation in a Cell Model of Osteoarthritis, *Iranian Journal of Biotechnology*. (2020) 18:e2255. doi: 10.30498/IJB.2020.129470.2255

75. Xi P, Zhang CL, Wu SY, Liu L, Li WJ, Li YM. CircRNA circ-IQGAP1 Knockdown Alleviates Interleukin-1β-Induced Osteoarthritis Progression via Targeting miR-671-5p/TCF4, *Orthop Surg*. (2021) 13:1036-46. doi: 10.1111/os.12923

76. Shu Z, Yu'e L, Jian W, Yi W, Baitong W, Danjing Y, et al. ADSCs increase the autophagy of chondrocytes through decreasing miR-7-5p in Osteoarthritis rats by targeting ATG4A, *International Immunopharmacology*. (2023) 120:110390. doi: 10.1016/j.intimp.2023.110390

77. Chen H, Yang J, Tan Z. Upregulation of microRNA-9-5p inhibits apoptosis of chondrocytes through downregulating Tnc in mice with osteoarthritis following tibial plateau fracture, *J Cell Physiol*. (2019) 234:23326-36. doi: 10.1002/jcp.28900

78. Rijiang C, Yan T, Xiunian H, Wantao W, Fake L. circSLTM knockdown attenuates chondrocyte inflammation, apoptosis and ECM degradation in osteoarthritis by regulating the miR-515-5p/VAPB axis, *International Immunopharmacology*. (2024) 138:112435. doi: 10.1016/j.intimp.2024.112435

79. Yongqin J, Lei Z, Huiyu T. MicroRNA-149 improves osteoarthritis via repression of VCAM-1 and inactivation of PI3K/AKT pathway, *EXPERIMENTAL GERONTOLOGY*. (2023) 174:112103. doi: 10.1016/j.exger.2023.112103

80. Jinling Z, Fangyue C, Genxiang R, Zhi T, Binjie G. Hsa_circ_0005567 Activates Autophagy and Suppresses IL-1β-Induced Chondrocyte Apoptosis by Regulating miR-495, *Frontiers in Molecular Biosciences*. (2020) 7:216. doi: 10.3389/fmolb.2020.00216

81. He C, Zhao C, Liu S, Zhong Y, Liu L, Cai D. Down-regulation of MiR-138-5p Protects Chondrocytes ATDC5 and CHON-001 from IL-1 β-induced Inflammation <i>Via</i> Up-regulating SOX9, *CURRENT PHARMACEUTICAL DESIGN*. (2020) 25:4613-21. doi: 10.2174/1381612825666190905163046

82. Le P, Ming D, Yonggang M, Wei H, Fan L. miR-520c-3p regulates IL-1β-stimulated human chondrocyte apoptosis and cartilage degradation by targeting GAS2, *Journal of Orthopaedic Surgery and Research*. (2021) 16:347. doi: 10.1186/s13018-021-02466-7

83. Panyang S, Yute Y, Gang L, Weijie C, Junxing C, Qingxin W, et al. CircCDK14 protects against Osteoarthritis by sponging miR-125a-5p and promoting the expression of Smad2, *Theranostics*. (2020) 10:9113-31. doi: 10.7150/thno.45993

84. Ligang Q, Bo Y, Taoping C, Kang C, Zheng M, Yunfei W, et al. Circ_0022383 alleviates IL-1β-induced apoptosis, inflammation and extracellular matrix degeneration in osteoarthritis cell model by miR-3619-5p/SIRT1 axis, *International Immunopharmacology*. (2022) 112:109289. doi: 10.1016/j.intimp.2022.109289

85. Zhao S, Xiu G, Wang J, Wen Y, Lu J, Wu B, et al. Engineering exosomes derived from subcutaneous fat MSCs specially promote cartilage repair as miR-199a-3p delivery vehicles in Osteoarthritis, *J Nanobiotechnology*. (2023) 21:341. doi: 10.1186/s12951-023-02086-9

86. Zhilin C, Wenguang L, Xiaoyi Q, Haiyong B, Xiujiang S, Qian Y, et al. miR-296-5p inhibits IL-1β-induced apoptosis and cartilage degradation in human chondrocytes by directly targeting TGF-β1/CTGF/p38MAPK pathway, *Cell cycle (Georgetown, Tex.)*. (2020) 19:1443-53. doi: 10.1080/15384101.2020.1750813

87. Yuchang Z, Ping Z, Sen L, Xiangqian M, Huaqi W. CircSCAPER knockdown attenuates IL-1β-induced chondrocyte injury by miR-127-5p/TLR4 axis in osteoarthritis, *AUTOIMMUNITY*. (2022) 55:577-86. doi: 10.1080/08916934.2022.2103798

88. Jian-Lin Z, Shuang D, Hong-Song F, Xian-Jin D, Hao P, Qiong-Jie H. Circular RNA circANKRD36 regulates Casz1 by targeting miR-599 to prevent osteoarthritis chondrocyte apoptosis and inflammation, *JOURNAL OF CELLULAR AND MOLECULAR MEDICINE*. (2021) 25:120-31. doi: 10.1111/jcmm.15884

89. Shixing M, Aobo Z, Xiaole L, Shizhou Z, Shaopeng L, Haoming Z, et al. MiR-21-5p regulates extracellular matrix degradation and angiogenesis in TMJOA by targeting Spry1, *ARTHRITIS RESEARCH & THERAPY*. (2020) 22:99. doi: 10.1186/s13075-020-2145-y

90. Wang B, Ji D, Xing W, Li F, Huang Z, Zheng W, et al. miR-142-3p and HMGB1 Are Negatively Regulated in Proliferation, Apoptosis, Migration, and Autophagy of Cartilage Endplate Cells, *Cartilage*. (2021) 13:592S-603S. doi: 10.1177/19476035211012444

91. Zhibin Z, Jun M, Jiajia L, Aimin C, Lei Z. Circular RNA CircCDH13 contributes to the pathogenesis of osteoarthritis via CircCDH13/miR-296-3p/PTEN axis, *JOURNAL OF CELLULAR PHYSIOLOGY*. (2021) 236:3521-35. doi: 10.1002/jcp.30091

92. Yawei Z, Hengheng Z, Baitong L. Circ_0110251 overexpression alleviates IL-1β-induced chondrocyte apoptosis and extracellular matrix degradation by regulating miR-3189-3p/SPRY1 axis in osteoarthritis, *AUTOIMMUNITY*. (2022) 55:168-78. doi: 10.1080/08916934.2022.2027917

93. Xue H, Tu Y, Ma T, Wen T, Yang T, Xue L, et al. miR-93-5p attenuates IL-1β-induced chondrocyte apoptosis and cartilage degradation in osteoarthritis partially by targeting TCF4, *Bone*. (2019) 123:129-36. doi: 10.1016/j.bone.2019.03.035

94. Xianwei H, Kun G, Shuaihua L, Rongbo W. LncRNA HOTTIP leads to osteoarthritis progression via regulating miR-663a/ Fyn-related kinase axis, *BMC MUSCULOSKELETAL DISORDERS*. (2021) 22:67. doi: 10.1186/s12891-020-03861-7

95. Xiao O, Yunzhi D, Li Y, Feng X, Xiaowei Y, Xingyong L, et al. Circ_SPG11 plays contributing effects on IL-1β-induced chondrocyte apoptosis and ECM degradation via miR-665 inhibition-mediated GREM1 upregulation, *Clinical immunology (Orlando, Fla.)*. (2021) 233:108889. doi: 10.1016/j.clim.2021.108889

96. Dan M, Mingsong W, Jiying W, Xiangwen Z, Lan Y, Fang C. MicroRNA-101a-3p could be involved in the pathogenesis of temporomandibular joint osteoarthritis by mediating UBE2D1 and FZD4, *JOURNAL OF ORAL PATHOLOGY & MEDICINE*. (2021) 50:236-43. doi: 10.1111/jop.13131

97. Yunping Z, Zandong Z, Liang Y, Jie Y. MiR-485-3p promotes proliferation of osteoarthritis chondrocytes and inhibits apoptosis via Notch2 and the NF-κB pathway, *IMMUNOPHARMACOLOGY AND IMMUNOTOXICOLOGY*. (2021) 43:370-9. doi: 10.1080/08923973.2021.1918150

98. Jinsoo S, Eun-Heui J, Dongkyun K, Keun Young K, Churl-Hong C, Eun-Jung J. MicroRNA-222 regulates MMP-13 via targeting HDAC-4 during osteoarthritis pathogenesis, *BBA clinical*. (2015) 3:79-89. doi: 10.1016/j.bbacli.2014.11.009

99. Jialei H, Zi W, Yue P, Jia M, Xiaoyan M, Xia Q, et al. MiR-26a and miR-26b mediate osteoarthritis progression by targeting FUT4 via NF-κB signaling pathway, *INTERNATIONAL JOURNAL OF BIOCHEMISTRY & CELL BIOLOGY*. (2018) 94:79-88. doi: 10.1016/j.biocel.2017.12.003

100. Yuru J, Yuxiang L, Hengyong X, Zhi H, Ranran D, Yuxin Z, et al. miR-206a-3p suppresses the proliferation and differentiation of chicken chondrocytes in tibial dyschondroplasia by targeting BMP6, *POULTRY SCIENCE*. (2024) 103:103534. doi: 10.1016/j.psj.2024.103534

101. Guojian F, Feng Y, Jianli Z. Depletion of circ_0128846 ameliorates interleukin-1β-induced human chondrocyte apoptosis and inflammation through the miR-940/PTPN12 pathway, *International Immunopharmacology*. (2022) 110:108996. doi: 10.1016/j.intimp.2022.108996

102. Yongsheng Z, Chengcheng D, Pengcheng X, Yiting L, Piao Z, Zhenglin Z, et al. Sox9-Increased miR-322-5p Facilitates BMP2-Induced Chondrogenic Differentiation by Targeting Smad7 in Mesenchymal Stem Cells, *Stem Cells International*. (2021) 2021:9778207. doi: 10.1155/2021/9778207

103. Xindie Z, Lifeng J, Guoming F, Haoyu Y, Lidong W, Yong H, et al. Role of the ciRS-7/miR-7 axis in the regulation of proliferation, apoptosis and inflammation of chondrocytes induced by IL-1β, *International Immunopharmacology*. (2019) 71:233-40. doi: 10.1016/j.intimp.2019.03.037

104. Heyan S, Zhenwei L, Nannan L, Tao X, Kongzu H, Yubao S, et al. Long Non-coding RNA SNHG7 Suppresses Inflammation and Apoptosis of Chondrocytes Through Inactivating of p38 MAPK Signaling Pathway in Osteoarthritis, *MOLECULAR BIOTECHNOLOGY*. (2023) . doi: 10.1007/s12033-023-00856-2

105. Xiao-Fei S, Yi C, Qi-Rong D, Min-Qian Z. MicroRNA-675-3p regulates IL-1β-stimulated human chondrocyte apoptosis and cartilage degradation by targeting GNG5, *BIOCHEMICAL AND BIOPHYSICAL RESEARCH COMMUNICATIONS*. (2020) 527:458-65. doi: 10.1016/j.bbrc.2020.04.044

106. Dawei Z, Xiaorui C, Jun L, Guangyue Z. MiR-210 inhibits NF-κB signaling pathway by targeting DR6 in osteoarthritis, *Sci Rep*. (2015) 5:12775. doi: 10.1038/srep12775

107. Jing W, Lingqing C, Song J, Jun L, Hongmei Z, Hong Z, et al. Altered expression of microRNA-98 in IL-1β-induced cartilage degradation and its role in chondrocyte apoptosis, *Molecular Medicine Reports*. (2017) 16:3208-16. doi: 10.3892/mmr.2017.7028

108. Jin X, Yaozeng X. The lncRNA MEG3 downregulation leads to osteoarthritis progression via miR-16/SMAD7 axis, *Cell Biosci*. (2017) 7:69. doi: 10.1186/s13578-017-0195-x

109. Zhichao L, Jin W, Jing Y. TUG1 knockdown promoted viability and inhibited apoptosis and cartilage ECM degradation in chondrocytes via the miR-17-5p/FUT1 pathway in osteoarthritis, *Exp Ther Med*. (2020) 20:154. doi: 10.3892/etm.2020.9283

110. Zhongqiang Z, Bo Y, Shuping Z, Junxing W. CircRNA circ_SEC24A upregulates DNMT3A expression by sponging miR-26b-5p to aggravate osteoarthritis progression, *International Immunopharmacology*. (2021) 99:107957. doi: 10.1016/j.intimp.2021.107957

111. Mingli F, Lin J, Jingbo C, Shuai A, Jiang H, Qi Y. Circ_0020093 ameliorates IL-1β-induced apoptosis and extracellular matrix degradation of human chondrocytes by upregulating SPRY1 via targeting miR-23b, *MOLECULAR AND CELLULAR BIOCHEMISTRY*. (2021) 476:3623-33. doi: 10.1007/s11010-021-04186-2

112. S-T G, Y-M Y, L-P W, Z-M L, J-X L. LncRNA GAS5 induces chondrocyte apoptosis by down-regulating miR-137, *European Review for Medical and Pharmacological Sciences*. (2020) 24:10984-91. doi: 10.26355/eurrev_202011_23582

113. Hua Z, XiaoBing X, BenGen Z, JianFa C, YouQiang S, ShuangXiao Z, et al. Circular RNA SLTM as a miR-421-competing endogenous RNA to mediate HMGB2 expression stimulates apoptosis and inflammation in arthritic chondrocytes, *JOURNAL OF BIOCHEMICAL AND MOLECULAR TOXICOLOGY*. (2023) 37:e23306. doi: 10.1002/jbt.23306

114. Dongjie H, Ling L, Ran T. Hsa_circ_0007482 Promotes Proliferation and Differentiation of Chondrocytes in Knee Osteoarthritis, *Cartilage*. (2024) :19476035241250198. doi: 10.1177/19476035241250198

115. Kai Z, Yan Z, DongDong L, MingZhong X, HuaCai J, KaiQuan Z, et al. MiR-29a-3p mediates phosphatase and tensin homolog and inhibits osteoarthritis progression, *FUNCTIONAL & INTEGRATIVE GENOMICS*. (2024) 24:54. doi: 10.1007/s10142-024-01327-w

116. Helal E, Poulami D, Anirudh S, Sayaka N, Evgeny R, Carolen Y, et al. MicroRNA-34a-5p Promotes Joint Destruction During Osteoarthritis, *Arthritis & rheumatology (Hoboken, N.J.)*. (2021) 73:426-39. doi: 10.1002/art.41552

117. Hao X, Changrong D, Cuicui G, Shuai X, Yingzhen W, Bing L, et al. Suppression of CRLF1 promotes the chondrogenic differentiation of bone marrow-derived mesenchymal stem and protects cartilage tissue from damage in osteoarthritis via activation of miR-320, *Molecular medicine (Cambridge, Mass.)*. (2021) 27:116. doi: 10.1186/s10020-021-00369-1

118. Gu W, Shi Z, Song G, Zhang H. MicroRNA-199-3p up-regulation enhances chondrocyte proliferation and inhibits apoptosis in knee osteoarthritis via DNMT3A repression, *Inflamm Res*. (2021) 70:171-82. doi: 10.1007/s00011-020-01430-1

119. P C, Y F, M D, J L, H C, Q M, et al. MiR-15b is a key regulator of proliferation and apoptosis of chondrocytes from patients with condylar hyperplasia by targeting IGF1, IGF1R and BCL2, *Osteoarthritis and Cartilage*. (2019) 27:336-46. doi: 10.1016/j.joca.2018.09.010

120. Yang H, Wu D, Li H, Chen N, Shang Y. Downregulation of microRNA-448 inhibits IL-1β-induced cartilage degradation in human chondrocytes via upregulation of matrilin-3, *Cell Mol Biol Lett*. (2018) 23:7. doi: 10.1186/s11658-018-0072-6

121. Renqi J, Huili G, Fei C, Wentao Z, Tao S, Zirui Y. Circ_DHRS3 positively regulates GREM1 expression by competitively targeting miR-183-5p to modulate IL-1β-administered chondrocyte proliferation, apoptosis and ECM degradation, *International Immunopharmacology*. (2021) 91:107293. doi: 10.1016/j.intimp.2020.107293

122. Xin Z, Feng-Chao Z, Yong P, Dong-Ya L, Sheng-Cheng Y, Shao-Song S, et al. Downregulation of miR-221-3p contributes to IL-1β-induced cartilage degradation by directly targeting the SDF1/CXCR4 signaling pathway, *Journal of molecular medicine (Berlin, Germany)*. (2017) 95:615-27. doi: 10.1007/s00109-017-1516-6

123. Xiaodong L, Feng G, Weikang W, Jinglong Y. Expression of miR-204 in patients with osteoarthritis and its damage to chondrocytes, *JOURNAL OF MUSCULOSKELETAL & NEURONAL INTERACTIONS*. (2020) 20:265-71

124. Guohua Lü, Lei L, Bing W, Lei K. LINC00623/miR-101/HRAS axis modulates IL-1β-mediated ECM degradation, apoptosis and senescence of osteoarthritis chondrocytes, *Aging*. (2020) 12:3218-37. doi: 10.18632/aging.102801

125. Lei L, Guohua L, Bing W, Lei K. The role of lncRNA XIST/miR-211 axis in modulating the proliferation and apoptosis of osteoarthritis chondrocytes through CXCR4 and MAPK signaling, *BIOCHEMICAL AND BIOPHYSICAL RESEARCH COMMUNICATIONS*. (2018) 503:2555-62. doi: 10.1016/j.bbrc.2018.07.015

126. Xiao-Chen L, Liang X, Yu-Li C, Zhi-Yong Z, E-Nuo D, Shui S. MiR-1207-5p/CX3CR1 axis regulates the progression of osteoarthritis via the modulation of the activity of NF-κB pathway, *International Journal of Rheumatic Diseases*. (2020) 23:1057-65. doi: 10.1111/1756-185X.13898

127. Feiri H, Zhongliang S, Jie Y, Xizhen Z, Yaozeng X. Downregulation of lncRNA NEAT1 interacts with miR-374b-5p/PGAP1 axis to aggravate the development of osteoarthritis, *Journal of Orthopaedic Surgery and Research*. (2023) 18:670. doi: 10.1186/s13018-023-04147-z

128. Bingzhe H, Haichi Y, Yingzhi L, Wei Z, Xiaoning L. Upregulation of long noncoding TNFSF10 contributes to osteoarthritis progression through the miR-376-3p/FGFR1 axis, *JOURNAL OF CELLULAR BIOCHEMISTRY*. (2019) 120:19610-20. doi: 10.1002/jcb.29267

129. Xiang L, Yuanyuan W, Zhuyun C, Qi Z, Lexiang L, Peiliang F. Exosomes from human umbilical cord mesenchymal stem cells inhibit ROS production and cell apoptosis in human articular chondrocytes via the miR-100-5p/NOX4 axis, *CELL BIOLOGY INTERNATIONAL*. (2021) 45:2096-106. doi: 10.1002/cbin.11657

130. Laiya L, Jingyi W, Aoyuan F, Pei W, Runzhi C, Laibing L, et al. Synovial mesenchymal stem cell-derived extracellular vesicles containing microRN555A-26a-5p ameliorate cartilage damage of osteoarthritis, *JOURNAL OF GENE MEDICINE*. (2021) 23:e3379. doi: 10.1002/jgm.3379

131. Mei-Li M, Jin-Mei J, Xiao-Ping L, Li-Hu X. miR-144-3p aggravated cartilage injury in rheumatoid arthritis by regulating BMP2/PI3K/Akt axis, *Modern Rheumatology*. (2022) 32:1064-76. doi: 10.1093/mr/roab105

132. Fengjin T, Dongbo W, Zhongkai Y. The Fibroblast-Like Synoviocyte Derived Exosomal Long Non-coding RNA H19 Alleviates Osteoarthritis Progression Through the miR-106b-5p/TIMP2 Axis, *INFLAMMATION*. (2020) 43:1498-509. doi: 10.1007/s10753-020-01227-8

133. Fanyou N, Shaobo Z, Hui G, Yu D. NEAT1/miR-146a-3p/TrkB/ShcB axis regulates the development and function of chondrocyte, *Cell cycle (Georgetown, Tex.)*. (2021) 20:2174-94. doi: 10.1080/15384101.2021.1974787

134. Jinsoo S, Dongkyun K, Churl-Hong C, Eun-Jung J. MicroRNA-9 regulates survival of chondroblasts and cartilage integrity by targeting protogenin, *Cell Communication and Signaling*. (2013) 11:66. doi: 10.1186/1478-811X-11-66

135. Yang S, Sanmao S, Mengjun L, Ang D. Inhibition of miR-182-5p Targets FGF9 to Alleviate Osteoarthritis, *Analytical Cellular Pathology*. (2023) 2023:5911546. doi: 10.1155/2023/5911546

136. Lin Y, Zhang L, Ji M, Shen S, Chen Y, Wu S, et al. MiR-653-5p drives osteoarthritis pathogenesis by modulating chondrocyte senescence, *Arthritis Res Ther*. (2024) 26:111. doi: 10.1186/s13075-024-03334-5

137. Y H, H X L, D X, X X, X X. The Anti-Inflammatory Effect of miR-140-3p in BMSCs-Exosomes on Osteoarthritis, *Acta Chirurgiae Orthopaedicae et Traumatologiae Cechoslovaca*. (2023) 90:267-76

138. Jizhe Y, Yushuang Q, Naxin Z. Knockdown of Circ_SLC39A8 protects against the progression of osteoarthritis by regulating miR-591/IRAK3 axis, *Journal of Orthopaedic Surgery and Research*. (2021) 16:170. doi: 10.1186/s13018-021-02323-7

139. Baiyang D, Shujuan X, Xiumin S, Jianming G, Wenlei N, Hongguang X. miR-18a-3p Encourages Apoptosis of Chondrocyte in Osteoarthritis via HOXA1 Pathway, *Curr Mol Pharmacol*. (2020) 13:328-41. doi: 10.2174/1874467213666200204143740

140. Kai X, Zhen M, Xin-Miao X, Mo-Hong D, Qing-Gong M, Wei F, et al. LncRNA PVT1 induces chondrocyte apoptosis through upregulation of TNF-α in synoviocytes by sponging miR-211-3p, *MOLECULAR AND CELLULAR PROBES*. (2020) 52:101560. doi: 10.1016/j.mcp.2020.101560

141. Yusheng L, Wenqing X, Yizi Z, Hengzhen L, Zeqin W, Chen W, et al. The miR-548d-5p/SP1 signaling axis regulates chondrocyte proliferation and inflammatory responses in osteoarthritis, *International Immunopharmacology*. (2022) 110:109029. doi: 10.1016/j.intimp.2022.109029

142. Zhenye G, Huan W, Feng Z, Min L, Feida W, Mingming K, et al. Exosomal circ-BRWD1 contributes to osteoarthritis development through the modulation of miR-1277/TRAF6 axis, *ARTHRITIS RESEARCH & THERAPY*. (2021) 23:159. doi: 10.1186/s13075-021-02541-8

143. Zhe N, Xifu S, Guolin T, Lei N. Expression of miR-206 in Human Knee Articular Chondrocytes and Effects of miR-206 on Proliferation and Apoptosis of Articular Chondrocytes, *AMERICAN JOURNAL OF THE MEDICAL SCIENCES*. (2018) 355:240-6. doi: 10.1016/j.amjms.2017.11.003

144. Li Z, Cheng J, Liu J. Baicalin Protects Human OA Chondrocytes Against IL-1β-Induced Apoptosis and ECM Degradation by Activating Autophagy via MiR-766-3p/AIFM1 Axis, *Drug Des Devel Ther*. (2020) 14:2645-55. doi: 10.2147/DDDT.S255823

145. Jipeng L, Zhongxiong W, Ying X. Knockdown of long noncoding RNA HOTAIR inhibits osteoarthritis chondrocyte injury by miR-107/CXCL12 axis, *Journal of Orthopaedic Surgery and Research*. (2021) 16:410. doi: 10.1186/s13018-021-02547-7

146. Guiqiang M, Xuehui Z, Huige H, Hui S, Lihui W, Ting Z, et al. Bax Targeted by miR-29a Regulates Chondrocyte Apoptosis in Osteoarthritis, *Biomed Res Int*. (2019) 2019:1434538. doi: 10.1155/2019/1434538

147. Weikai Z, Peng C, Weihua H, Weifeng Y, Fengjing G, Anmin C, et al. Inhibition of microRNA-384-5p alleviates osteoarthritis through its effects on inhibiting apoptosis of cartilage cells via the NF-κB signaling pathway by targeting SOX9, *CANCER GENE THERAPY*. (2018) 25:326-38. doi: 10.1038/s41417-018-0029-y

148. Zhang B, Sun M, Wang J, Ma C, Hao T, Liu G, et al. MiR-671 ameliorates the progression of osteoarthritis in vitro and in vivo, *Pathol Res Pract*. (2019) 215:152423. doi: 10.1016/j.prp.2019.04.015

149. Jianwei X, Rongsheng W, Weijian Z, Xu C, Zhizhong Y. Circular RNA CSNK1G1 promotes the progression of osteoarthritis by targeting the miR‑4428/FUT2 axis, *INTERNATIONAL JOURNAL OF MOLECULAR MEDICINE*. (2021) 47:232-42. doi: 10.3892/ijmm.2020.4772

150. Maoxun L, Fei G, Hongyu C. MiR-30b-5p Influences Chronic Exercise Arthritic Injury by Targeting Hoxa1, *INTERNATIONAL JOURNAL OF SPORTS MEDICINE*. (2021) 42:1199-208. doi: 10.1055/a-1342-7872

151. Yuanmin Z, Longfei M, Chengqun W, Lina W, Yanxia G, Guodong W. Long noncoding RNA LINC00461 induced osteoarthritis progression by inhibiting miR-30a-5p, *Aging*. (2020) 12:4111-23. doi: 10.18632/aging.102839

152. Shaojian C, Zhihuan L, Xiaguang C. Andrographolide mitigates cartilage damage via miR-27-3p-modulated matrix metalloproteinase13 repression, *JOURNAL OF GENE MEDICINE*. (2020) 22:e3187. doi: 10.1002/jgm.3187

153. Peng Y, Zhanhu M, Daihao W, Pengcheng G, Mei M, Haibo Y. miR-3960 from Mesenchymal Stem Cell-Derived Extracellular Vesicles Inactivates SDC1/Wnt/<i>β</i>-Catenin Axis to Relieve Chondrocyte Injury in Osteoarthritis by Targeting PHLDA2, *Stem Cells International*. (2022) 2022:9455152. doi: 10.1155/2022/9455152

154. Li S, Liu J, Chen L. MiR-330-5p inhibits intervertebral disk degeneration via targeting CILP, *J Orthop Surg Res*. (2021) 16:440. doi: 10.1186/s13018-021-02582-4

155. Sujeong P, Jinjoo O, Yong-Il K, Seong-Kyu C, Churl-Hong C, Eun-Jung J. Suppression of ABCD2 dysregulates lipid metabolism via dysregulation of miR-141:ACSL4 in human osteoarthritis, *CELL BIOCHEMISTRY AND FUNCTION*. (2018) 36:366-76. doi: 10.1002/cbf.3356

156. Hai-Chao D, Pei-Nan L, Chang-Jian C, Xin X, Hong Z, Gang L, et al. Sinomenine Attenuates Cartilage Degeneration by Regulating miR-223-3p/NLRP3 Inflammasome Signaling, *INFLAMMATION*. (2019) 42:1265-75. doi: 10.1007/s10753-019-00986-3

157. Luping T, Jianbo D, Guangju Z, Zhihai L. LncRNA‑p21 promotes chondrocyte apoptosis in osteoarthritis by acting as a sponge for miR‑451, *Molecular Medicine Reports*. (2018) 18:5295-301. doi: 10.3892/mmr.2018.9506

158. Xin L, Liangliang L, Hongbo Z, Yan S, Ziyu C, Xiaofeng F, et al. MiR-146b accelerates osteoarthritis progression by targeting alpha-2-macroglobulin, *Aging*. (2019) 11:6014-28. doi: 10.18632/aging.102160

159. Changjian C, Peng Y, Shengxiong H, Xuegang S, Baowen L. Circular RNA-9119 protects IL-1β-treated chondrocytes from apoptosis in an osteoarthritis cell model by intercepting the microRNA-26a/PTEN axis, *LIFE SCIENCES*. (2020) 256:117924. doi: 10.1016/j.lfs.2020.117924

160. Jing M, Lei Z. MiR-320a upregulation improves IL-1β-induced osteoarthritis via targeting the DAZAP1 and MAPK pathways, *Journal of Orthopaedic Surgery and Research*. (2023) 18:541. doi: 10.1186/s13018-023-03984-2

161. Haibin Z, Xilei L, Yusheng L, Xucheng Y, Runzhi L, Haoyi W, et al. CREB Ameliorates Osteoarthritis Progression Through Regulating Chondrocytes Autophagy <i>via</i> the miR-373/METTL3/TFEB Axis, *Frontiers in Cell and Developmental Biology*. (2021) 9:778941. doi: 10.3389/fcell.2021.778941

162. Hanyu L, Yixin Y, Shuanji O, Yong Q, Guitao L, Hebei H, et al. The silencing of miR-199a-5p protects the articular cartilage through MAPK4 in osteoarthritis, *Ann Transl Med*. (2022) 10:601. doi: 10.21037/atm-22-2057

163. Chen K, Fang H, Xu N. LncRNA LOXL1-AS1 is transcriptionally activated by JUND and contributes to osteoarthritis progression via targeting the miR-423-5p/KDM5C axis, *Life Sci*. (2020) 258:118095. doi: 10.1016/j.lfs.2020.118095

164. Wang Z, Zhou N, Wang W, Yu Y, Xia L, Li N. HDAC2 interacts with microRNA-503-5p to regulate SGK1 in osteoarthritis, *Arthritis Res Ther*. (2021) 23:78. doi: 10.1186/s13075-020-02373-y

165. Tiansheng Z, Jishang H, Jinliang L, Qingluo Z, Tong L, Qiang X, et al. Long non-coding RNA HOTAIRincreased mechanical stimulation-induced apoptosis by regulating microRNA-221/BBC3 axis in C28/I2 cells, *Bioengineered*. (2021) 12:10734-44. doi: 10.1080/21655979.2021.2003129

166. Xiaokun L, Yu L, Huimin C, Yuancheng P, Ran L, Shunyou C. miR-335-5P contributes to human osteoarthritis by targeting HBP1, *Exp Ther Med*. (2021) 21:109. doi: 10.3892/etm.2020.9541

167. Xing Z, Chao-Ran H, Sheng P, Yong P, Ye-Shuai C, Guo-Chun Z, et al. Long non-coding RNA SNHG15 is a competing endogenous RNA of miR-141-3p that prevents osteoarthritis progression by upregulating BCL2L13 expression, *International Immunopharmacology*. (2020) 83:106425. doi: 10.1016/j.intimp.2020.106425

168. Jinliang W, Xiaofei L, Songtao C, Jingtao S, Shaohua W, Xuan W. Blocking HOTAIR protects human chondrocytes against IL-1β-induced cell apoptosis, ECM degradation, inflammatory response and oxidative stress via regulating miR-222-3p/ADAM10 axis, *International Immunopharmacology*. (2021) 98:107903. doi: 10.1016/j.intimp.2021.107903

169. Lv H, Liu P, Hu H, Li X, Li P. MiR-98-5p plays suppressive effects on IL-1β-induced chondrocyte injury associated with osteoarthritis by targeting CASP3, *J Orthop Surg Res*. (2024) 19:239. doi: 10.1186/s13018-024-04628-9

170. Qing L, Mingjie W, Guofang F, Kuangwen L, Wengang C, Liang L, et al. MicroRNA‑186‑5p downregulation inhibits osteoarthritis development by targeting MAPK1, *Molecular Medicine Reports*. (2021) 23. doi: 10.3892/mmr.2021.11892

171. Rozi R, Zhou Y, Rong K, Chen P. miR-124-3p sabotages lncRNA MALAT1 stability to repress chondrocyte pyroptosis and relieve cartilage injury in osteoarthritis, *J Orthop Surg Res*. (2022) 17:453. doi: 10.1186/s13018-022-03334-8

172. Xiao H, Lili D. Potential of miR-25-3p in protection of chondrocytes: emphasis on osteoarthritis, *FOLIA HISTOCHEMICA ET CYTOBIOLOGICA*. (2021) 59:30-9. doi: 10.5603/FHC.a2021.0004

173. Yan X, Liang D, Shizhang L, Yuanyuan Y, Zhi Q, Liang S. Long intergenic non-protein coding RNA 00707 regulates chondrocyte apoptosis and proliferation in osteoarthritis by serving as a sponge for microRNA-199-3p, *Bioengineered*. (2022) 13:11137-45. doi: 10.1080/21655979.2022.2061287

174. Shuying S, Yizheng W, Junxin C, Ziang X, Kangmao H, Gangliang W, et al. CircSERPINE2 protects against osteoarthritis by targeting miR-1271 and ETS-related gene, *ANNALS OF THE RHEUMATIC DISEASES*. (2019) 78:826-36. doi: 10.1136/annrheumdis-2018-214786

175. Miao Z, Zhiqiang W, Baojie L, Fengyi S, Anzhong C, Mingzhi G. Identification of microRNA‑363‑3p as an essential regulator of chondrocyte apoptosis in osteoarthritis by targeting NRF1 through the p53‑signaling pathway, *Molecular Medicine Reports*. (2020) 21:1077-88. doi: 10.3892/mmr.2020.10940

176. Pan H, Dai H, Wang L, Lin S, Tao Y, Zheng Y, et al. MicroRNA-410-3p modulates chondrocyte apoptosis and inflammation by targeting high mobility group box 1 (HMGB1) in an osteoarthritis mouse model, *BMC Musculoskelet Disord*. (2020) 21:486. doi: 10.1186/s12891-020-03489-7

177. Xiao-Bo W, Feng-Chao Z, Lin-Hong Y, Jin-Long T, Zheng-Ya Z, Yong P, et al. MicroRNA-21-5p as a novel therapeutic target for osteoarthritis, *Rheumatology (Oxford, England)*. (2019) . doi: 10.1093/rheumatology/kez102

178. Jinsoo S, Chihyun A, Churl-Hong C, Eun-Jung J. A long non-coding RNA, GAS5, plays a critical role in the regulation of miR-21 during osteoarthritis, *JOURNAL OF ORTHOPAEDIC RESEARCH*. (2014) 32:1628-35. doi: 10.1002/jor.22718

179. Yu L, Zhang X, Liu X, Li G, Chen M, Liu Z, et al. CircTMOD3 promotes lipopolysaccharide-induced chondrocyte apoptosis in osteoarthritis by sponging miR-27a, *J Bone Miner Metab*. (2022) 40:415-21. doi: 10.1007/s00774-022-01310-0

180. Jian W, Xiang W, Xu D, Tao H, Dengxin S, Hairong T. EZH2 is associated with cartilage degeneration in osteoarthritis by promoting SDC1 expression via histone methylation of the microRNA-138 promoter, *LABORATORY INVESTIGATION*. (2021) 101:600-11. doi: 10.1038/s41374-021-00532-6

181. Jun L, Ming-Liang J, Xue-Jun Z, Pei-Liang S, Hao W, Chen W, et al. MicroRNA-218-5p as a Potential Target for the Treatment of Human Osteoarthritis, *MOLECULAR THERAPY*. (2017) 25:2676-88. doi: 10.1016/j.ymthe.2017.08.009

182. Meng-Ya L, Yong-Hui Y, Xian W, Ze-Yu S, Man L, Shuai X, et al. [Effect of needle-knife on chondrocyte apoptosis of knee joint in rabbits with knee osteoarthritis based on CircSERPINE2-miR-1271-5P-ERG axis], *Zhongguo zhen jiu = Chinese acupuncture & moxibustion*. (2023) 43:447-53. doi: 10.13703/j.0255-2930.20220411-k0001

183. Chengyuan Z, Ye L, Feng Y, Shilin J. Circular RNA CCDC66 Regulates Osteoarthritis Progression by Targeting miR-3622b-5p, *GERONTOLOGY*. (2022) 68:431-41. doi: 10.1159/000520325

184. Yingchun C, Lin Z, Ernan L, Genai Z, Yu H, Wei Y, et al. Long-chain non-coding RNA HOTAIR promotes the progression of osteoarthritis via sponging miR-20b/PTEN axis, *LIFE SCIENCES*. (2020) 253:117685. doi: 10.1016/j.lfs.2020.117685

185. Dongyun L, Xiaoying W, Tengda Y, Lin Z, Lirui F, Mingxing Z, et al. LncRNA MINCR attenuates osteoarthritis progression via sponging miR-146a-5p to promote BMPR2 expression, *Cell cycle (Georgetown, Tex.)*. (2022) 21:2417-32. doi: 10.1080/15384101.2022.2099191

186. Yanping X, Jianwei L, Yanhui S, Yuanshen L, Qingshan L, Xuan C. Downregulation of miR-3680-3p inhibits the progression of osteoarthritis via targeting OGG1, *ARCHIVES OF GERONTOLOGY AND GERIATRICS*. 100:104626. doi: 10.1016/j.archger.2022.104626

187. Jia H, Jia Z, Xiaochen F, Zhe L. Electro-acupuncture modulated miR-214 expression to prevent chondrocyte apoptosis and reduce pain by targeting BAX and TRPV4 in osteoarthritis rats, *Brazilian journal of medical and biological research = Revista brasileira de pesquisas medicas e biologicas*. (2024) 57:e13238. doi: 10.1590/1414-431X2024e13238

188. LuLu Y, ChangJun S, YuanYuan L, DongSheng L, TianTian X, XuQin G, et al. Down-regulation of long noncoding RNA HULC inhibits the inflammatory response in ankylosing spondylitis by reducing miR-556-5p-mediated YAP1 expression, *Journal of Orthopaedic Surgery and Research*. (2023) 18:551. doi: 10.1186/s13018-023-04003-0

189. Yue Y, Zhibo S, Feng L, Yuanzhang B, Fei W. FGD5-AS1 Inhibits Osteoarthritis Development by Modulating miR-302d-3p/TGFBR2 Axis, *Cartilage*. (2021) 13:1412S-1420S. doi: 10.1177/19476035211003324

190. Yao L, Ting F, Yi Z, Long-Jie Y, Bai-Bai W, Jian G, et al. Micro-223 Promotes Diabetic Osteoarthritis Progression by Regulating Cartilage Degeneration and Subchondral Bone Remodeling, *Cartilage*. (2023) :19476035231210631. doi: 10.1177/19476035231210631

191. Yachen L, Junjun D, Weicheng L, Jie L. Exosomal miR-93-5p regulated the progression of osteoarthritis by targeting ADAMTS9, *Open medicine (Warsaw, Poland)*. (2023) 18:20230668. doi: 10.1515/med-2023-0668

192. Zhongmeng Y, Yuxing T, Qing Z, Huading L, Guoyong X. Down-regulation of microRNA-23b aggravates LPS-induced inflammatory injury in chondrogenic ATDC5 cells by targeting PDCD4, *Iranian Journal of Basic Medical Sciences*. (2018) 21:529-35. doi: 10.22038/IJBMS.2018.25856.6364

193. Feng L, Xiangyang L, Yue Y, Zhibo S, Shuang D, Zhongping J, et al. NEAT1/miR-193a-3p/SOX5 axis regulates cartilage matrix degradation in human osteoarthritis, *CELL BIOLOGY INTERNATIONAL*. (2020) 44:947-57. doi: 10.1002/cbin.11291

194. Ningbo L, Yongsheng W, Xuejian W. Knockdown of Circ_0037658 Alleviates IL-1β-Induced Osteoarthritis Progression by Serving as a Sponge of miR-665 to Regulate ADAMTS5, *Frontiers in Genetics*. (2022) 13:886898. doi: 10.3389/fgene.2022.886898

195. Taitao S, Jian Y, Liang H, Shuo T, Bin X, Xianbin G, et al. Knockdown of Long Non-Coding RNA RP11-445H22.4 Alleviates LPS-Induced Injuries by Regulation of MiR-301a in Osteoarthritis, *Cellular physiology and biochemistry : international journal of experimental cellular physiology, biochemistry, and pharmacology*. (2018) 45:832-43. doi: 10.1159/000487175

196. Wang C, Wang L, Guan X, Yue C. MiR-4303 relieves chondrocyte inflammation by targeting ASPN in osteoarthritis, *J Orthop Surg Res*. (2021) 16:618. doi: 10.1186/s13018-021-02731-9

197. Xue-Peng L, Xu W, Shang-Quan W, Gang S, Ying-Chun Z, He Y, et al. Differentiation Antagonizing Non-protein Coding RNA Knockdown Alleviates Lipopolysaccharide-Induced Inflammatory Injury and Apoptosis in Human Chondrocyte Primary Chondrocyte Cells Through Upregulating miRNA-19a-3p, *Orthopaedic Surgery*. (2021) 13:276-84. doi: 10.1111/os.12845

198. Dadihanc T, Zhang Y, Li GQ, Zhou HK, Huang J, Zhang X, et al. CircRNA SEC24A promotes osteoarthritis through miR-107-5p/CASP3 axis, *Regen Ther*. (2024) 26:60-70. doi: 10.1016/j.reth.2024.04.011

199. Yueqi Z, Guobin Q, Yuheng Y, Chenzhong W, Zhe W, Chang J, et al. Exosomes derived from bone marrow mesenchymal stem cells pretreated with decellularized extracellular matrix enhance the alleviation of osteoarthritis through miR-3473b/phosphatase and tensin homolog axis, *JOURNAL OF GENE MEDICINE*. (2023) 25:e3510. doi: 10.1002/jgm.3510

200. Yuan Q, Yu-Ping Z, Jing W, Li-Gang J, Jia-Xin D, Dong-Bao Z, et al. Downregulated microRNA-135a ameliorates rheumatoid arthritis by inactivation of the phosphatidylinositol 3-kinase/AKT signaling pathway via phosphatidylinositol 3-kinase regulatory subunit 2, *JOURNAL OF CELLULAR PHYSIOLOGY*. (2019) 234:17663-76. doi: 10.1002/jcp.28390

201. Yiming W, Aoyuan F, Liangyu L, Zhangyi P, Min M, Shulin L, et al. Exosome modification to better alleviates endoplasmic reticulum stress induced chondrocyte apoptosis and osteoarthritis, *BIOCHEMICAL PHARMACOLOGY*. (2022) 206:115343. doi: 10.1016/j.bcp.2022.115343

202. Nan K, Zhang Y, Zhang X, Li D, Zhao Y, Jing Z, et al. Exosomes from miRNA-378-modified adipose-derived stem cells prevent glucocorticoid-induced osteonecrosis of the femoral head by enhancing angiogenesis and osteogenesis via targeting miR-378 negatively regulated suppressor of fused (Sufu), *Stem Cell Res Ther*. (2021) 12:331. doi: 10.1186/s13287-021-02390-x

203. Chen R, Ye B, Xie H, Huang Y, Wu Z, Wu H, et al. miR-129-3p alleviates chondrocyte apoptosis in knee joint fracture-induced osteoarthritis through CPEB1, *J Orthop Surg Res*. (2020) 15:552. doi: 10.1186/s13018-020-02070-1

204. Feiri H, Zhongliang S, Jie Y, Xizhen Z, Yaozeng X. Knocking-down long non-coding RNA LINC01094 prohibits chondrocyte apoptosis via regulating microRNA-577/metal-regulatory transcription factor 1 axis, *Journal of orthopaedic surgery (Hong Kong)*. 32:10225536241254588. doi: 10.1177/10225536241254588

205. Yongsheng W, Ningbo L, Xuejian W. Circular RNA_0003800 exacerbates IL-1β-induced chondrocyte injury via miR-197-3p/SOX5 axis, *International Immunopharmacology*. (2023) 115:109643. doi: 10.1016/j.intimp.2022.109643

206. Q W, Z-H Y, X-B M, X-H T. Low expression of CircRNA HIPK3 promotes osteoarthritis chondrocyte apoptosis by serving as a sponge of miR-124 to regulate SOX8, *European Review for Medical and Pharmacological Sciences*. (2020) 24:7937-45. doi: 10.26355/eurrev_202008_22476

207. Yang X, Song Y, Sun Y, Wang M, Xiang Y. Down-regulation of miR-361-5p promotes the viability, migration and tube formation of endothelial progenitor cells via targeting FGF1, *Biosci Rep*. (2020) 40:BSR20200557. doi: 10.1042/BSR20200557

208. Dong J, Shigao C, Pengcheng K, Tengfei L, Xun L, Jiongzhe X, et al. microRNA-105-5p protects against chondrocyte injury, extracellular matrix degradation, and osteoarthritis progression by targeting SPARCL1, *HISTOLOGY AND HISTOPATHOLOGY*. (2024) 39:483-96. doi: 10.14670/HH-18-654

209. W-F W, S-Y L, Z-F Q, Z-H L, H-R D, W-J Z. MiR-145 targeting BNIP3 reduces apoptosis of chondrocytes in osteoarthritis through Notch signaling pathway, *European Review for Medical and Pharmacological Sciences*. (2020) 24:8263-72. doi: 10.26355/eurrev_202008_22622

210. Shaobo Z, Yu D, Hui G, Kaiyuan H, Zhongjie N. miR-877-5p alleviates chondrocyte dysfunction in osteoarthritis models via repressing FOXM1, *JOURNAL OF GENE MEDICINE*. (2020) 22:e3246. doi: 10.1002/jgm.3246

211. Yingchi Z, Rui L, Xiaojian H, Enzhi Y, Yong Y, Chengla Y, et al. Circular RNA MELK Promotes Chondrocyte Apoptosis and Inhibits Autophagy in Osteoarthritis by Regulating MYD88/NF-<i>κ</i>B Signaling Axis through MicroRNA-497-5p, *Contrast Media Mol Imaging*. (2022) 2022:7614497. doi: 10.1155/2022/7614497

212. Peng S, Ying L, Jie R, Qingqing L, Haobo S, Junqing J, et al. Overexpression of miR-532-5p restrains oxidative stress response of chondrocytes in nontraumatic osteonecrosis of the femoral head by inhibiting ABL1, *Open medicine (Warsaw, Poland)*. (2024) 19:20240943. doi: 10.1515/med-2024-0943

213. Zhou M, Liu B, Ye HM, Hou JN, Huang YC, Zhang P, et al. ROS-induced imbalance of the miR-34a-5p/SIRT1/p53 axis triggers chronic chondrocyte injury and inflammation, *Heliyon*. (2024) 10:e31654. doi: 10.1016/j.heliyon.2024.e31654

214. Zhang Y, Peng X, Song W, Sun Y, Wang L, Li Q, et al. [Effects of microRNA-140 gene transfection with nucleus localization signal linked nucleic kinase substrate short peptide conjugated chitosan on rabbit articular chondrocytes], *Zhongguo Xiu Fu Chong Jian Wai Ke Za Zhi*. (2017) 31:1256-61. doi: 10.7507/1002-1892.201705088

215. Mingjun Q, Yanhua X, Guanghua T, Xiaoxu W, Peiguan H, Liang H. Synovial mesenchymal stem cell-derived exosomal miR-485-3p relieves cartilage damage in osteoarthritis by targeting the NRP1-mediated PI3K/Akt pathway: Exosomal miR-485-3p relieves cartilage damage, *Heliyon*. (2024) 10:e24042. doi: 10.1016/j.heliyon.2024.e24042

216. Wang Y, Bai L. Resveratrol inhibits apoptosis by increase in the proportion of chondrocytes in the S phase of cell cycle in articular cartilage of ACLT plus Mmx rats, *Saudi J Biol Sci*. (2019) 26:839-44. doi: 10.1016/j.sjbs.2017.04.010

217. Zhi-Xi D, Peng H, Chao T, Qing L, Shuang-Qing L, Ze-Ling L, et al. MicroRNA-15a-5p Regulates the Development of Osteoarthritis by Targeting PTHrP in Chondrocytes, *Biomed Res Int*. (2019) 2019:3904923. doi: 10.1155/2019/3904923

218. Hongxing L, Zhiling L, Yigang P, Yang C, Lin M, Yong L, et al. MicroRNA-375 exacerbates knee osteoarthritis through repressing chondrocyte autophagy by targeting ATG2B, *Aging*. (2020) 12:7248-61. doi: 10.18632/aging.103073

219. Libo Z, Deping C, Penghui L, Lei C, Yucheng S. miR-132-3p participates in the pathological mechanism of temporomandibular joint osteoarthritis by targeting PTEN, *ARCHIVES OF ORAL BIOLOGY*. (2022) 142:105511. doi: 10.1016/j.archoralbio.2022.105511

220. Wei C, Feilong W, Jiangtao W, Fuyu C, Ting C. The Molecular Mechanism of Long Non-Coding RNA MALAT1-Mediated Regulation of Chondrocyte Pyroptosis in Ankylosing Spondylitis, *MOLECULES AND CELLS*. (2022) 45:365-75. doi: 10.14348/molcells.2022.2081

221. Qiang W, Pei-Yan H, Jun-Guo W, Tie-Qi Z, Ling-Feng L, Liang-Da H, et al. miR-219a-5p inhibits the pyroptosis in knee osteoarthritis by inactivating the NLRP3 signaling via targeting FBXO3, *Environ Toxicol*. (2022) 37:2673-82. doi: 10.1002/tox.23627

222. Linlin C, Pinpin J, Hang W, Lingan H, Gaige W, Xianda C, et al. MiR-1 is a critical regulator of chondrocyte proliferation and hypertrophy by inhibiting Indian hedgehog pathway during postnatal endochondral ossification in miR-1 overexpression transgenic mice, *BONE*. (2022) 165:116566. doi: 10.1016/j.bone.2022.116566

223. Jiashu Y, Ming Z, Dawei Y, Yunfei M, Yuting T, Mengying X, et al. m<sup>6</sup>A-mediated upregulation of AC008 promotes osteoarthritis progression through the miR-328-3p‒AQP1/ANKH axis, *EXPERIMENTAL AND MOLECULAR MEDICINE*. (2021) 53:1723-34. doi: 10.1038/s12276-021-00696-7

224. Gen L, Lijun X, Xiaoyun L, Lisha M, Jihui Z. miR-155 inhibits chondrocyte pyroptosis in knee osteoarthritis by targeting SMAD2 and inhibiting the NLRP3/Caspase-1 pathway, *Journal of Orthopaedic Surgery and Research*. (2022) 17:48. doi: 10.1186/s13018-021-02886-5

225. Jin-Mei J, Mei-Li M, Xiao-Ping L, Li-Hu X. MiR-144-3p induced by SP1 promotes IL-1β-induced pyroptosis in chondrocytes via PTEN/PINK1/Parkin axis, *AUTOIMMUNITY*. (2022) 55:21-31. doi: 10.1080/08916934.2021.1983802

226. Lei Z, Jianjun Q, Jixiang S, Shaoyang L, Hanlin Z. MicroRNA-140-5p represses chondrocyte pyroptosis and relieves cartilage injury in osteoarthritis by inhibiting cathepsin B/Nod-like receptor protein 3, *Bioengineered*. (2021) 12:9949-64. doi: 10.1080/21655979.2021.1985342

227. Jiatian Q, Peiliang F, Shiao L, Xiang L, Yancheng C, Zhenen L. miR-107 affects cartilage matrix degradation in the pathogenesis of knee osteoarthritis by regulating caspase-1, *Journal of Orthopaedic Surgery and Research*. (2021) 16:40. doi: 10.1186/s13018-020-02121-7

228. Tianjun Z, Zengqiao Z, Xiaoshen H, Dongyi H, Wei F. Role of Long Intergenic Nonprotein-Coding RNA 00511 in Nod-Like Receptor Protein Pyrin Domain 3-Induced Chondrocyte Pyroptosis via the MicroRNA-9-5p/FUT1 Axis, *JOURNAL OF MICROBIOLOGY AND BIOTECHNOLOGY*. (2024) 34:1511-21. doi: 10.4014/jmb.2312.12014

229. Liu W, Liu A, Li X, Sun Z, Sun Z, Liu Y, et al. Dual-engineered cartilage-targeting extracellular vesicles derived from mesenchymal stem cells enhance osteoarthritis treatment via miR-223/NLRP3/pyroptosis axis: Toward a precision therapy, *Bioact Mater*. (2023) 30:169-83. doi: 10.1016/j.bioactmat.2023.06.012

230. Jianhang W, Tao S. Mir-25-3p in extracellular vesicles from fibroblast-like synoviocytes alleviates pyroptosis of chondrocytes in knee osteoarthritis, *JOURNAL OF BIOENERGETICS AND BIOMEMBRANES*. (2023) 55:365-80. doi: 10.1007/s10863-023-09964-9

231. Honggang X, Bin X. BMSC-Derived Exosomes Ameliorate Osteoarthritis by Inhibiting Pyroptosis of Cartilage via Delivering miR-326 Targeting HDAC3 and STAT1//NF-<i>κ</i>B p65 to Chondrocytes, *Mediators Inflamm*. (2021) 2021:9972805. doi: 10.1155/2021/9972805

232. Jiangyi W, Liang K, Cheng C, Junjun Y, Wei-Nan Z, Tao L, et al. miR-100-5p-abundant exosomes derived from infrapatellar fat pad MSCs protect articular cartilage and ameliorate gait abnormalities via inhibition of mTOR in osteoarthritis, *BIOMATERIALS*. (2019) 206:87-100. doi: 10.1016/j.biomaterials.2019.03.022

233. Shuang Q, Benjuan L, Yanshuai M, Xueqin W, Lina Z, Xiao H, et al. MicroRNA-153-3p increases autophagy in sevoflurane-preconditioned mice to protect against ischaemic/reperfusion injury after knee arthroplasty, *JOURNAL OF CELLULAR AND MOLECULAR MEDICINE*. (2020) 24:5330-40. doi: 10.1111/jcmm.15188

234. Mingzhuang H, Yijian Z, Xinfeng Z, Tao L, Huilin Y, Xi C, et al. Kartogenin prevents cartilage degradation and alleviates osteoarthritis progression in mice via the miR-146a/NRF2 axis, *Cell Death & Disease*. (2021) 12:483. doi: 10.1038/s41419-021-03765-x

235. Chen C, Shaoxiong M, Bo Y, Wen L, Xiao Y, Liuxun L, et al. MiR-27a promotes the autophagy and apoptosis of IL-1β treated-articular chondrocytes in osteoarthritis through PI3K/AKT/mTOR signaling, *Aging*. (2019) 11:6371-84. doi: 10.18632/aging.102194

236. Shile C, Zhigang N, Jiarui C, Hao P. Circ_0136474 promotes the progression of osteoarthritis by sponging mir-140-3p and upregulating MECP2, *JOURNAL OF MOLECULAR HISTOLOGY*. (2023) 54:1-12. doi: 10.1007/s10735-022-10100-x

237. Weidong H, Ye C. Inhibition of miR-20 promotes proliferation and autophagy in articular chondrocytes by PI3K/AKT/mTOR signaling pathway, *Biomedicine & pharmacotherapy = Biomedecine & pharmacotherapie*. (2018) 97:607-15. doi: 10.1016/j.biopha.2017.10.152

238. Fei X, Yong-Ming L, Hai-Bin W, Ying-Chun S. miR-31-5p/SOX4 Axis Affects Autophagy and Apoptosis of Chondrocytes by Regulating Extracellular Regulated Protein Kinase/Mechanical Target of Rapamycin Kinase Signalling, *PATHOBIOLOGY*. (2022) 89:63-73. doi: 10.1159/000519006

239. Hui H, Huasong S, Lingxiao J, Dingmei Z, Hui W, Jing L, et al. Autophagy activated by GR/miR-421-3p/mTOR pathway as a compensatory mechanism participates in chondrodysplasia induced by prenatal caffeine exposure in male fetal rats, *TOXICOLOGY LETTERS*. (2024) 397:141-50. doi: 10.1016/j.toxlet.2024.05.010

240. Ganesan J, Ramanujam D, Sassi Y, Ahles A, Jentzsch C, Werfel S, et al. MiR-378 controls cardiac hypertrophy by combined repression of mitogen-activated protein kinase pathway factors, *Circulation*. (2013) 127:2097-106. doi: 10.1161/CIRCULATIONAHA.112.000882

241. Gu M, Haiwen Y, Kai S, Donghong Z, Julin Z, Hao W, et al. Circular RNA RHOT1 Regulates miR-142-5p/CCND1 to Participate in Chondrocyte Autophagy and Proliferation in Osteoarthritis, *Journal of Immunology Research*. (2022) 2022:4370873. doi: 10.1155/2022/4370873

242. Bin H, Dianming J. HOTAIR-induced apoptosis is mediated by sponging miR-130a-3p to repress chondrocyte autophagy in knee osteoarthritis, *CELL BIOLOGY INTERNATIONAL*. (2020) 44:524-35. doi: 10.1002/cbin.11253

243. Jing Z, Zhenzhen Z, Qing L, Qijin L, Jiemei L, Lixia Y, et al. CircPan3 Promotes the Ghrelin System and Chondrocyte Autophagy by Sponging miR-667-5p During Rat Osteoarthritis Pathogenesis, *Frontiers in Cell and Developmental Biology*. (2021) 9:719898. doi: 10.3389/fcell.2021.719898

244. Ko JY, Wang FS, Lian WS, Fang HC, Kuo SJ. Cartilage-specific knockout of miRNA-128a expression normalizes the expression of circadian clock genes (CCGs) and mitigates the severity of osteoarthritis, *Biomed J*. (2024) 47:100629. doi: 10.1016/j.bj.2023.100629

245. Tianwen S, Fei W, Gaojian H, Zhizhou L. Salvianolic acid B activates chondrocytes autophagy and reduces chondrocyte apoptosis in obese mice via the KCNQ1OT1/miR-128-3p/SIRT1 signaling pathways, *Nutrition & Metabolism*. (2022) 19:53. doi: 10.1186/s12986-022-00686-0

246. Zi W, Jialei H, Yue P, Yujia S, Liqun J, Xia Q, et al. miR-140-5p/miR-149 Affects Chondrocyte Proliferation, Apoptosis, and Autophagy by Targeting FUT1 in Osteoarthritis, *INFLAMMATION*. (2018) 41:959-71. doi: 10.1007/s10753-018-0750-6

247. Yang F, Huang R, Ma H, Zhao X, Wang G. miRNA-411 Regulates Chondrocyte Autophagy in Osteoarthritis by Targeting Hypoxia-Inducible Factor 1 alpha (HIF-1α), *Med Sci Monit*. (2020) 26:e921155. doi: 10.12659/MSM.921155

248. Huasong S, Bin L, Dingmei Z, Hui H, Hangyuan H, Jiayong Z, et al. Autophagy inhibition mediated by intrauterine miR-1912-3p/CTSD programming participated in the susceptibility to osteoarthritis induced by prenatal dexamethasone exposure in male adult offspring rats, *FASEB JOURNAL*. (2023) 37:e23011. doi: 10.1096/fj.202300022RR

249. Yan S, Wang M, Zhao J, Zhang H, Zhou C, Jin L, et al. MicroRNA-34a affects chondrocyte apoptosis and proliferation by targeting the SIRT1/p53 signaling pathway during the pathogenesis of osteoarthritis, *Int J Mol Med*. (2016) 38:201-9. doi: 10.3892/ijmm.2016.2618

250. Thomas G W, Madhu B, Navdeep K, Vasilios M, Jason D, Shabana Amanda A. Characterization of miR-335-5p and miR-335-3p in human osteoarthritic tissues, *ARTHRITIS RESEARCH & THERAPY*. (2023) 25:105. doi: 10.1186/s13075-023-03088-6

251. Lian WS, Ko JY, Wu RW, Sun YC, Chen YS, Wu SL, et al. MicroRNA-128a represses chondrocyte autophagy and exacerbates knee osteoarthritis by disrupting Atg12, *Cell Death Dis*. (2018) 9:919. doi: 10.1038/s41419-018-0994-y

252. Shuai Z, Zhe J. Bone Mesenchymal Stem Cell-Derived Extracellular Vesicles Containing Long Noncoding RNA NEAT1 Relieve Osteoarthritis, *Oxid Med Cell Longev*. (2022) 2022:5517648. doi: 10.1155/2022/5517648

253. Xia Q, Wang Q, Lin F, Wang J. miR-125a-5p-abundant exosomes derived from mesenchymal stem cells suppress chondrocyte degeneration via targeting E2F2 in traumatic osteoarthritis, *Bioengineered*. (2021) 12:11225-38. doi: 10.1080/21655979.2021.1995580

254. Xie SC, Yang L, Shu T, Liu Q, Wang W. miR-149-5p mitigates tumor necrosis factor-α-induced chondrocyte apoptosis by inhibiting TRADD, *Arch Med Sci*. (2024) 20:602-11. doi: 10.5114/aoms.2020.92324

255. Ming Y, DaWei Z, Min Y. Saikosaponin D alleviates inflammatory response of osteoarthritis and mediates autophagy via elevating microRNA-199-3p to target transcription Factor-4, *Journal of Orthopaedic Surgery and Research*. (2024) 19:151. doi: 10.1186/s13018-024-04607-0

256. Xindie Z, Jin L, Yuanshuai Z, Zhicheng Y, Haoyu Y, Dong L, et al. Down-regulated ciRS-7/up-regulated miR-7 axis aggravated cartilage degradation and autophagy defection by PI3K/AKT/mTOR activation mediated by IL-17A in osteoarthritis, *Aging*. (2020) 12:20163-83. doi: 10.18632/aging.103731

257. Huang J, Peng J, Cao G, Lu S, Liu L, Li Z, et al. Hypoxia-Induced MicroRNA-429 Promotes Differentiation of MC3T3-E1 Osteoblastic Cells by Mediating ZFPM2 Expression, *Cell Physiol Biochem*. (2016) 39:1177-86. doi: 10.1159/000447824

258. Haoyi C, Fangjing C, Fangqiong H, Yifan L, Meixing Z, Qi Z, et al. MicroRNA-224-5p nanoparticles balance homeostasis via inhibiting cartilage degeneration and synovial inflammation for synergistic alleviation of osteoarthritis, *Acta Biomaterialia*. (2023) 167:401-15. doi: 10.1016/j.actbio.2023.06.010

259. Jun W, Xiaopeng L, Xiang G, Congcong W, Zezhong L, Xiaoguang L, et al. MicroRNA-34a-5p promotes the progression of osteoarthritis secondary to developmental dysplasia of the hip by restraining SESN2-induced autophagy, *JOURNAL OF ORTHOPAEDIC RESEARCH*. (2024) 42:66-77. doi: 10.1002/jor.25639

260. Chao L, Hongyi J, Zhongnan L, Tian X, Weidan W, Chihao L, et al. MiR-146b-5p enriched bioinspired exosomes derived from fucoidan-directed induction mesenchymal stem cells protect chondrocytes in osteoarthritis by targeting TRAF6, *JOURNAL OF NANOBIOTECHNOLOGY*. (2023) 21:486. doi: 10.1186/s12951-023-02264-9

261. Clément V, Abderrahim B, Romain C, Amandine D, Emilie B, Jérôme E L, et al. Tumor Suppressive Role of miR-342-5p in Human Chondrosarcoma Cells and 3D Organoids, *INTERNATIONAL JOURNAL OF MOLECULAR SCIENCES*. (2021) 22. doi: 10.3390/ijms22115590

262. Ni Z, Kuang L, Chen H, Xie Y, Zhang B, Ouyang J, et al. The exosome-like vesicles from osteoarthritic chondrocyte enhanced mature IL-1β production of macrophages and aggravated synovitis in osteoarthritis, *Cell Death Dis*. (2019) 10:522. doi: 10.1038/s41419-019-1739-2

263. Hu G, Zhao X, Wang C, Geng Y, Zhao J, Xu J, et al. MicroRNA-145 attenuates TNF-α-driven cartilage matrix degradation in osteoarthritis via direct suppression of MKK4, *Cell Death Dis*. (2017) 8:e3140. doi: 10.1038/cddis.2017.522

264. S D, O A, Y M, F F, M K L. MicroRNA-155 suppresses autophagy in chondrocytes by modulating expression of autophagy proteins, *Osteoarthritis and Cartilage*. (2016) 24:1082-91. doi: 10.1016/j.joca.2016.01.005

265. Jian-Feng L, Lian-Gang Q, Xiao-Bo Z, Yi-Xin S. LncRNA RMRP knockdown promotes proliferation and inhibits apoptosis in osteoarthritis chondrocytes by miR-206/CDK9 axis, *PHARMAZIE*. (2020) 75:500-4. doi: 10.1691/ph.2020.0591

266. Deokha K, Jinsoo S, Eun-Jung J. BNIP3-Dependent Mitophagy via PGC1α Promotes Cartilage Degradation, *Cells*. (2021) 10. doi: 10.3390/cells10071839

267. Mingmin S, Menghao S, Cong W, Yue S, Yangxin W, Shigui Y. Therapeutic Potential of POU3F3, a Novel Long Non-coding RNA, Alleviates the Pathogenesis of Osteoarthritis by Regulating the miR-29a- 3p/FOXO3 Axis, *CURRENT GENE THERAPY*. (2022) 22:427-38. doi: 10.2174/1566523222666220309150722

268. Yong L, Fangchang Y, Yuxi S, Xiliang G. miR-17-5p and miR-19b-3p prevent osteoarthritis progression by targeting EZH2, *Exp Ther Med*. (2020) 20:1653-63. doi: 10.3892/etm.2020.8887

269. Wen-Peng X, Teng M, Yan-Chen L, Xiang-Peng W, Rong-Xiu B, Wei-Guo W, et al. [Cangxi Tongbi Capsules promote chondrocyte autophagy by regulating circRNA＿0008365/miR-1271/p38 MAPK pathway to inhibit development of knee osteoarthritis], *Zhongguo Zhong yao za zhi = Zhongguo zhongyao zazhi = China journal of Chinese materia medica*. (2023) 48:4843-51. doi: 10.19540/j.cnki.cjcmm.20230510.708

270. Qian Y, Bei Z, Qi H, Yuan Z, Xian-Bo P. microRNA-206 is required for osteoarthritis development through its effect on apoptosis and autophagy of articular chondrocytes via modulating the phosphoinositide 3-kinase/protein kinase B-mTOR pathway by targeting insulin-like growth factor-1, *JOURNAL OF CELLULAR BIOCHEMISTRY*. (2019) 120:5287-303. doi: 10.1002/jcb.27803

271. Chongtao Z, Bin C, Xu H, Weiyuan L, Shengyu W, Xun Z, et al. LncRNA MEG3 suppresses erastin-induced ferroptosis of chondrocytes via regulating miR-885-5p/SLC7A11 axis, *MOLECULAR BIOLOGY REPORTS*. (2024) 51:139. doi: 10.1007/s11033-023-09095-9

272. Ming Z, Chenjun Z, Kai S, Gang L, Lei L, Jian H, et al. miR-1 Inhibits the Ferroptosis of Chondrocyte by Targeting CX43 and Alleviates Osteoarthritis Progression, *Journal of Immunology Research*. (2023) 2023:2061071. doi: 10.1155/2023/2061071

273. Dexin W, Yu F, Liang L, Wensuo L, Lei W, Liwei Y, et al. Upregulating miR-181b promotes ferroptosis in osteoarthritic chondrocytes by inhibiting SLC7A11, *BMC MUSCULOSKELETAL DISORDERS*. (2023) 24:862. doi: 10.1186/s12891-023-07003-7

274. Chunlei H, Zhaogan Z, Yadong Y, Shanshan Y, Qiang W, Xunzhi L, et al. Silencing of CircTRIM25/miR-138-5p/CREB1 axis promotes chondrogenesis in osteoarthritis, *AUTOIMMUNITY*. (2024) 57:2361749. doi: 10.1080/08916934.2024.2361749

275. Ruina K, Lianmei J, Yafei P, Dongbao Z, Jie G. Exosomes from osteoarthritic fibroblast-like synoviocytes promote cartilage ferroptosis and damage via delivering microRNA-19b-3p to target SLC7A11 in osteoarthritis, *Front Immunol*. (2023) 14:1181156. doi: 10.3389/fimmu.2023.1181156

276. Sheng B, Li X, Zhou L, Zhou J, Guan R, Zhang X. Targeting miR-10a-5p/IL-6R axis for reducing IL-6-induced cartilage cell ferroptosis, *EXPERIMENTAL AND MOLECULAR PATHOLOGY*. (2021) 118:104570. doi: 10.1016/j.yexmp.2020.104570
